# Characterization and comprehensive genome analysis of novel bacteriophage, vB_Kpn_ZCKp20p, with lytic and anti-biofilm potential against clinical multidrug-resistant *Klebsiella pneumoniae*

**DOI:** 10.3389/fcimb.2023.1077995

**Published:** 2023-01-23

**Authors:** Bishoy Maher Zaki, Nada A. Fahmy, Ramy Karam Aziz, Reham Samir, Ayman El-Shibiny

**Affiliations:** ^1^ Department of Microbiology and Immunology, Faculty of Pharmacy, October University for Modern Sciences and Arts (MSA), 6th of October, Giza, Egypt; ^2^ Center for Microbiology and Phage Therapy, Biomedical Sciences, Zewail City of Science and Technology, Giza, Egypt; ^3^ Department of Microbiology and Immunology, Faculty of Pharmacy, Cairo University, Cairo, Egypt; ^4^ Center for Genome and Microbiome Research, Cairo University, Cairo, Egypt; ^5^ Microbiology and Immunology Research Program, Children’s Cancer Hospital Egypt, Cairo, Egypt; ^6^ Faculty of Environmental Agricultural Sciences, Arish University, Arish, Egypt

**Keywords:** antimicrobial resistance (AMR), genomics, phage therapy, phylogenetic analysis, siphovirus, phage proteomic tree, genome-based taxonomy

## Abstract

**Introduction:**

The rise of infections by antibiotic-resistant bacterial pathogens is alarming. Among these, *Klebsiella pneumoniae* is a leading cause of death by hospital-acquired infections, and its multidrug-resistant strains are flagged as a global threat to human health, which necessitates finding novel antibiotics or alternative therapies. One promising therapeutic alternative is the use of virulent bacteriophages, which specifically target bacteria and coevolve with them to overcome potential resistance. Here, we aimed to discover specific bacteriophages with therapeutic potential against multiresistant *K. pneumoniae* clinical isolates.

**Methods and Results:**

Out of six bacteriophages that we isolated from urban and medical sewage, phage vB_Kpn_ZCKp20p had the broadest host range and was thus characterized in detail. Transmission electron microscopy suggests vB_Kpn_ZCKp20p to be a tailed phage of the siphoviral morphotype. *In vitro* evaluation indicated a high lytic efficiency (30 min latent period and burst size of ∼100 PFU/cell), and extended stability at temperatures up to 70°C and a wide range of (2-12) pH. Additionally, phage vB_Kpn_ZCKp20p possesses antibiofilm activity that was evaluated by the crystal violet assay and was not cytotoxic to human skin fibroblasts. The whole genome was sequenced and annotated, uncovering one tRNA gene and 33 genes encoding proteins with assigned functions out of 85 predicted genes. Furthermore, comparative genomics and phylogenetic analysis suggest that vB_Kpn_ZCKp20p most likely represents a new species, but belongs to the same genus as *Klebsiella* phages ZCKP8 and 6691. Comprehensive genomic and bioinformatics analyses substantiate the safety of the phage and its strictly lytic lifestyle.

**Conclusion:**

Phage vB_Kpn_ZCKp20p is a novel phage with potential to be used against biofilm-forming *K. pneumoniae* and could be a promising source for antibacterial and antibiofilm products, which will be individually studied experimentally in future studies.

## 1 Introduction

Infections by antimicrobial-resistant pathogens are a serious threat to global health and are expected to cause more mortality than cancer by 2050, if no radically different intervention strategy is implemented (O’Neill, 2016; Murray et al., 2022). In particular, a list of six bacterial pathogens, dubbed the ESKAPE pathogens (*Enterococcus faecium*, *Staphylococcus aureus*, *Klebsiella pneumoniae*, *Acinetobacter baumannii*, *Pseudomonas aeruginosa*, and *Enterobacter* spp.), are expected to become resistant to all known antibiotics and are commonly spread in hospitals (Rice, 2008).

*K. pneumoniae*, one of the ESKAPE pathogens, is a normal inhabitant of the gastrointestinal mucosa of animals and humans; however, it can be life-threatening as it causes opportunistic infections, including pneumonia, bacteremia, urinary tract infections, wound infections, meningitis, and neonatal sepsis (Wyres and Holt, 2018). Globally, *K. pneumoniae* is behind 11% of hospital-acquired pneumonia cases (Ashurst and Dawson, 2022). New variants of *K. pneumoniae* are responsible for severe community-acquired infections (CAIs) that have been developed seriously since the 1990s. These CAIs are highly invasive and commonly complicated by a metastatic spread in healthy individuals; these infections include pyogenic liver abscess, endophthalmitis, bacteremia, and meningitis (Ko et al., 2002; Shon et al., 2013).

Being both an environmental free-living and animal host-associated bacterium, *K. pneumoniae* has great potential for acquiring and transmitting antimicrobial resistance (AMR) genes, mainly through horizontal gene transfer. Consequently, *K. pneumoniae* strains were among the first in which horizontally acquired extended-spectrum beta-lactamase (ESBL), quinolone resistance, carbapenemases, and—lately—colistin resistance genes were detected (De Oliveira et al., 2020; Mancuso et al., 2021). This makes multi-drug resistant (MDR) *K. pneumoniae* strains a primary hub for disseminating various AMR genes to Gram-negative pathogens (Wyres and Holt, 2018).

The Centers for Disease Control and Prevention (CDC) categorized carbapenem-resistant *Enterobacteriaceae* as an urgent threat (CDC: Centers for Disease Control Prevention, 2019). Carbapenem-resistant *K. pneumoniae* (CRKP), causing bloodstream infections and pneumonia, can lead to a mortality rate > 40% (De Oliveira et al., 2020), and in Egypt, MDR *K. pneumoniae* is a critical concern with commonly isolated carbapenem- and colistin-resistant strains (Hamza et al., 2016; Attia et al., 2019; Mills and Lee, 2019; Abdelwahab et al., 2021; Elmonir et al., 2021).

Pathogenic *K. pneumoniae* can firmly form biofilms and attach to biotic and abiotic surfaces, which shield the bacteria, thus contributing to their AMR, defense against host immune response, and invasive infections (Wang et al., 2020). These biofilms are also crucial for medical device colonization, which leads to device-associated nosocomial infections (Wu et al., 2019), reported to represent 25% of all hospital-acquired infections (often referred to as HAIs) in the United States acute care hospitals (Magill et al., 2014). In Egyptian hospitals, *Klebsiella* spp. were the most frequently isolated pathogens from HAIs (Fathy et al., 2013; Talaat et al., 2016; Hassan et al., 2020).

MDR *K. pneumoniae* strains are a warning sign for the post-antibiotic crisis and a vital target for developing alternative therapies to resolve the antimicrobial resistance and the dry pipeline of new antimicrobial compounds. One such alternative is bacteriophage therapy.

Bacteriophages (phages in short) are the natural predators of bacteria and have been considered for over 100 years to treat bacterial infections, yet the rise of the antibiotics industry largely masked them. Today, they are reconsidered for treating MDR infections owing to their high specificity and ability to replicate and coevolve with their hosts. In contrast to antibiotics, phage therapy has an activity that is limited to the site of infection, and it neither leads to dysbacteriosis nor toxicity to eukaryotic cells (Burrowes et al., 2011; Li et al., 2022). Additionally, some phages can disrupt bacterial biofilms, for example, through the production of tail-associated depolymerases, which degrade the polysaccharides of the bacterial biofilm to reach and infect the bacterial cell. The depolymerase activity against the biofilm makes the bacteria vulnerable and unprotected from host immunity and antimicrobial agents (Latka et al., 2017; Majkowska-Skrobek et al., 2018; Gordillo Altamirano and Barr, 2019). The pipeline for phage isolation is less elaborate and more economical than novel antimicrobial discovery pipelines, which makes phage discovery affordable for academic research laboratories (Belete, 2019; Principi et al., 2019).

Here, we aimed to isolate local phages with high specificity, high lytic potential and antibiofilm activity against MDR *K. pneumoniae* isolated from Egyptian patients. The isolated phage was morphologically characterized, and its genome was fully sequenced and annotated, suggesting an ideal therapeutic phage with no lysogenic potential and no observable resistance-transducing abilities.

## 2 Materials and methods

### 2.1 Bacterial isolation, identification and growth conditions

Thirty-two isolates of *K. pneumoniae* were subcultured from biobanked clinical isolates, routinely collected from patients in tertiary care hospitals in Cairo, Egypt, between 2017 and 2019. The isolates were collected from sputum (n = 20) and endotracheal aspirate (n = 12).

The biobanked isolates had been identified by the Vitek 2 automated system (bioMérieux, Marcy l’Étoile, France). Before further subcultures, the bacterial identity of each isolate was verified and confirmed by Gram-staining, followed by culture on differential selective media (MacConkey agar, Neogen, UK) and typical biochemical reactions for *Enterobacteriaceae* (Farmer et al., 1985).

The identity of the bacterial strain used for phage propagation was further confirmed by the polymerase chain reaction (PCR) as previously described (Turton et al., 2010), followed by sequencing of its 16S–23S rRNA internal transcribed spacer. Conserved, predesigned primers (Woese and Fox, 1977) for that region (FP: 5’-ATTTGAAGAGGTTGCAAACGAT-3’ and RP: 5’-TTCACTCTGAAGTTTTCTTGTGTTC-3’) were synthesized by LGC Biosearch Technologies, UK. After PCR, the amplicon size was confirmed by gel electrophoresis, and the DNA was extracted from the 1% (w/v) agarose gel, and sequenced at Macrogen, Korea. The obtained sequence was checked by BLASTn against the rRNA/ITS database at the National Center for Biotechnology Information (NCBI) portal.

For phage isolation purposes, bacterial strains were cultured on tryptic soy broth (TSB, Oxoid, UK) media at 37°C in a shaking incubator at 150 rpm. Also, TSB supplemented with 20% glycerol was used as a maintenance medium to preserve all isolates at -80°C.

### 2.2 Antimicrobial susceptibility assay

The antimicrobial susceptibility of the bacterial isolates was estimated according to the Clinical and Laboratory Standards Institute 2019 recommendations (CLSI) (CLSI, 2019). Colonies of overnight culture were aseptically suspended in sterile isotonic saline solution to be visually comparable to a 0.5 McFarland standard. The bacterial suspensions were swabbed aseptically and uniformly on Mueller-Hinton agar (MHA) (Oxoid, UK). The antimicrobial discs were aseptically added with an antimicrobial susceptibility disc dispenser (Oxoid, UK). The cultures were incubated at 37°C for 18 h. The diameters of the inhibited zone around the discs were measured in triplicates, and the mean diameters were calculated and interpreted in reference to the breakpoints of CLSI 2019 (CLSI, 2019) into either susceptible, intermediate or resistant. The 18 tested antimicrobials (belonging to 10 different antimicrobial classes) were piperacillin, piperacillin-tazobactam, cefuroxime, cefepime, doripenem, meropenem, ertapenem, imipenem, aztreonam, azithromycin, tobramycin, kanamycin, gentamicin, amikacin, ciprofloxacin, levofloxacin, trimethoprim-sulfamethoxazole, and doxycycline. The antimicrobial discs were purchased from Oxoid, UK (**Table S1**).

The results of the antimicrobial susceptibility assay of all isolates were further analyzed to calculate their multiple antibiotic resistance (MAR) index (Krumperman, 1983). In addition, we developed a modified MAR index that considers intermediately susceptible isolates. The modified MAR index is the ratio of antimicrobial agents to which a bacterial isolate is not susceptible (intermediately susceptible or resistant) to the total number of assayed antimicrobial agents. While the MAR index assigns a score of 1 to any antibiotic to which the bacterial isolate is resistant and zero to other cases, the modified MAR index scores resistance as 1 and intermediate susceptibility as 0.5. As Kahlmeter et al. (2019) recommended, resistant and intermediate isolates were considered ‘not susceptible,’ a term they suggested instead of ‘nonsusceptible’ (Kahlmeter et al., 2019). *Not susceptible* bacterial isolates with one or more antimicrobial agents in three or more classes were considered MDR (Magiorakos et al., 2012).

### 2.3 Identification of virulence genes

The bacterial isolates used to determine the phage host range were screened for virulence genes by PCR. These genes were classified into siderophores (*entB*, *iutA*, and *irp2*), types 1 and 3 fimbriae (*fimH*, *mrkD*), located in either the core or accessory genome.

The sequences of the targeted virulence genes were retrieved from the NCBI database. Primers for the virulence genes were designed by Primer-BLAST (Ye et al., 2012) and analyzed by OligoAnalyzer (URL://https://eu.idtdna.com/pages/tools/oligoanalyzer). The primers were tested by the in silico PCR amplification tools (Bikandi et al., 2004), and BLASTn checked the obtained sequence of PCR products. The primers were synthesized by Willowfort, UK (**Table S2**).

For virulence gene amplification, bacterial colonies were boiled in sterile saline solution, and a colony PCR approach was used (Gussow and Clackson, 1989; Azevedo et al., 2017). The PCR products were separated by agarose gel electrophoresis (Sigma Aldrich, Germany), and 100 bp DNA Ladder RTU (GeneDireX, India) was used.

### 2.4 Phage isolation

Several liquid sewage samples were collected from urban and medical sources in different Egyptian cities. The crude sewage samples were used without any treatment. Each isolate of *K. pneumoniae* was cultured overnight in TSB and incubated at 37°C with 150 rpm shaking. A mixed culture of all *K. pneumoniae* isolates was prepared by transferring 0.5 ml of each culture into a 100 ml conical flask with fresh 50 ml TSB. The mixed culture was incubated overnight at 37°C and 150 rpm.

For enrichment, each collected sewage sample was mixed with an equal volume of the mixed culture of *K. pneumoniae* isolates in a sterile 15 ml centrifuge tube (Carlson, 2005; Twest and Kropinski, 2009). Each enriched culture was incubated for 4 h at 37°C in a static incubator. Next, the culture was centrifuged at 2,300 × *g* for 20 min, and 1 ml of the supernatant (phage lysate) was aseptically collected and stored at 4°C. For culturable phage screening, 10 μl of each lysate was spotted twice on tryptic soy agar (TSA, DifcoTM, BD, Franklin Lakes, NJ, USA) plates supplemented with 1% (w/v) top soft TSA, which was inoculated with 100 µl of bacterial broth cultured in its exponential growth phase. The plates were incubated overnight at 37°C. On the following day, the formed phage plaques on the bacterial lawns were excised and suspended in 500 µl sodium magnesium (SM) buffer [5.8 g of NaCl; Fisher Chemicals: S/3120/60, 2 g of MgSO_4_.7H_2_O; Fisher Chemicals: M/1050/60, 5 ml of 2% (w/v) Gelatin; Sigma: G-1393 and 50 ml of 1 M Tris-HCl; Sigma: T-2663 at pH 7.5, in a liter of distilled water (dH_2_O)]. The buffer was initially sterilized in an autoclave and by filtration through a 0.22 μm cellulose acetate (CA) syringe filter (Standard Membrane Filtration Limited, China), and then was left for 2 h at 4°C for phage particle elution. The SM buffer for each plaque was aseptically transferred into new microcentrifuge tubes, and then centrifuged at 2,300 × *g* and 4°C for 20 min. The supernatant was transferred into a new microcentrifuge tube and stored at 4°C. Eventually, the isolated phages were further spotted on their bacterial *K. pneumonai*e hosts for confirmation.

### 2.5 Plaque purification, phage propagation and concentration

The isolated bacteriophages were serially diluted 10 times in SM buffer, then spotted on the surface of dried bacterial lawns and incubated aerobically at 37°C following the spot test (Carlson, 2005). The spotted plates were examined for bacteriophage plaques after 24 h. Single plaques were picked from the top agar layer using a sterile micropipette tip. For phage particle elution, the excised plaques were suspended in a 300 μl SM buffer, vortexed for 10 sec, then left for ≥ 24 h at 4°C. Each phage lysate was centrifuged at 5,500 × *g* for 20 min, and the supernatant was collected aseptically into new sterile microcentrifuge tubes, which were kept at 4°C. This purification procedure was repeated six times for each isolated bacteriophage to produce purified phage stocks. Subsequently, aliquots of purified bacteriophages were used for propagation.

The purified bacteriophages were propagated by the plate lysate method, according to Bonilla et al. (2016), with some modifications. Ten microliters of the purified lysate and 100 µl of a fresh culture of *K. pneumoniae* host strain were mixed with 5 ml melted 1% (w/v) soft TSA, which were poured on a plate containing TSA base agar. Thirty plates were prepared as above, incubated overnight at 37°C, and plaques were aseptically scraped in 3 ml of sterile SM buffer, and left at room temperature for 15 min. The buffer was collected from all plates in one tube and centrifuged at 5,500 × *g* at 4°C for 20 min. The supernatant of phage lysate was transferred to a new sterile tube. Chloroform (1% v/v) was added to the phage lysate and left for 2 min at 4°C; then, the upper layer was extracted into a new sterile tube. The phage lysate was further centrifuged at 5,500 × *g* for 20 min, and the collected supernatant was filtered through 0.22 μm CA syringe filters. The phage preparation was concentrated by centrifugation at maximum speed (18,000 × *g*) for 90 min at 4°C, followed by pellet resuspension in 3 ml SM buffer (modified from Carlson, 2005). Plaques corresponding to the concentrated phage lysates were enumerated by spot test (Carlson, 2005).

### 2.6 Host range determination

**Figure 1:**
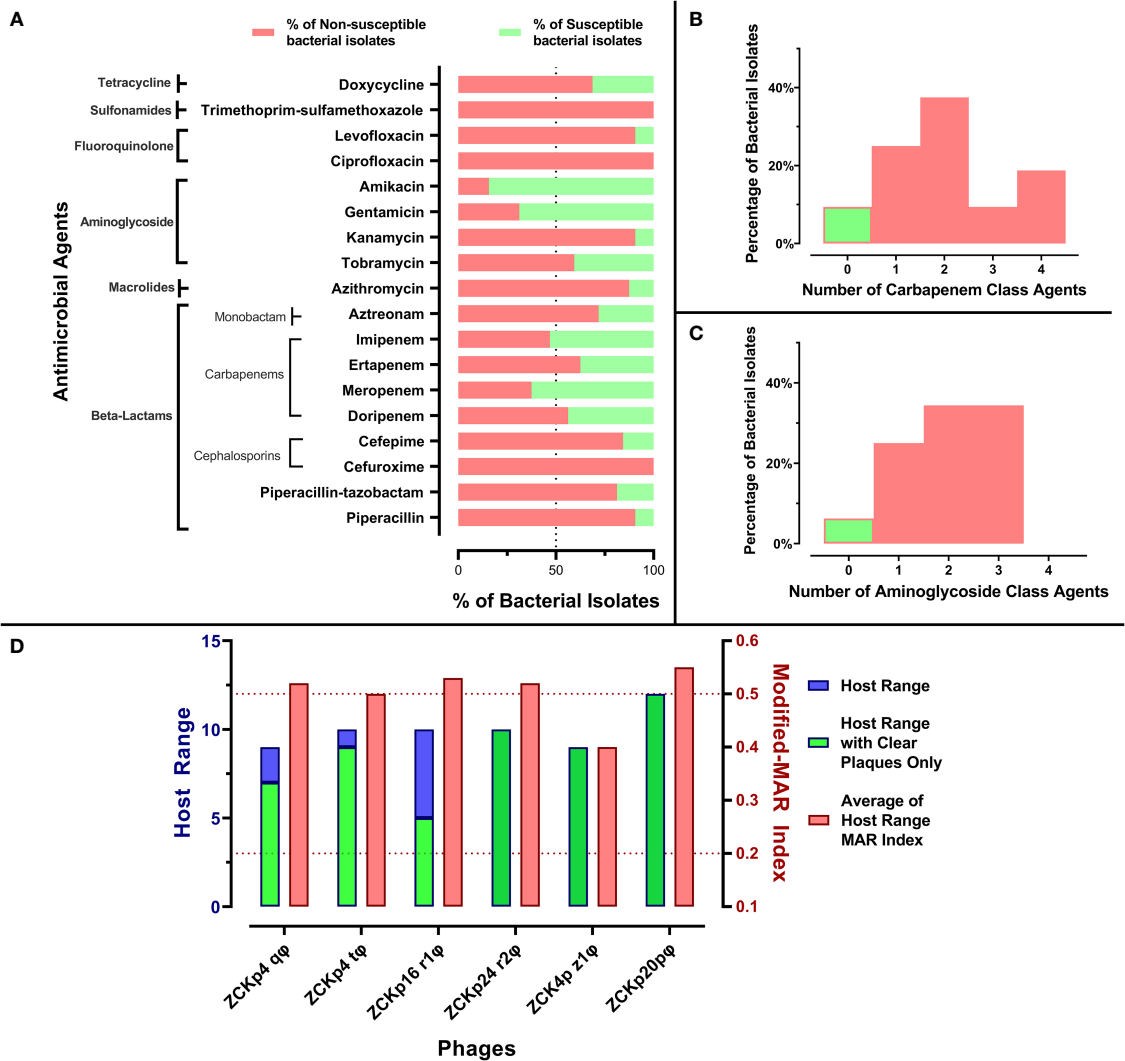
Antibiogram of bacterial isolates and phage host range. **(A)** A stacked-bar chart of bacterial isolates’ resistance and susceptibility percentages to each tested antimicrobial agent. The susceptibility test was carried out by the disk diffusion method, and the results were interpreted according to CLSI 2019. **(B)** A histogram representing the percentage of bacterial isolates not susceptible to one or more carbapenem antibiotic. **(C)** A histogram representing the distribution of bacterial isolates not susceptible to one or more aminoglycoside. **(D)** Host ranges of the six isolated phages and average modified-MAR indices of their bacterial hosts. Blue bars represent the number of bacterial isolates in the host range of each isolated phage, while the green bars specify only the number of hosts that showed clear plaques. The left Y-axis describes blue and green bars. Red bars show the average modified-MAR indices of all isolates included in each isolated phage host range. The scale for red bars is plotted on the right Y-axis.

To determine the phage host range, we used the previously described spot testing assay (Kutter, 2009) on 32 bacterial isolates of *K. pneumoniae* (**Figure 1**). The purified phage lysates were spotted twice on bacterial lawns seeded in TSA plates (TSB with 1% top agar) with 100 μl of each test strain. After overnight incubation, the plates were checked, and their plaques were scored according to their clarity (Kutter, 2009). A clear plaque was scored +3, indicating complete lysis, while an opaque plaque with a hazy or turbid background was scored +2. Spots with non-confluent plaques were scored +1, and spots that showed no plaques were scored 0. For confirmation, the spot testing assay was repeated for bacterial hosts with plaques of scores > +2.

### 2.7 Relative efficiency of plating

Phage effectiveness against *K. pneumoniae* host strains was further assessed by the efficiency-of-plating method (EOP) as previously described (Kutter, 2009). Briefly, a stock of the phage was serially diluted 10-fold (10^1^ to 10^8^). Ten microliters of each dilution were spotted in triplicates on a fresh lawn of each susceptible bacterial isolate identified according to the host range assay. After overnight incubation, the plaques of the same phage titer were enumerated, and the average plaque-forming unit (PFU) count for spot triplicate was calculated for each bacterial isolate. The relative EOP was calculated as the ratio of the average PFU count of the same phage dilution on each bacterial host to the maximum PFU count observed (Kutter, 2009). The EOP was classified into four levels of efficiency from ‘high production’ to ‘no production’ (**Table S3**) (Mirzaei and Nilsson, 2015).

### 2.8 Transmission electron microscopy of bacteriophage morphology

Purified high-titer lysate of 10^10^ PFU/ml of the phage was prepared and sent to the TEM core facility at the National Research Center (NRC), Cairo, Egypt. The prepared lysate was added to a copper grid, fixed with 2.5% (v/v) glutaraldehyde and negatively stained by 2% (w/v) phosphotungstic acid (pH 7.2). Micrographs were obtained by a JEOL 1230 transmission electron microscope (NRC, Cairo, Egypt), and the dimensions of the virion were measured by ImageJ version 1.53n with reference to the scale bar of 100 nm that was generated from the microscope.

### 2.9 *In vitro* evaluation of bacteriolytic activity and burst size

The bacteriolytic activity of the phage was evaluated *in vitro* against its isolating bacterial host at different multiplicity of infection (MOI) values (Carlson, 2005). The phage lysate concentrations 10^6^, 10^7^, 10^8^, and 10^9^ PFU/ml were prepared to achieve MOIs 0.1, 1, 10, and 100, respectively. The challenged bacteria were freshly grown in TSB medium to mid-log phase with a concentration of 10^6^ - 10^7^ colony-forming units (CFU)/ml. The concentration of bacterial suspension was determined according to its optical density at 600 nm. For each MOI, 20 ml of mid-log bacterial suspension was prepared, then divided into tubes labeled as T and C (for test and control, respectively), each containing 10 ml. The added volume of the phage lysate was calculated according to each MOI.

The two tubes were incubated at 37°C with shaking at 100 rpm. The bacteriolytic activity of the phage was evaluated through 11 time points (0, 10, 20, 30, 45, 60, 75, 90, 120, 150, and 210) over 3.5 h of incubation. At each time point, a sample of 100 µl was drawn from both tubes (T and C). The drawn samples were serially diluted 10-fold from 10^-1^ to 10^-10^ in TSB, and each dilution was spotted on agar plates. The titer of the uninfected bacteria was determined as CFU/ml from plates of tube C (Fayez et al., 2021). Additionally, the titer of surviving bacteria after infection and the titer of free phage as PFU/ml were determined from plates corresponding to tube T. The bacterial titers were assayed by the plate count method (Lee, 2008), while the phage titers were determined by spot test (Carlson, 2005). The titers of surviving bacteria in the assayed MOIs were determined to evaluate the most efficient bacteriolytic MOI (Liu et al., 2020).

Moreover, a portion of phage-treated bacterial suspension of MOI 0.1 was collected at every time point. The collected samples were divided into two tubes, one untreated with chloroform for infective centers quantification and the other was treated with 1% (v/v) chloroform to induce lysis and burst of the intact bacterial cells to free intracellular phages. Next, the samples were diluted and spotted on bacterial lawns for post-burst phage titer determination. The burst size was calculated as the ratio of the titer of released virions at plateau to the initial virions titer (Carlson, 2005). These assays were conducted in triplicates.

### 2.10 Phage stability

The phage stability was assessed under various conditions of temperature and pH. Different temperatures were used to cover both storage and different thermal conditions (- 20°C, 4°C, 40°C, 60°C, 70°C, and 80°C). Phage aliquots were adjusted at 10^10^ PFU/ml as starting titers for each temperature. The phage titer of each aliquot were determined at indicated time points for 2 h (10, 20, 30, 60, and 120 min) and spotted after 24 h for an extended time point.

Moreover, the phage stability was monitored over a broad pH spectrum ranging from 2 to 13. The SM buffer pH values were adjusted by either 1M hydrogen chloride (HCl) or 1M sodium hydroxide (NaOH). The phage titers for each pH condition were determined after 1 hour and 24 h of incubation at 4°C. All phage titers were determined by spot test (Carlson, 2005). The temperature and pH stability experiments were conducted in triplicates.

### 2.11 Bacteriophage potency against bacterial biofilm

The anti-biofilm activity of the phage was evaluated at different MOIs (100, 10, 1, 0.1, 0.01, 0.001, and 0.0001) for two different phenotypes: (i) inhibition of biofilm formation and (ii) clearance of formed biofilm. The biofilm formation, staining, and measurement were conducted by the microtiter plate biofilm assay (Merritt et al., 2011) with minor modifications.

A fresh TSB-diluted bacterial culture was grown to a concentration of 10^5^ CFU/ml (exponential phase), estimated by optical density at 600 nm. A volume of 125 μl of the culture was added to wells of flat-bottomed polystyrene microtiter plates (Greiner Bio-One, Portugal). In each microtiter plate, a row of 6 wells containing untreated culture was used as a negative control.

#### 2.11.1 Biofilm inhibition assay

Bacterial cultures were exposed to phage infection from the beginning of the experiment and were incubated at 37°C for 48 h without shaking before crystal violet staining. Each MOI was evaluated in 6 replicate wells. The phage antibiofilm activity was assayed at MOIs (0.0001 to 100) corresponded to concentrations ranged between 10^2^ PFU/ml to 10^8^ PFU/ml. A volume of 10 μl phage of concertation equivalent to the required MOI was added to each well.

#### 2.11.2 Biofilm clearance assay

The same procedure of the inhibition assay was applied to the biofilm clearance except for incubating all bacterial cultures without phage treatment in the wells for 48 h to form mature biofilm. After incubation, the phage preparations were added at different MOIs to the untreated wells of the plate and incubated at 37°C for 24 h.

The optical density at 600 nm (OD600) values of well contents was measured before discarding the media and planktonic cells for normalization, to minimize the variability due to growth rate and cell density. Planktonic cells and media were discarded, and washed in a tap water tank. Plates were left to dump out and dry, then the adhered biofilm was stained by 150 μl of freshly prepared 0.1% (w/v) crystal violet. Excess crystal violet was discarded, and plates were rewashed and left to dry at ambient temperature. The stained biofilms were solubilized in 150 μl of 30% (v/v) acetic acid. The optical density was estimated from the absorbance of solubilize stained biofilm at 590 nm by the FLUOstar Omega Microplate reader (BMG LABTECH, Germany).

### 2.12 Effect on viability of human fibroblasts using MTT assay

The 3-(4,5-Dimethylthiazol-2-yl)-2,5-diphenyltetrazolium bromide (MTT) assay was used to evaluate the effect of phage vB_Kpn_ZCKp20p on the viability of normal human skin fibroblast (HSF) cells. The HSF cells (passage 12) were grown in high glucose Dulbecco’s Modified Eagle’s Medium (DMEM, Biowest, France) supplemented with 10% fetal bovine serum, 100 units/mL penicillin, and 100 mg/mL streptomycin. The cells were seeded with density 8 x 10^3^ cells/well in 96-well cell culture plates (CELLSTAR, Greiner Bio-One, Portugal) and grown in a carbon dioxide incubator at 37°C.

A purified lysate of phage vB_Kpn_ZCKp20p in SM buffer was 10-fold serially diluted in DMEM media to be assayed at different concentrations (10^9^,10^8^,10^7^, and 10^6^ PFU/ml) on a proliferative monolayer of fibroblasts in the wells. The phage effect on the cells’ growth was evaluated after 24 h and 48 h. A negative control of untreated cells in DMEM media and a vehicle control of SM buffer diluted in DMEM media with untreated cells were prepared.

After incubation at each time point, the used DMEM media was replaced with fresh 100 µl DMEM containing 10% MTT labeling reagent (final concentration 0.5 mg/ml) and left in the incubator for 4 h. Then, the media was carefully withdrawn, and an equal volume of DMSO was added per well to solubilize the formed crystals of formazan for 20 min. Next, the wells’ absorbance was measured at 570 nm in a FLUOstar Omega Microplate Reader. The OD values were used to calculate the viability of the phage-treated cells as percentage of the untreated cells (negative control) by the following formula (Carmichael et al., 1987; van Meerloo et al., 2011; Rahimzadeh Torabi et al., 2021; Rezk et al., 2022). The cell viability assay was conducted in four technical replicates and three biological replicates for each incubation time point.


$$Cell\;viability=100-\left(\left(1-\;\frac{OD_{phage-treated\;cells}\;-\;OD_{blank}}{OD_{untreated\;cells}\;-\;OD_{blank}}\right)\times 100\right)$$

### 2.13 Bacteriophage DNA extraction

The genomic DNA of the phage was extracted from 10 ml of purified high-titer (10^10^ PFU/ml) phage lysate by the phenol-chloroform–isoamyl alcohol method, as detailed in Sambrook et al. (2001), with some modifications. Initially, the phage capsid was lysed to release the contained DNA by incubation for an hour at 56°C with proteinase K and sodium dodecyl sulfate (SDS) (10% w/v), which were added to a final concentration of 50 ug/ml and 0.5%, respectively. The solution was then thoroughly mixed with an equal volume of phenol: chloroform: isoamyl alcohol (25:24:1) to separate genomic DNA from proteins. The mixture was separated into two phases by centrifugation at 18,000 × *g* for 10 min, an aqueous phase on the top from which DNA was collected and an organic phase containing proteins, which was discarded.

The extracted DNA in the aqueous phase was precipitated overnight at -20°C by 1:10 volume of 3M sodium acetate (pH 5.2) and 2:1 volume of ice-cold isopropanol. The precipitated DNA was pelleted at 18,000 × *g* for 10 min by centrifugation. The supernatant was discarded, and then the pellet was resuspended in 90% ice-cold ethanol and transferred into a new 1.5 ml tube. The extracted DNA pellet was washed twice in 70% ethanol, left to dry, and finally resuspended in 100 μl nuclease-free water. The DNA concentration and quality were measured by FLUOstar Omega Microplate reader (BMG LABTECH, Germany).

### 2.14 Genome sequencing, assembly and annotation

The extracted DNA was sequenced at the core genomics facility of Zewail City of Science and Technology, with the standard Illumina protocols. Briefly, a genomic library of the extracted DNA of the phage was constructed by the Illumina Nextera tagmentation protocol (Illumina, Cambridge, UK). An Illumina v3 sequence cassette was used for sequencing the tagmented library for 600 cycles on an Illumina MiSeq platform.

Sequence reads were quality checked by FastQC (v0.11.9) and *de novo* assembled by Unicycler (v0.4.8) on the PATRIC platform (v3.6.12) (Davis et al., 2020), currently a part of the BV-BRC portal (https://www.bv-brc.org/). In the PATRIC assembly pipeline, the assembled genome was polished by Pilon (v1.23), and the resulted contig was plotted by Bandage (v0.8.1). The assembly statistics were performed by QUAST (v5.0.2) and SAMtools (v1.3).

Next, the single assembled contig was annotated with the Rapid Annotation using Subsystem Technology Toolkit (RASTtk) pipeline (Aziz et al., 2008). The annotation scheme was customized to start with “annotate-proteins-phage” followed by “annotate-proteins-kmer-v2”. tRNAscan-SE (v2.0) was used to identify tRNA genes (Chan and Lowe, 2019).

After RASTtk annotation, a second-round of annotation was performed for confirmation of assigned functions, or for assigning functions to proteins with unassigned functions. Several tools were used for that purpose, including NCBI BLASTp (against the non-redundant protein database), HHPred, InterPro Scan, and UniProt. Accordingly, the annotated proteins were manually edited in the UGENE software, v43.0 (Okonechnikov et al., 2012), and a final GBK file was generated. A genomic map of the phage was generated by Proksee web-based tool (Jason R. Grant et al., 2022) by CGView family tools (Stothard et al., 2019).

### 2.15 Bioinformatics analysis and comparative genomics

The phage lifestyle was predicted by BACPHLIP (Galaxy Version 1.0) (Hockenberry and Wilke, 2021), which predicts lifestyle based on conserved domains of integrases, excisionases, recombinases, transposases, and partitioning proteins (ParA and ParB). Moreover, the phage therapeutic suitability was evaluated by PhageLeads, which analyses the phage genome for temperate genetic markers, AMR, and virulence genes. Other tools were used to further test the feasibility of the phage for therapy, including RGI Resistance Gene Identifier (RGI v5.2.1, CARD v3.2.3 (Alcock et al., 2020), DBETH (Chakraborty et al., 2012), and VRprofile2 (Zankari et al., 2017; Bortolaia et al., 2020; Wang et al., 2022). PhageTerm (Galaxy v1.0.11) was used to predict the genome termini and packaging from sequence reads (Garneau et al., 2017).

The topology of the phage predicted proteome was analyzed for transmembrane domains by DeepTMHMM, which uses a deep learning protein language model-based algorithm (Hallgren et al., 2022). The genomic sequence was scanned by Phage Depolymerase Finder (PhageDPO) (Galaxy Version 0.1.0) prediction tool to find genes with putative depolymerase function. This tool uses two models, the Support Vector Machine (SVM) and the Artificial Neural Network (ANN) models, to analyze the genomic sequence (Duarte, 2021).

The whole genome sequence of the phage was checked for similarity with deposited sequences in the GenBank-NCBI database by BLASTn. The top hits with the highest scores and identity ≥ 90% were compared to the phage genome, and the average nucleotide identity (ANI) based on BLAST+ (ANIb) was also calculated by JSpeciesWS (Richter et al., 2016). The genomes with the highest ANI were compared to the phage genome and visualized by ProgressiveMauve (Darling et al., 2010). Additionally, these phages were compared in terms of the proteome by the Proteome Comparison tool of the PATRIC platform, which performs protein bidirectional sequence-based comparison by BLASTp (Overbeek et al., 2014).

### 2.16 Phylogenetic analysis and taxonomic assignment

Genome-based taxonomy was conducted according to the guidelines by Turner et al. (2021). We applied a proteome-based clustering strategy using the ViPTree server (Nishimura et al., 2017) for family-level classification. The analysis was conducted with the closely related phages and other dsDNA prokaryotic viruses. Phages with highest VipTree tBLASTx scores (*S_G_*) were selected to construct a more detailed rectangular proteomic tree.

For further classification to taxonomic levels below the family level (genus and species), a phylogenetic analysis of genome-genome distance of the isolated phage with BLASTn top hits, phages of highest (*S_G_*) on ViPTree and an outgroup of other morphotypes and families was performed. The genome-genome distance was computed by Virus Classification and Tree Building Online Resource (VICTOR) (Meier-Kolthoff and Göker, 2017) based on Genome-BLAST Distance Phylogeny (GBDP) method (Meier-Kolthoff et al., 2013). As indicated in the VICTOR documentation, minimum evolution trees were inferred from the calculated intergenomic distances, and FASTME was used for branch support computation. The analyses were supported by 100 pseudo-bootstrap replicates. Trees were rooted at the midpoint (Farris, 1972) and visualized by ggtree (Yu, 2020). The OPTSIL program was used to estimate taxon boundaries at different taxonomic levels (Goker et al., 2009) at recommended clustering thresholds (Meier-Kolthoff and Göker, 2017) and an F value (fraction of links required for cluster fusion) of 0.5 (Meier-Kolthoff et al., 2014). Moreover, VIRIDIC (Virus Intergenomic Distance Calculator) was used to identify intergenomic similarities of the isolated phage genome to the closest phage homologs in BLASTn and other *Caudoviricetes* phages that were filtered from the NCBI virus database. The filtered phages from the NCBI were select according to the following criteria, *Caudoviricetes*, complete RefSeq genome, genome length between 40Kb - 60Kb, and their host type is Gammaproteobacteria. The FASTA sequence of the filtered phages were downloaded and used for VIRIDIC analysis. VIRIDIC applies the traditional algorithm to calculate pairwise intergenomic distances/similarities according to ICTV recommendation (Moraru et al., 2020).

Additionally, genomic relationships between the phage and reference phages genomic sequences on NCBI-GenBank were analyzed with the alignment-free method, PhageClouds (Rangel-Pineros et al., 2021), which calculates intergenomic distances with dashing based on a threshold of 0.25.

Pan-genomic analysis was performed by CoreGenes5 to find shared signature genes of the phage and its close relatives (Davis et al., 2022). The amino acid sequence alignments of the highly conserved signature genes, encoding the terminase large subunit, the major capsid protein, and the DNA polymerase enzyme, were used for further confirmation of genus- and family-level phylogeny. The conserved gene-based phylogenetic analysis was conducted for the isolated phage with its close relatives against an outgroup of podoviruses and myoviruses. The amino acid sequences of the selected conserved genes of the selected phages were aligned by ClustalW, and the evolutionary history was inferred by the maximum likelihood method with Le_Gascuel model (Le and Gascuel, 2008) for terminase large subunit, major capsid protein, and JTT matrix-based model (Jones et al., 1992) for DNA polymerase III beta subunit. The analysis was supported with 1000 bootstrap replicates. The evolutionary rate differences among sites were modeled by a discrete gamma distribution. Tree branch lengths were measured in number of substitutions per site, while all gaps and missing data were entirely removed. The trees of the phylogenetic analyses were constructed in MEGA (v11) (Tamura et al., 2021).

### 2.17 Statistical analysis

All statistical analyses were performed by GraphPad Prism 9.1.1 for Windows (GraphPad Software, San Diego, California, USA). Data are presented as means and standard deviation of the mean (± SD). Control and test sets were compared by *t*-test/one-way ANOVA at a significance level of *P*< 0.05.

### 2.18 Nucleotide sequence accession number

The genomic sequence of phage vB_Kpn_ZCKp20p was deposited in the NCBI nucleotide database (GenBank) under accession number OP373729.

## 3 Results

### 3.1 Characterization of clinical bacterial isolates

#### 3.1.1 Phenotypic and genotypic identification

The clinical bacterial isolates were identified as *K. pneumoniae* by Gram staining, culture characteristics on selective media, and biochemical tests. The representative isolate, K20, was selected for further experiments and was identified both by the automated Vitek 2 system, and by amplification and sequencing of the 16S–23S rRNA ITS region. The sequenced DNA was 99.74% identical (with 100% coverage) to the 16S rRNA gene of several strains of *K. pneumoniae*, for example, *K. pneumoniae* strain 2021CK-00608 (GenBank Acc. No. CP104678.1).

#### 3.1.2 Antibiotic resistance profile and multiresistance indices of clinical bacterial isolates

All bacterial isolates were categorized as MDR as they were *not susceptible* to at least one agent in three or more classes. Additionally, the MAR indices of all isolates were above 0.2, and 26 isolates (~80%) had modified-MAR indices > 0.5, reflecting a rather highly resistant profile. Strikingly, three commonly used antibiotics, cefuroxime, ciprofloxacin, and trimethoprim-sulfamethoxazole, were ineffective against all isolates. Susceptibility to piperacillin, kanamycin, levofloxacin, azithromycin, cefepime, piperacillin-tazobactam, and aztreonam was observed in< 30% of the isolates (**Figure 1A**). About 90% of the isolates were not susceptible to at least one member of the carbapenem and aminoglycoside classes, and the majority of the isolates (> 65%) were not susceptible to at least two antibiotics within the carbapenem and aminoglycoside classes (**Figures 1B, C**). In contrast, more than 50% of the bacterial isolates were susceptible to amikacin (84%), gentamicin (69%), meropenem (62.5%), and imipenem (53%) (**Figure 1A**). In conclusion, the antimicrobial profile of the isolates reflected their resistance to critical antimicrobial classes, and their MAR indices suggested that the isolates were collected from sources with high burden of resistant microbes, perhaps owing to excessing use of antibiotics (Davis and Brown, 2016).

### 3.2 Phage isolation and host range determination

Six distinct phages were isolated from urban and medical sewage of three different cities to ensure source diversity. Phage vB_Kpn_ZCKp20p, the focus of this study, was isolated from the medical sewage of a healthcare facility in Cairo.

The host range broadness and antimicrobial profile distinguished the isolated phages as potential therapeutic agents. Out of 32 tested *Klebsiella* isolates, the isolated phages lysed 9 to 12 hosts: Phages ZCKp24r2, ZCK4pz1, and vB_Kpn_ZCKp20p formed clear plaques with all affected hosts, while phages ZCKp4t, ZCKp4q, and ZCKp16r1 formed clear plaques on only 90%, 77%, and 50% of their host lawns, respectively (**Figure 1D**).

**Figure 2:**
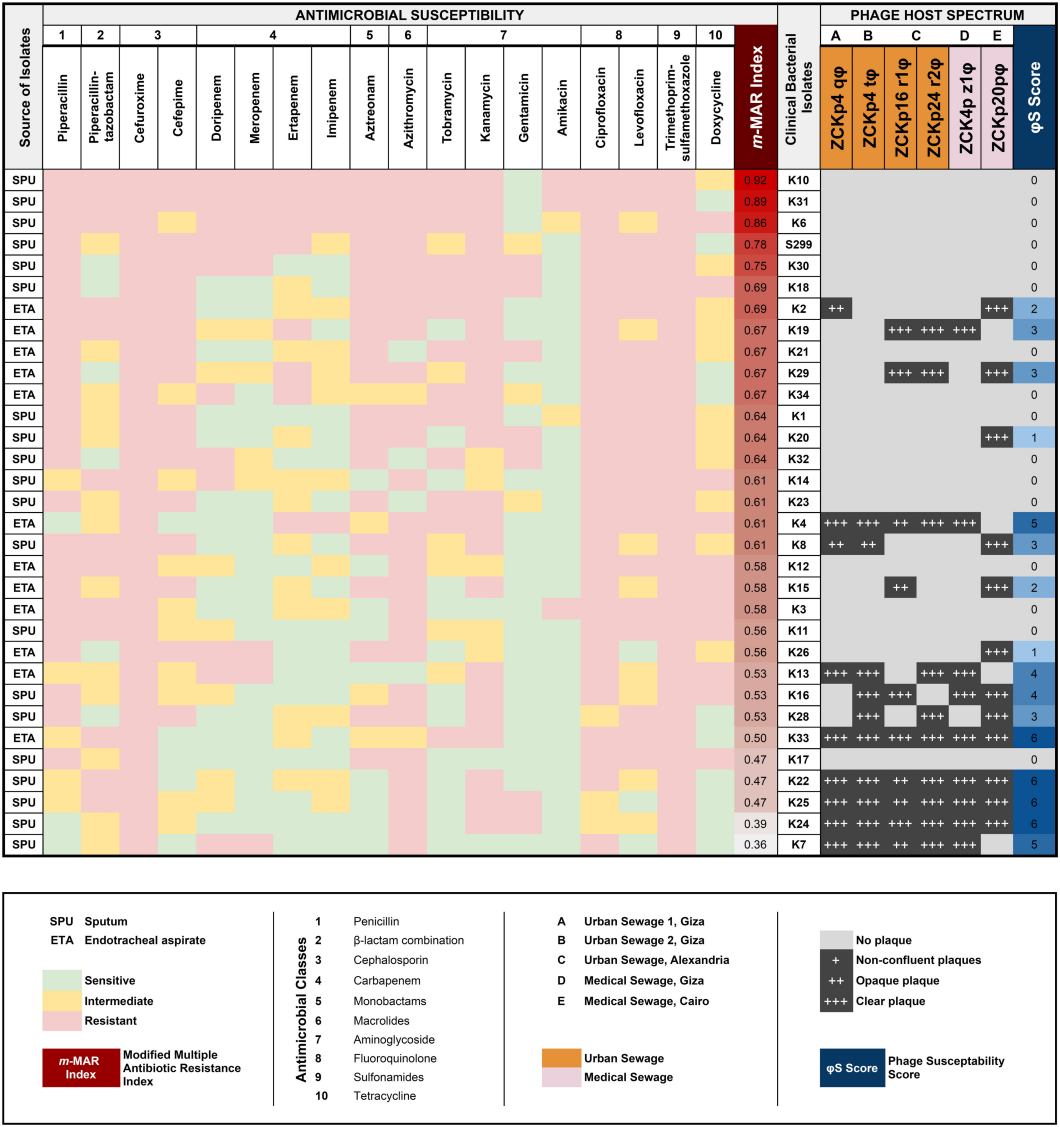
Antimicrobial and phage susceptibility profile of bacterial isolates. A heatmap of the antimicrobial profile of each bacterial isolate against 18 antimicrobial agents of 10 classes, with the estimated modified-MAR index for all isolates. The sources of isolated bacteria (SPU = sputum; ETA = endotracheal aspirate) are indicated in the leftmost column. In a grayscale matrix, the host range of each of the isolated phages is presented according to the plaque clarity (non-confluent plaque [+] to clear plaque [+++]) against the bacterial isolates. The host range of each phage is given a score (represented as a blue shaded gradient) that reflects the number of susceptible host isolates. The isolated phages are color-coded according to their source of isolation. A partial negative correlation is observed between the bacterial resistance of an isolate and its susceptibility to phage infection (Spearman rank correlation coefficient *r_s_* = - 0.6139, *P* = 0.00019).

When bacterial isolates were arranged according to their modified-MAR indices from the highest (0.92) to the lowest (0.36), a negative correlation between the host ranges of the isolated phages and bacterial resistance profile was observed (Spearman’s correlation *r_s_* = -0.6139, *P* = 0.00019, **Figure 2**). No bacterial host with a modified-MAR index above 0.69 was susceptible to any of the isolated phages, and the most susceptible bacterial isolate, to infection by both phages ZCKp4q and vB_Kpn_ZCKp20p, was K2 (modified-MAR index = 0.69). In contrast, the bacterial hosts with modified-MAR indices ≤ 0.5 were susceptible to four to six phages, except for one isolate (K17).

These preliminary results reflected that phage vB_Kpn_ZCKp20p had the broadest host range, of which the bacterial isolates had the highest average of modified-MAR indices; thus, phage vB_Kpn_ZCKp20p was selected for further characterization and genomic analysis.

The pathogenicity of *K. pneumoniae* is associated with genes encoding for virulence factors; these genes are either part of the core or accessory genome. In this study, the susceptible bacterial isolates to phage vB_Kpn_ZCKp20p were screened by PCR for siderophore-related genetic determinants, including enterobactin (*entB*), aerobactin (*iutA*), and yersiniabactin (*irp2)*, in addition to, type 1 fimbriae (*fimH)*. The *entB* and *iutA* genes, associated with enterobactin biosynthesis and aerobactin uptake, respectively, were detected in all tested isolates. Additionally, the yersiniabactin genetic determinant was identified in all isolates, except K22. Lastly, *fimH* and *mrkD* were identified in all bacterial isolates susceptible to phage vB_Kpn_ZCKp20p.

### 3.3 Phenotypic characterization of phage vB_Kpn_ZCKp20p

#### 3.3.1 Morphological characteristics

**Figure 3:**
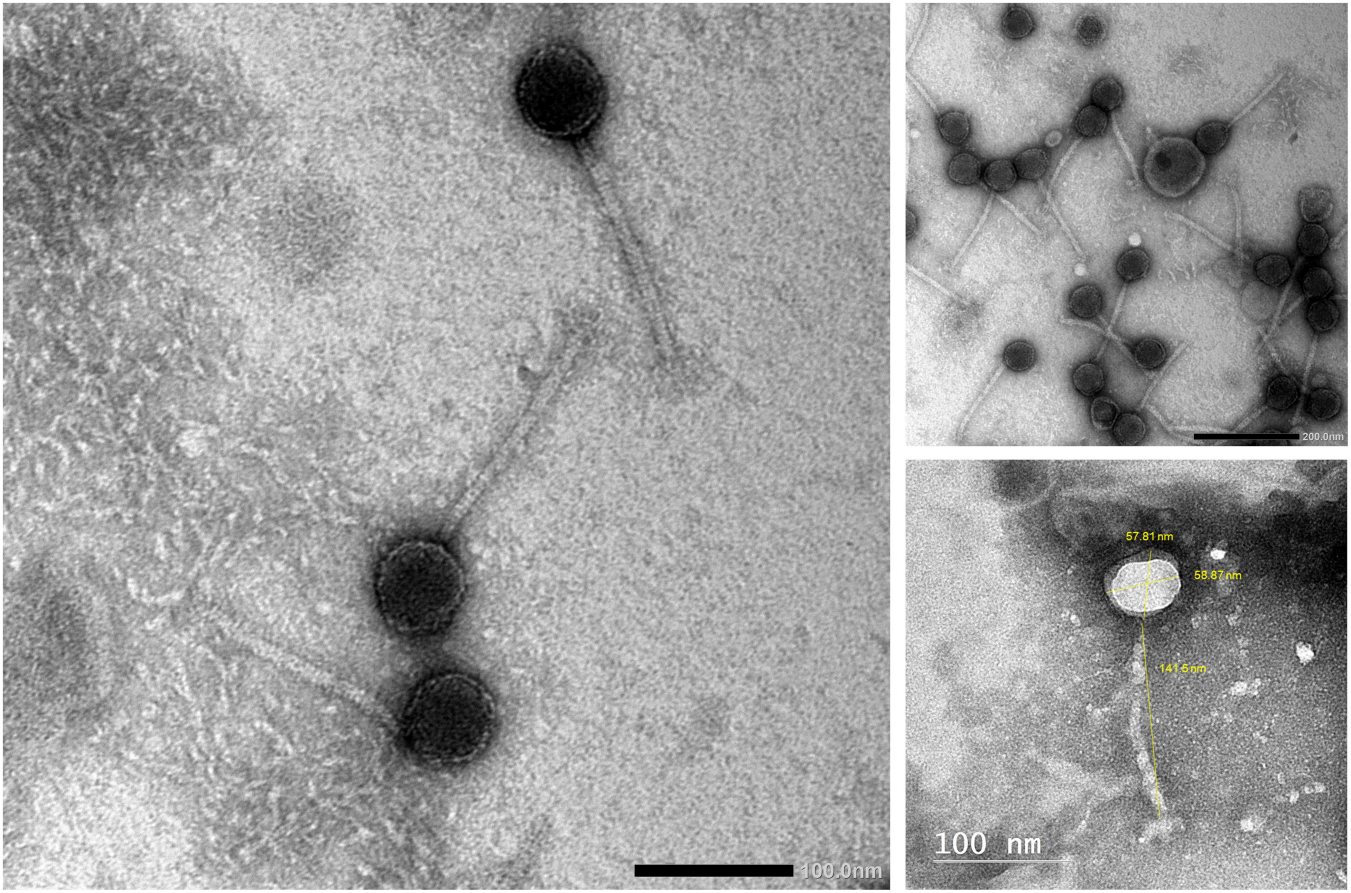
Transmission electron micrograph of phage vB_Kpn_ZCKp20p. Phage particles were negatively stained with 2% (w/v) phosphotungstic acid and obtained by JEOL 1230 at a scale bar of 100 nm. The virion dimensions were measured by ImageJ version 1.53n.

Phage vB_Kpn_ZCKp20p was examined by TEM (**Figure 3**), which revealed the virion structure as an icosahedral capsid of a diameter ~58 nm and a long, noncontractile, flexible tail of dimensions ~141 nm × ~10 nm, which ends with short terminal fibers. These morphological features are consistent with a siphoviral morphotype (legacy family *Siphoviridae*, order *Caudovirales*) according to the ninth report of the International Committee on Taxonomy of Viruses (ICTV) (King et al., 2011).

#### 3.3.2 Relative efficiency of plating

Phage vB_Kpn_ZCKp20p virulence (i.e., lytic potential) was further assessed in terms of relative EOP. Bacterial isolate K15 was considered the indicator host for estimating the relative EOP as it produced the highest phage titer. All phages demonstrated a high EOP (> 0.5) in susceptible isolates, including the isolation host K20, except for two isolates (K16 and K26), in which the phage yielded medium EOP (< 0.5 - > 0.1) (**Figure S1**).

#### 3.3.3 *In vitro* bacteriolytic activity and burst size

**Figure 4:**
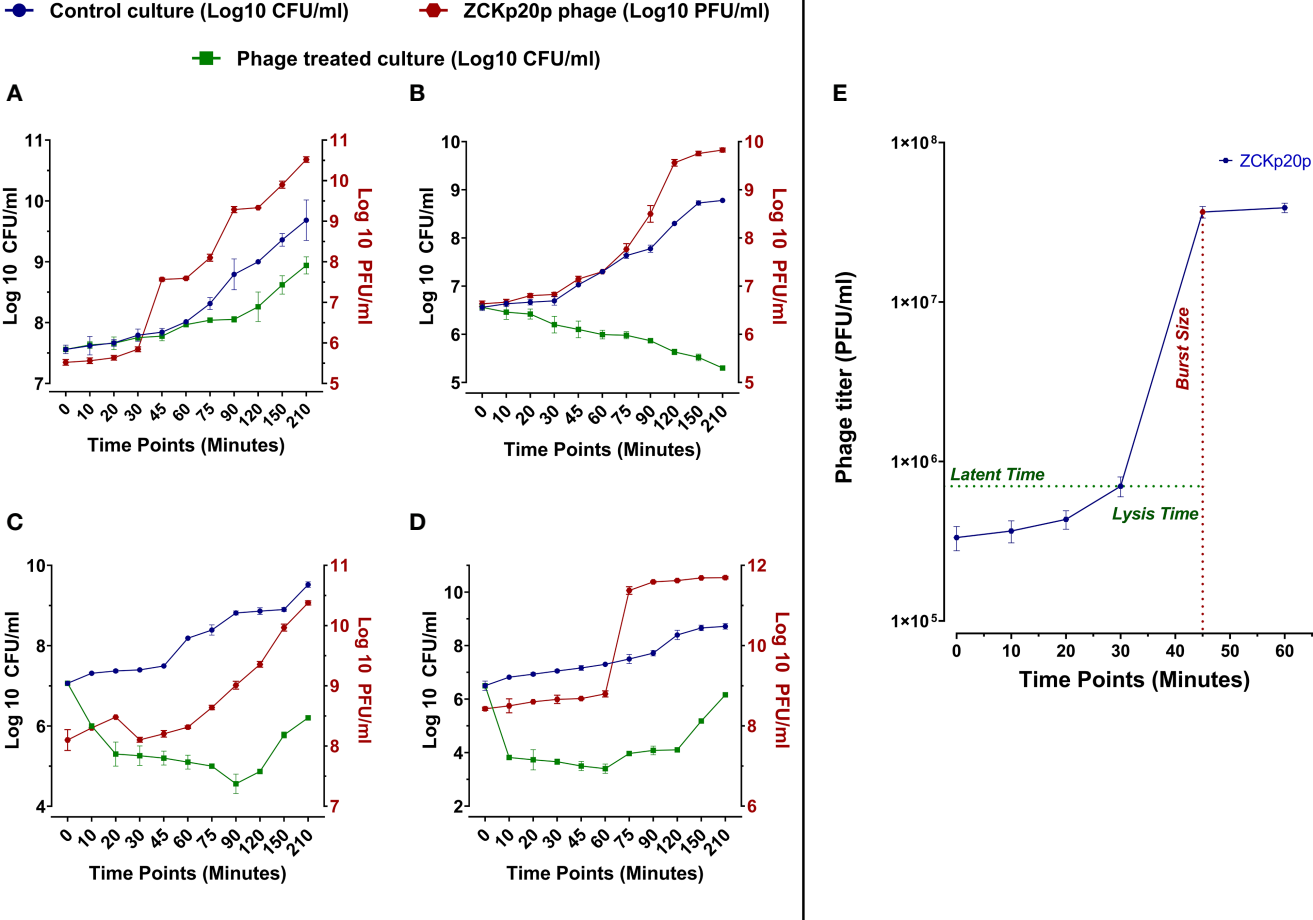
*In vitro* bacteriolytic dynamics of phage vB_Kpn_ZCKp20p at different MOIs (0.1, 1, 10, and 100) and one-step growth curve. MOIs are separately represented in the above charts (**A**–**D**, respectively). Blue and green lines are plotted against the left Y-axis, representing the mean bacterial count of the control culture (untreated) and the survived bacteria of phage treated culture, respectively. The red line represents the mean of phage titers determined by spot test (Carlson, 2005). The mean of phage titers is plotted against the right Y-axis. The results were plotted as the mean of triplicates with error bars of ± SD. **(E)** One-step growth curve of phage vB_Kpn_ZCKp20p at MOI 0.1. Results are mean values ± SD of phage titer (PFU/ml). The phage titer (blue line) was determined at an interval of 10 to 15 min post-infection, and titers were determined by spot test (Carlson, 2005)—the latent period of about 30 min and the lysis time (∼15 min) during which the bacterial cells start to burst till they reach a maximum and form a plateau of released PFU. The burst size (red) was estimated at 100 PFU/cell ( ± 10).

The dynamics of phage vB_Kpn_ZCKp20p’s bacteriolytic activity were evaluated at different MOIs (0.1, 1, 10, and 100) within 210 min. The bacteriolytic activity of the phage was determined by the significant reduction in the growth of the bacterial host in a treated broth culture with the phage compared to the untreated (control) bacterial culture. The dynamics of the phage antibacterial activity varied according to the tested MOI (**Figures 4A-D**). The significant reduction (*P*< 0.05) in bacterial growth was initiated at different time points of 75 and 20 min at each MOI of 0.1 and 1, respectively, while it took only 10 min at MOI 10 and 100. The challenged bacteria did not recover or develop resistance against the phage at MOI 1 throughout the experiment. However, the bacteria were recovered at MOI 0.1/10 and 100 after 150 and 75 min, respectively. After 210 min, the treated bacterial count was significantly reduced by 4.8 × 10^5^ CFU/ml (*P*< 0.05), 5.9 × 10^8^ CFU/ml (*P*< 0.0001), 3.3 × 10^9^ CFU/ml (*P*< 0.0001), and 5.3 × 10^8^ CFU/ml (*P*< 0.0001) at MOIs 0.1, 1, 10, and 100, respectively, in comparison to the control titer. The phage production dramatically increased with high significance (*P*< 0.0001) at MOI 0.1, 1, 10, 100 by 2.49 × 10^11^ PFU/ml, 4.67 × 10^11^ PFU/ml, 8.99 × 10^10^ PFU/ml, and 4.93 × 10^11^ PFU/ml, respectively. Phage titers did not reach an asymptote during the entire experiment time at all MOIs except for MOI 100.

The phage replication was analyzed through a one-step growth curve at low MOI (0.1) under standard growth conditions, which revealed an approximate latent period of 30 min, lysis time of 15 min, followed by a burst size of 100 ( ± 10) PFU per infected host cells (**Figure 4E**).

#### 3.3.4 Temperature and pH stability

**Figure 5:**
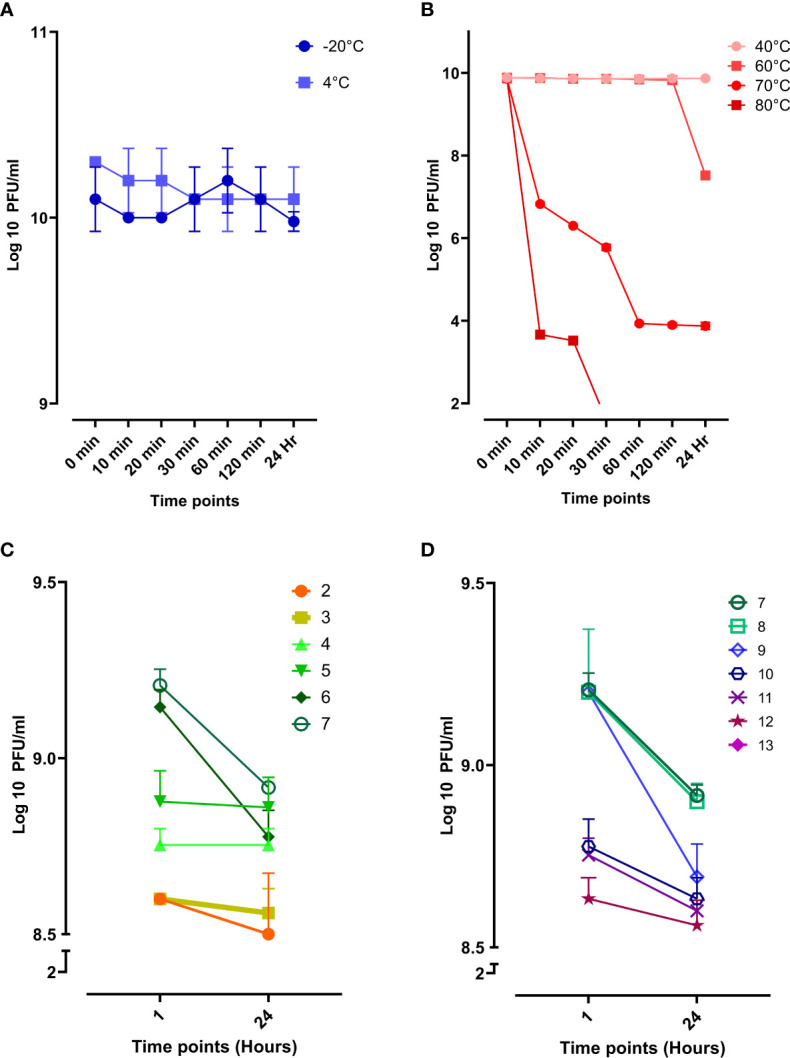
Stability of phage vB_Kpn_ZCKp20p at different temperatures and pH values. **(A)** Storage temperatures of 20°C and 4°C are represented in blue and light blue, respectively. **(B)** Phage stability at different thermal temperatures, represented in a red hue (80°C, dark red to 40°C, light red). **(C)** Phage stability in the acidic spectrum from pH 2 to 7. **(D)** Phage stability in the alkaline spectrum from pH 7 to 13; At pH 13, no survived phages were detected. The results were plotted as the mean of triplicates with error bars of ± SD.

Evaluating the phage stability is vital for therapeutic purposes as the phage might be exposed to harsh conditions during storage, transportation, and downstream processing. Phage vB_Kpn_ZCKp20p was stable at storage temperatures (-20°C and 4°C) without any reduction in the phage titer during the entire assay time (**Figure 5A**). Next, the phage was assayed for thermal stability at temperatures of 40°C, 60°C, 70°C, and 80°C (**Figure 5B**). Phage vB_Kpn_ZCKp20p was highly stable at 40°C till the assay endpoint, and it was also stable at 60°C for 2 h, but the phage titer was reduced by two orders of magnitude after 24 h. The phage tolerated 70°C, but its titer was significantly reduced (*P* < 0.0001) by about 6 log cycles after an hour, and it reached a plateau till 24 h. However, phage vB_Kpn_ZCKp20p did not tolerate 80°C, as a its titer was massively reduced (*P* < 0.0001) within the first 10 min, and no surviving phages were detected from 30 min to the assay endpoint.

Additionally, phage vB_Kpn_ZCKp20p stability was challenged at a wide array of pH values (2 to13), and the survived phages were estimated after an hour and 24 h. The phage titers at different pH conditions were compared to pH 7, neutral pH. Phage vB_Kpn_ZCKp20p survived at all assayed acidic pH levels, and limited reduction (< 8%) was observed in the phage titer after 24 h when compared to the pH 7 titer (**Figure 5C**). At the alkaline pH range, the results were comparable to the acidic pH except for pH 13, where the phage titer completely vanished without any detectable survival after 1 hour of exposure (**Figure 5D**). The phage titer at 24 h of pH 7 and 8 revealed no significant difference (*P* > 0.05). Collectively, both pH (7 and 8) values were considered the most optimum.

### 3.4 Antibiofilm activity of phage vB_Kpn_ZCKp20p

**Figure 6:**
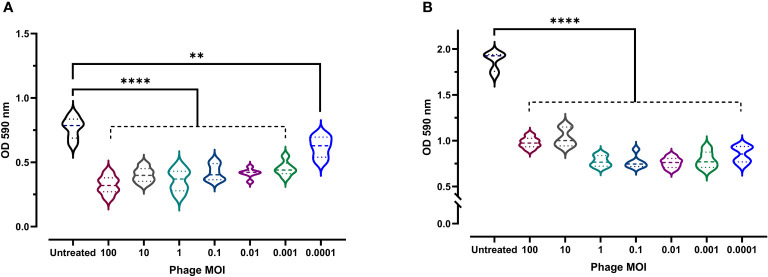
Antibiofilm activity of phage vB_Kpn_ZCKp20p. **(A)** Phage vB_Kpn_ZCKp20p inhibited biofilm formation at different MOIs for 48 h in 96-well plates. **(B)** Phage vB_Kpn_ZCKp20p degraded a 48-hour pre-formed mature biofilm at different MOIs for 24 h in 96-well plates. Statistically significant differences are marked by asterisks, where ** indicates *P* < 0.01 and **** indicates *P* < 0.0001.

Given the clinical importance of biofilm formation (as they boost bacterial resistance and virulence), the antibiofilm activity of phage vB_Kpn_ZCKp20p was experimentally assessed at a wide range of MOIs. Both biofilm formation inhibition and disruption of a pre-formed mature biofilm were evaluated. At all assayed MOIs, bacterial biofilm formation was significantly reduced (*P* < 0.0001, except for MOI 0.0001 as *P* = 0.0039) in comparison to the untreated culture after 48 h of incubation (**Figure 6A**). The highest antibiofilm activity was achieved at MOI 100 (OD: 0.32); however, there was no significant difference (*P* > 0.24) in the results at MOIs ranging from 100 to 0.01. Therefore, MOI 0.01 was considered the lowest MOI with the highest inhibitory effect against biofilm formation.

The capacity of phage vB_Kpn_ZCKp20p to degrade mature biofilm, reflecting its invasion efficiency, was evaluated. A 48-hour-old biofilm was exposed to different phage MOIs, which succeeded in subverting the biofilm after 24 h of all treated cultures, as their ODs were significantly lower (*P*< 0.0001) than the OD of untreated culture with a mature biofilm (**Figure 6B**). The lowest OD (0.75) was observed at MOI 0.01, which was not significantly different (*P* > 0.5) when compared to the results at MOIs 1, 0.1, 0.001 and 0.0001. In summary, 0.01 was the lowest MOI of phage vB_Kpn_ZCKp20p with the highest antibiofilm activity to inhibit biofilm formation and disrupt the mature biofilm.

### 3.5 Effect of vB_Kpn_ZCKp20p on viability of human cells

**Figure 7:**
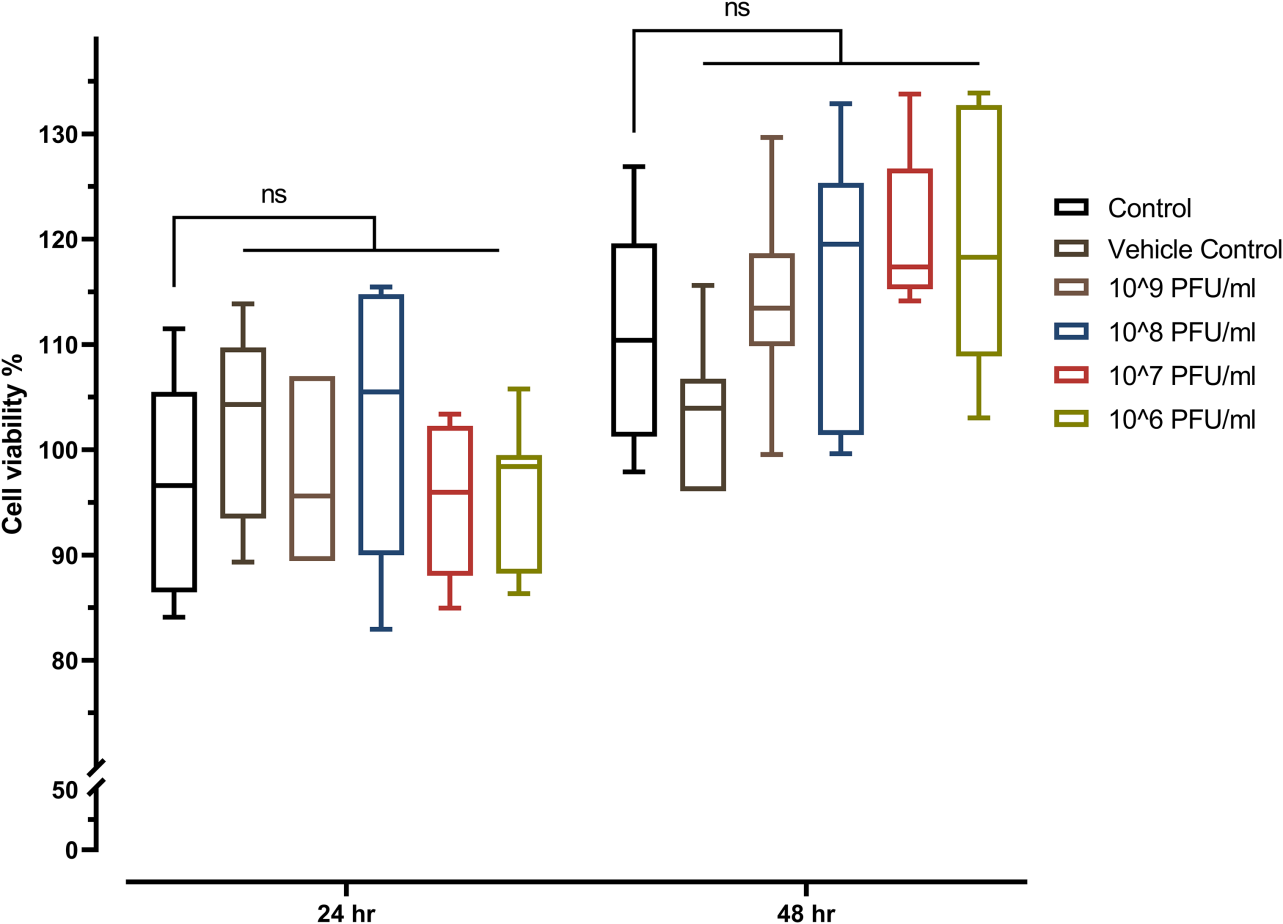
MTT assay of phage-treated HSF cells viability seeded at a density of 8 × 10^3^ cells/well in a 96-well cell culture plate. The mean percentage of viable cells after exposure to four different concentrations of phage vB_Kpn_ZCKp20p for 24 h and 48 h were statistically compared to the mean viability of control cells (untreated cells). The vehicle control was SM buffer (the phage vehicle) diluted in DMEM media corresponding to the prepared phage dilutions. The mark ‘ns’ indicates statistically non-significant differences (*P* > 0.05).

The toxicity of phage vB_Kpn_ZCKp20p was evaluated *in vitro* by the MTT assay on a common type of human cells (HSF). The MTT assay results did not demonstrate any significant reduction in the cells’ viability after they were exposed to the phage for 24 h and 48 h, which ruled out the cytotoxicity of all assayed phage concentrations (10^6^ PFU/ml to 10^9^ PFU/ml) (**Figure 7**).

### 3.6 Genomic characterization and phylogenetic analysis of phage vB_Kpn_ZCKp20p

#### 3.6.1 Genomic analysis and annotation

To further characterize phage vB_Kpn_ZCKp20p and whether it has any genetic components that may interfere with therapeutic applications, we sequenced its genome. After DNA extraction, library preparation, and Illumina sequencing, 18,459 paired-end sequence reads were generated, with an average length of 140 bp, and a total number of bases is 5,203,310.

The reads were assembled into one contig of size 48,797 bp with average read coverage of 69x and GC content of 47.91%. PhageTerm analysis suggested that phage vB_Kpn_ZCKp20p possesses a terminally redundant genome, with partially circular permutations. This pattern corresponds to the headful packaging mode in which linear concatemeric DNA is cut randomly during phage assembly to fill the capsid with the genome regardless of the cut sites; usually, this mode fills the capsid with more than 100% of the genome size.

**Figure 8:**
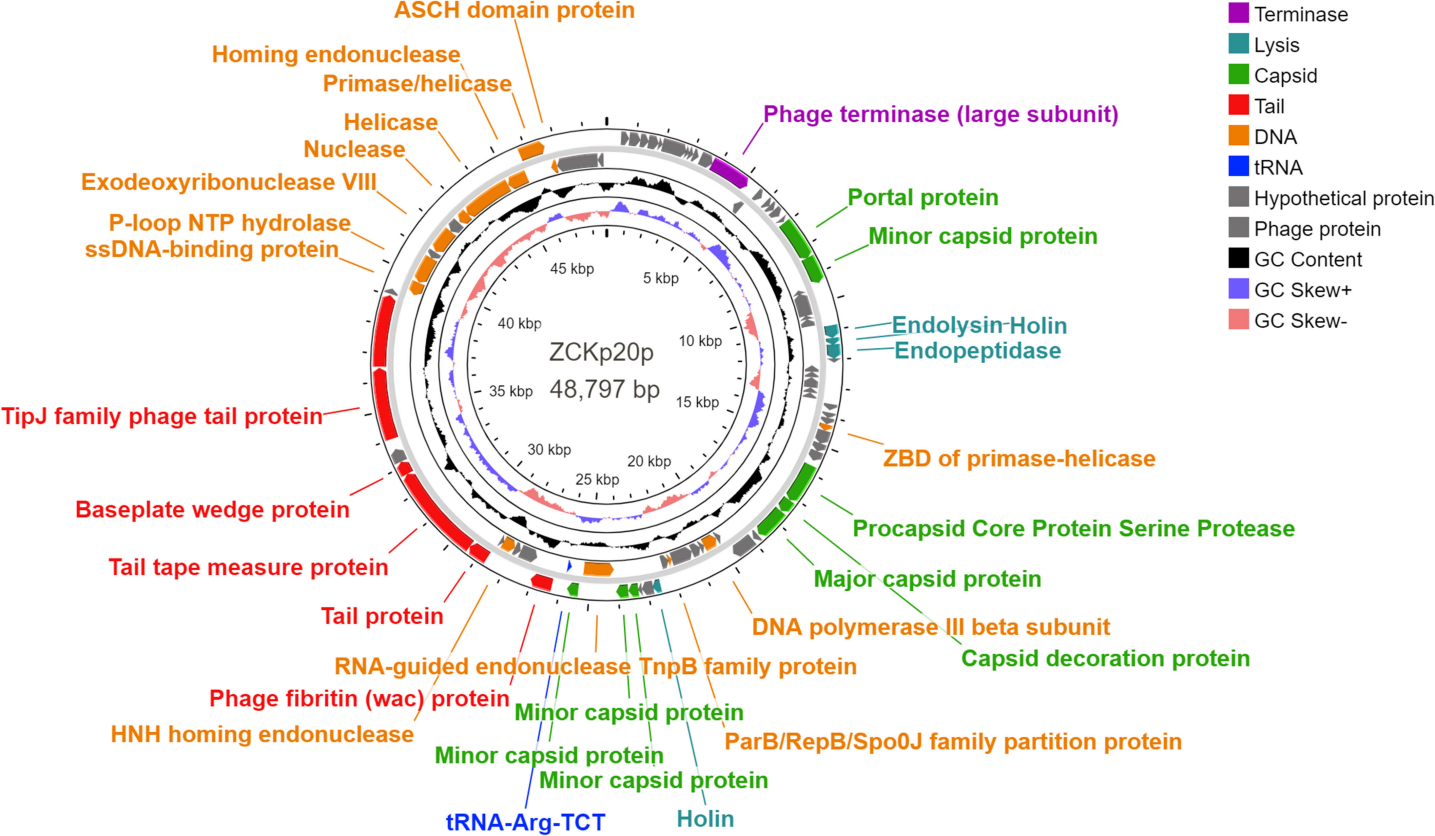
Genomic circular map of phage vB_Kpn_ZCKp20p. Different colors represent the coding sequences (CDS) according to the category of the predicted function: terminase (violet); lytic enzymes (turquoise); capsid proteins (green); tail proteins (red); DNA metabolism, replication and repair-related proteins (orange); tRNA gene (blue); phage and hypothetical proteins (grey). The GC content skew is represented in the middle circle with black color. The inner circle represents the GC skew in light blue and light red colors for above and below averages, respectively.

Eighty-five open reading frames (ORFs) were predicted in the complete genome of phage vB_Kpn_ZCKp20p, of which 33 were assigned known functions, including one gene encoding tRNA-Arg, anticodon TCT (**Figure 8**). For all predicted ORFs, the start codon was ATG, except for one gene encoding a putative nuclease, as it started with TTG. The transcriptional orientation of all predicted genes with putative functions was on one strand, except for the tRNA gene and 11 genes encoding enzymes related to DNA replication. Finally, out of 52 identified ORFs with unknown protein functions, nine matched other phage protein-coding genes, while 43 ORFs were not confirmed to encode phage proteins. The annotated genes with known encoded functions were classified into four groups: DNA replication/repair/transcription (8 genes), virion structure (12 genes: 6 capsid- and 6 tail-associated genes), genome packaging and assembly (8 genes), and four genes related to host cell wall lysis (**Table S4**). DNA-associated functions included helicase, primase, exodeoxyribonuclease VIII, single-stranded-DNA binding protein, and DNA polymerase III.

Structural protein-coding genes are categorized into capsid and tail, found in groups in the same transcription direction. The module of putative capsid genes responsible for the portal, major, and minor capsid-encoding proteins was located between nucleotide positions 6,955 to 25,763, with intervening genes encoding DNA-associated proteins and lytic enzymes. The tail-encoding genes, which included fibritin, tape measure, baseplate wedge, TipJ, and tailspike proteins, were arranged between nucleotide positions 26,297 to 39,020. PhageDPO tool detected depolymerase activity in the tailspike protein (ORF 72) by both SVM and ANN models with 100% prediction (**Table S5**). The genes that contribute to genome packaging and assembly included large terminase (TerL), homing endonuclease, RNA-guided endonuclease (TnpB), procapsid protease, and capsid decorative protein.

Moreover, the genome encompasses genes with putative encoded lysis functions, which included holins, endopeptidase Rz, and endolysin. Adjacent genes of endolysin-holin-endopeptidase were observed starting from base 11,575 to 12,873 with no intergenomic spaces and a few overlapping nucleotides (< 23 bp). The ribosomal binding site (RBS) was located upstream of these genes at base 10833, which followed RBS-start codon consensus. DeepTMHMM predicted transmembrane topology of class II holins in two holins putative proteins (ORFs 27 and 54) as they include two alpha-helical transmembrane domains with 100% probability (**Figure S2**).

These results suggest that phage vB_Kpn_ZCKp20p genome contains all core phage genes. The genome was further checked for the feasibility of a therapeutic application. BACPHLIP predicted a virulent lifestyle for phage vB_Kpn_ZCKp20p (with 91.25% probability); likewise, PhageLeads did not identify any lysogeny, virulence or AMR-related genes. Additionally, using more tools for genomic screening (RGI v5.2.1, ResFinder (v4.1), DBETH, and VRprofile2) could not detect any virulence or AMR-related genes. Taken together, all performed genomic analyses confidently suggest the safety and the potential of phage vB_Kpn_ZCKp20p for therapeutic application.

#### 3.6.2 Comparative genomic and proteomic analyses

To explore the phylogenetic neighborhood of phage vB_Kpn_ZCKp20p, we screened public NCBI nucleotide data (GenBank) using BLASTn and identified 11 phages with high similarity (> 85% sequence identity over 46% - 88% coverage, **Table S6**). The 11 phages have been classified under the former family *Siphoviridae*, and only two phages (*Vibrio* phage pYD38-A and *Aeromonas* phage pIS4-A) were assigned to a genus (*Roufvirus*).

The global alignment of phage vB_Kpn_ZCKp20p to its 11 phylogenetic neighbors resulted in ANIb values > 84.5%. Two of the 11 aligned phages (*Klebsiella* phage ZCKP8 and *Klebsiella* phage 6991) had ANIb% above the 95% cut-off, suggesting they belong to the same species of vB_Kpn_ZCKp20p (**Table S7**). These phages were compared to phage vB_Kpn_ZCKp20p at the genomic level by ProgresiveMauve and at the predicted proteome level with PATRIC Proteome Comparison tools.

**Figure 9:**
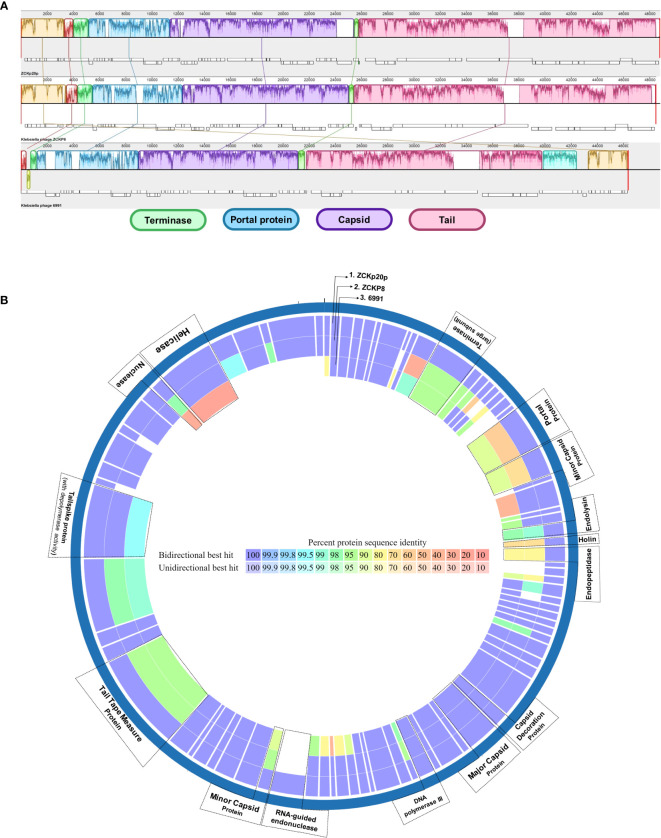
Comparative genomic map of phage vB_Kpn_ZCKp20p and closely related phages. **(A)** Progressive MAUVE alignment of phage vB_Kpn_ZCKp20p and closely related phages *Klebsiella* phages ZCKP8 and 6991: genomic regions with homology are represented in the same colors. **(B)** Comparative circular map of protein-coding genes of phage vB_Kpn_ZCKp20p and closely related phages (*Klebsiella* phages ZCKP8 and 6991) on PATRIC.

The genomic comparison indicated high similarity between the three phages in genomic regions of terminase, portal, capsid-related, and tail-related putative genes (**Figure 9A**). Moreover, the proteome comparison suggested orthology of several protein-coding genes (based on identity > 95% at the amino acid sequence level). These orthologs are: terminase (ORF 12), endolysin (ORF 26), capsid decoration (ORF 43), major capsid (ORF 44), DNA polymerase III (ORF 48), tail tape measure (ORF 68), and tail spike proteins (ORF 72) (**Figure 9B**). The portal protein of phage vB_Kpn_ZCKp20p was more similar to that of *Klebsiella* phage 6991 than of *Klebsiella* phage ZCKP8. Holin (ORF 27) and endopeptidase (ORF 28) proteins of the compared phages to that of phage vB_Kpn_ZCKp20p were identical by 77-74% and 86-90%, respectively, with sequence coverage ∼of 99% (**Table S8**).

#### 3.6.3 Phylogenetic analysis

**Figure 10:**
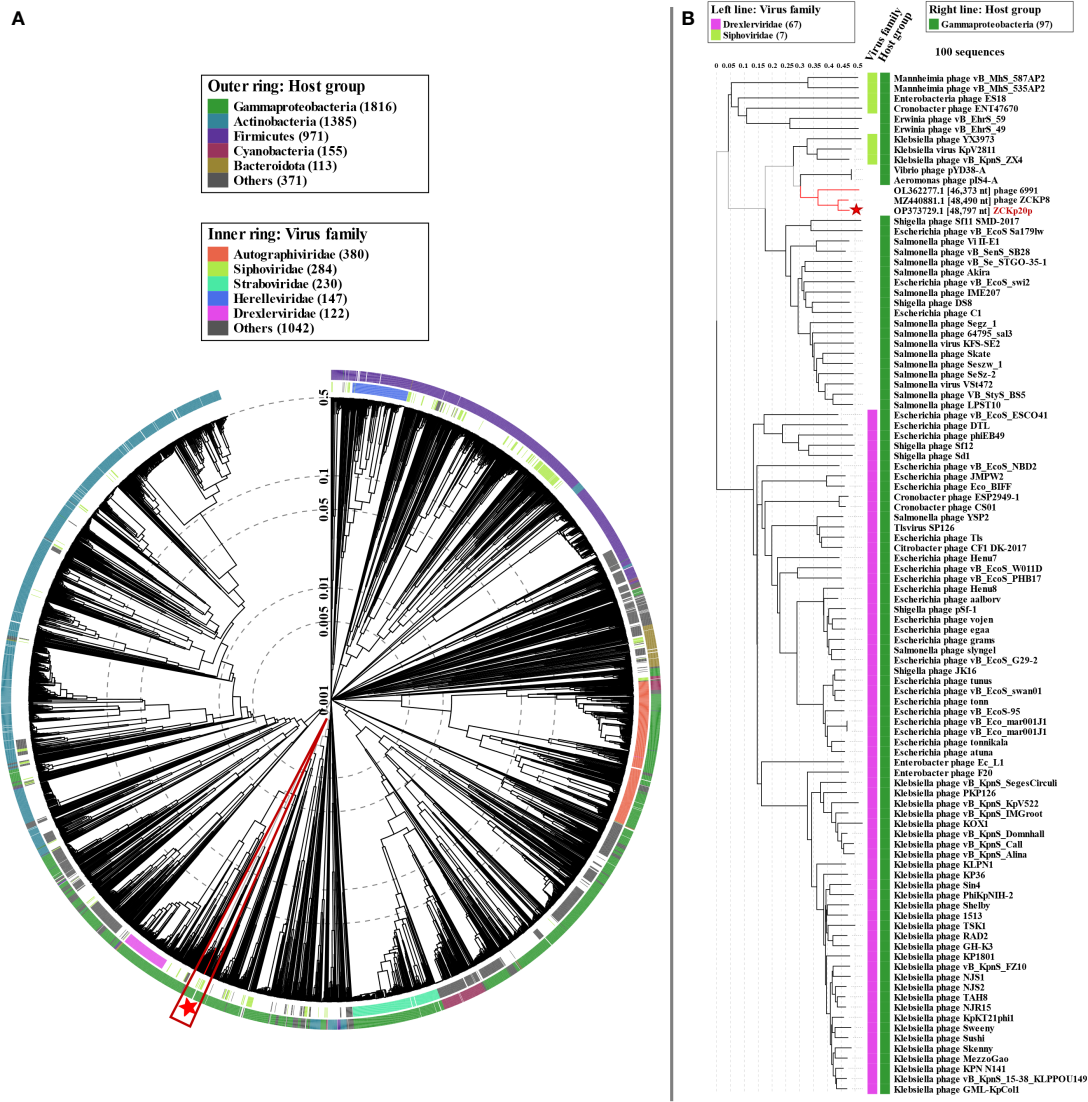
Proteomic tree of phage vB_Kpn_ZCKp20p. **(A)** Circular proteomic tree based on genome-wide similarities of phage vB_Kpn_ZCKp20p (marked with a red star), top matches on BLASTn, and closely related reference phage genomes. **(B)** Rectangular proteomic tree of phage vB_Kpn_ZCKp20p and 99 phages with highest ViPTree *S_G_* scores.

We used proteome-based phylogeny to compare vB_Kpn_ZCKp20p genome to ∼4,800 phages using VipTree. Phage vB_Kpn_ZCKp20p and the most closely related phages (ZCKP8 and 6691) were clustered among other unclassified siphoviruses whose bacterial hosts belong to class Gammaproteobacteria (**Figure 10A**). Phage vB_Kpn_ZCKp20p and 99 phages with highest VipTree *S_G_* were selected to construct a rectangular proteomic tree (**Figure 10B**, **Table S9**). The rectangular proteomic tree grouped phage vB_Kpn_ZCKp20p with other unclassified siphoviruses in a clade that is distinct from siphoviruses of family *Drexlerviridae* of *S_G_* ≤ 0.06.

**Figure 11:**
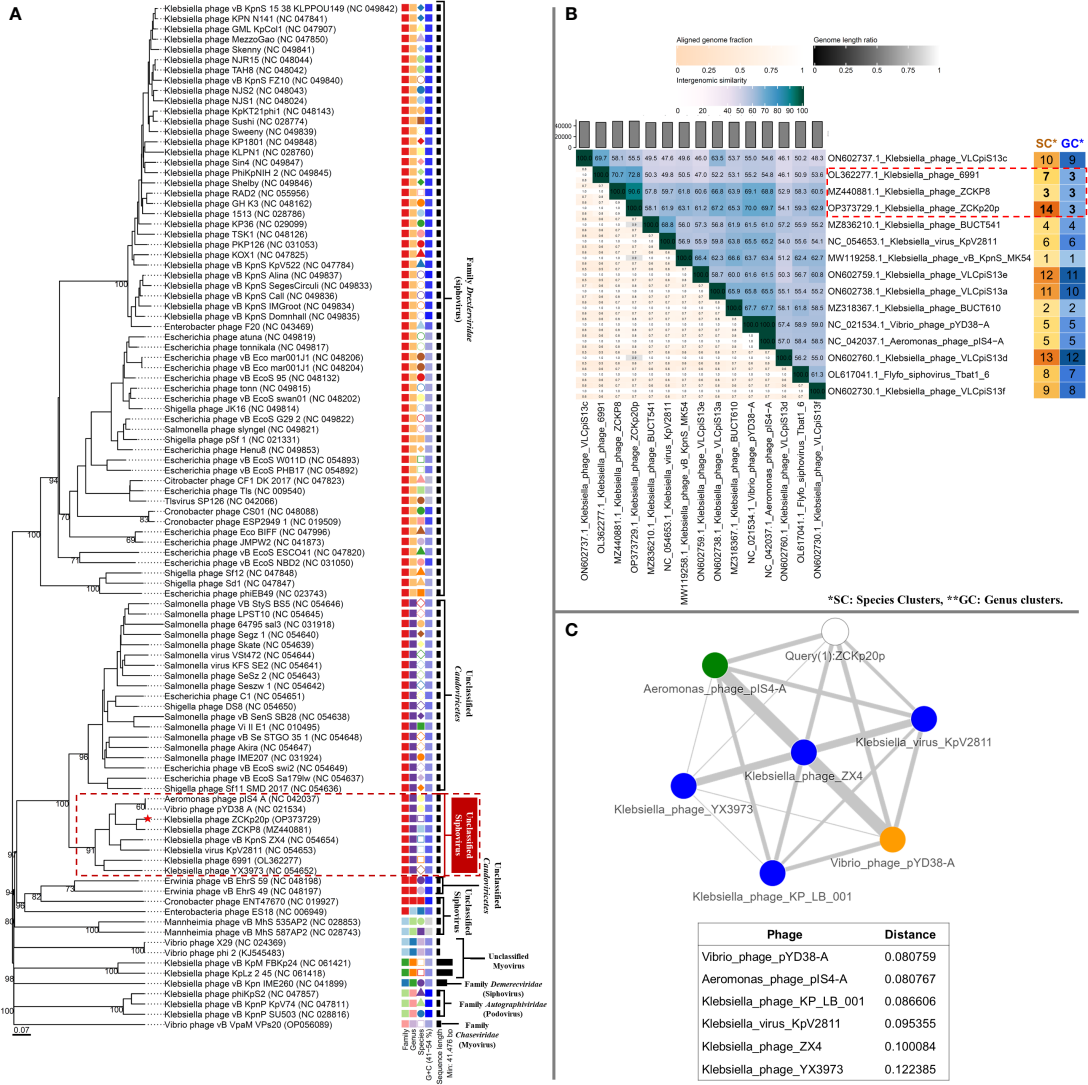
Phylogeny of phage vB_Kpn_ZCKp20p. **(A)** VICTOR genome-based phylogenetic tree conducted by nucleotides pairwise comparison of phage vB_Kpn_ZCKp20p, other *Caudoviricetes* phages, and closely related phages according to genome BLASTn top hits. The phages were clustered into 6 families, 10 genera, and they were clustered into 94 species. The GC content of the phage genomes is represented in the hue of blue color, and the genome size is presented in horizontal black lines on the right side. **(B)** VIRIDIC heatmap for the intergenomic similarity between phage vB_Kpn_ZCKp20p and closely related phages according to genome BLASTn top hits. Three factors of the alignment capacity were considered to calculate the relatedness between the phages: intergenomic similarities (hue of green to blue), the percentage of aligned genome sequence in a pair (shade of pink) and their length ratio (shade of black). **(C)** PhageCloud analysis of phage vB_Kpn_ZCKp20p genomic relationship with the closest reference phage genomes on NCBI-GenBank. Intergenomic distances were calculated by dashing based on a threshold of 0.25.

The genome-to-genome distance-based phylogenetic analysis (VICTOR analysis by the GBDP method with 30% average support and OPTSIL clustering) generated 94 species clusters, ten clusters at the genus level, and six families. VICTOR clustered phage vB_Kpn_ZCKp20p with other siphoviruses belonging to the family *Drexlerviridae*, but not at the same genus level (**Figure 11A**). At the genus level, phage vB_Kpn_ZCKp20p was grouped with other unclassified siphoviruses; however, it represented a unique species among them.

VIRIDIC was also used to compute pairwise intergenomic similarities of phage vB_Kpn_ZCKp20p and other phages, since VICTOR results had weak support (30%). Unlike VICTOR, VIRIDIC can readily analyze > 100 genomes; however, it cannot determine relationships between phages of similarity below 65%. Therefore, phages of intergenomic similarity results > 60% with phage vB_Kpn_ZCKp20p were shortlisted, and their analysis results are graphically represented as a heat map (**Figure 11B**). The calculated intergenomic similarity of VIRIDIC analysis for phage vB_Kpn_ZCKp20p versus > 600 RefSeq genomes of class *Caudoviricetes* phages are available in the supplementary material (**Table S10**).

VIRIDIC clustered phage vB_Kpn_ZCKp20p and the shortlisted 14 phages into 14 species clusters, in which all phages had distinct species. The only exception was *Aeromonas* phage pIS4-A and *Vibrio* phage pYD38-A, which were grouped under one species. At the genus level, VIRDIC clustered the phages into 12 different genera, in which phage vB_Kpn_ZCKp20p, *Klebsiella* phage ZCKP8, and *Klebsiella* phage 6991 were exclusively grouped in the same genus cluster. The intergenomic similarity of phage vB_Kpn_ZCKp20p to *Klebsiella* phages ZCKP8 and 6991 was 90.6% and 72.8%, respectively (**Figure 11B**), which qualified them to belong to the same genus (default VIRDIC similarity thresholds are 95% for species and 70% for genus).

**Figure 12:**
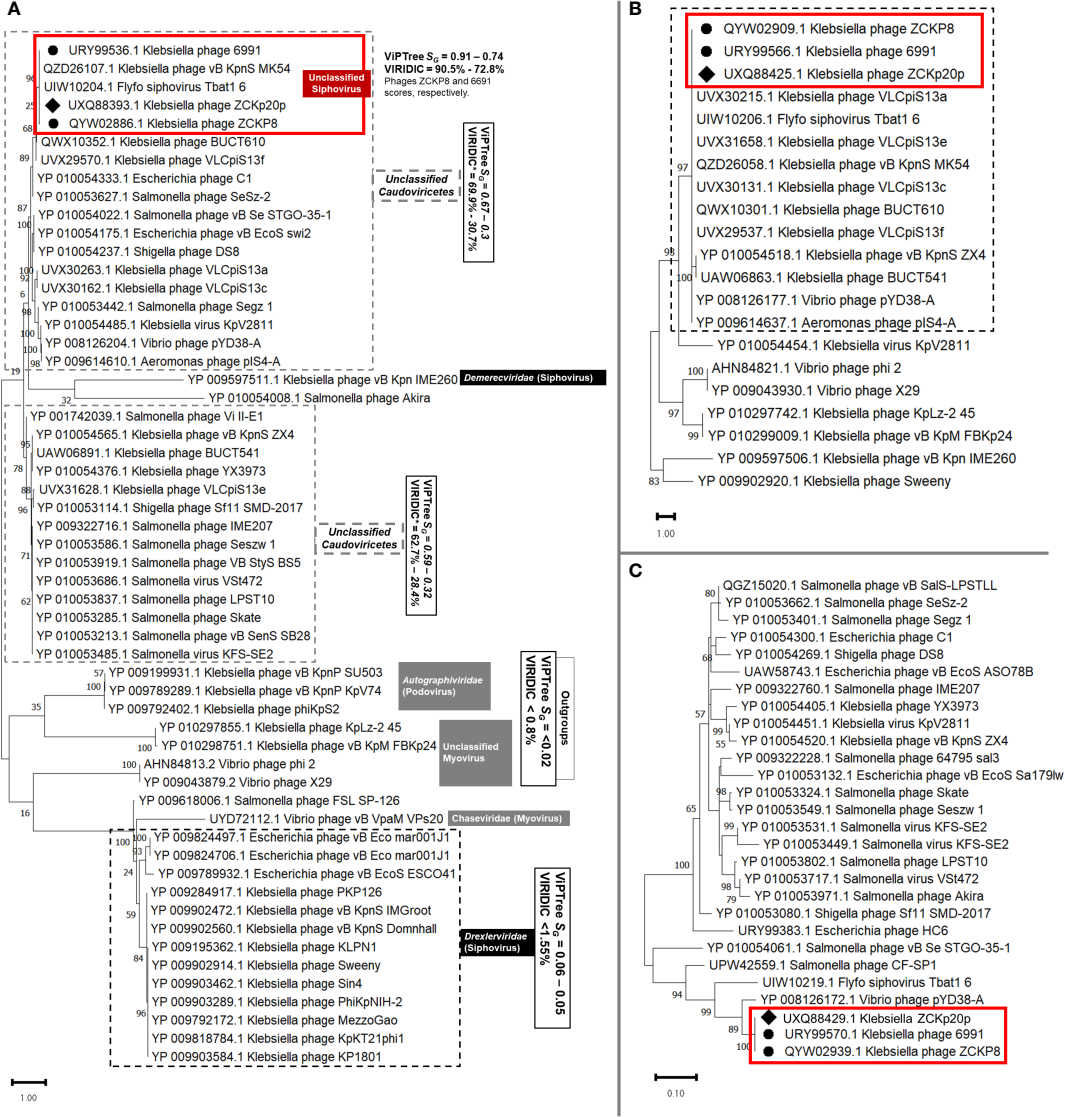
Viral conserved signature protein-based phylogenetic tree. **(A)** A phylogenetic tree for the evolutionary relatedness of the terminase large subunits of phage vB_Kpn_ZCKp20p to its corresponding proteins in other phages. **(B)** Phylogenetic tree of the major capsid protein of vB_Kpn_ZCKp20p and its orthologs in closely related phages. **(C)** Phylogenetic tree of the DNA polymerase III beta subunit of phage vB_Kpn_ZCKp20p and its orthologs in other phages. Phages included in the evolutionary analysis were chosen based on the closest relatives according to the proteomic tree and intergenomic similarity analysis. The phylogenetic distance was inferred by the Maximum likelihood method and supported with a bootstrap of 1000 replicates, and the trees were constructed by MEGA11. Phage vB_Kpn_ZCKp20p clusters were outlined in red based on the protein homology in both trees of the highest log likelihood (-12536.16, -5522.81 and -1839.71, respectively).

Another protein-protein network-based approach, PhageCloud, investigated the genomic relationship of phage vB_Kpn_ZCKp20p with phage genomes on NCBI-GenBank. Out of 5,240 analyzed complete genomes, only six phages were top matched to phage vB_Kpn_ZCKp20p with a distance range of 0.08 to 0.12 (**Figure 11C**). Thirty-one homologous genes were detected by CoreGenes5 in vB_Kpn_ZCKp20p and its top matched phages on BLASTn, which included the genes encoding terminase large subunit and major capsid proteins (**Table S11**); however, DNA polymerase wasn’t one of the identified homologs. DNA polymerase was identified as a homolog when the analysis included vB_Kpn_ZCKp20p and the most closely related phages (ZCKP8 and 6991, **Table S12**). Accordingly, phylogenetic analysis based on conserved signature proteins (i.e., terminase large subunit, major capsid protein and DNA polymerase III beta subunit) was considered. This analysis resulted in divergent classification, and only two phages (ZCKP8 and 6991) were monophyletic in the three generated phylogenetic trees (**Figure 12**). Siphoviruses of families *Drexlerviridae* and *Demerecviridae* were grouped separately from the clade of vB_Kpn_ZCKp20p in the trees of terminase and major capsid proteins (**Figures 12A, B**).

Taken together, these results strongly suggest that phage vB_Kpn_ZCKp20p shares the same genus as *Klebsiella* phages ZCKP8 and 6691, but they do not share the same species. Accordingly, we consider phage vB_Kpn_ZCKp20p as a siphovirus that belongs to a new species in the class *Caudoviricetes*.

## 4 Discussion

*K. pneumoniae* is one of the ESKAPE pathogens that are considered a threat to global public health. MDR *K. pneumoniae* strains are highly associated with hospitals, where patients are exposed to many risk factors: immunosuppression and invasive medical procedures, including endotracheal ventilation (Rice, 2008). In Egyptian hospitals, MDR *K. pneumoniae* strains are commonly isolated, particularly from ventilator-associated pneumonia, with a diverse array of antimicrobial resistance genes that limit the available therapeutic options, even the last resort antibiotics, carbapenems (Abdelwahab et al., 2021). Hence, there is an urgent demand to find alternative therapeutic approaches for *K. pneumoniae* infections, and among these approaches is phage therapy.

For therapy, an ideal phage is one that is strictly lytic, with high efficiency of killing its bacterial host, and that is safe to use in humans, i.e., with no potential risk of making bacteria neither more resistant nor more virulent. Additionally, a therapeutic bacteriophage against *K. pneumoniae* should be effective against the harder-to-treat strains of this notorious pathogen and should have as broad a host range as practically achievable (Weber-Dabrowska et al., 2016; Hyman, 2019).

In this study, six phages were isolated from urban and medical sewage. Out of these, phage vB_Kpn_ZCKp20p was selected because it had the broadest host-range with clear plaques that reflect the lytic efficiency of the phage and because it targeted a host with high *in vitro* resistance to antimicrobials. The experimental work started by collecting a rather challenging set of biofilm-producing MDR *K. pneumonia* isolates to be screened for phage susceptibility. Fortunately, but alarmingly, MDR *K. pneumoniae* isolates were not hard to find. Phage vB_Kpn_ZCKp20p was characterized and tested for its ability to lyse clinical isolates, and those susceptible ones were all armed with critical siderophores and types 1 and 3 fimbriae. These virulence factors promote bacterial growth, replication, and pathogenesis as they may deteriorate respiratory infections into bacteremia and induce inflammation, in addition to contributing to colonization of hospital devices (Nelson et al., 2007; Shon et al., 2013; Holden et al., 2016).

The antibiograms of all tested bacterial isolates revealed significant resistance, an observation in accordance with several studies that estimated the predominance of carbapenem-resistant isolates from Egyptian hospitals to range from 37.5% to 62.5% (Metwally et al., 2013). While the bacteria were somewhat susceptible to aminoglycosides, notably amikacin and gentamycin, on average, each bacterial isolate was resistant to nine antimicrobial agents.

For an objective assessment of multiresistance, we adopted the previously described MAR index score (Davis and Brown, 2016) since it is considered a reliable indicator for tracking bacterial infections and AMR. A bacterial isolate whose MAR index exceeds 0.2 is primarily believed to have developed from a high-risk source of contamination, where antimicrobials are heavily administered (Davis and Brown, 2016). In this study, all bacterial isolates had a MAR index > 0.2. We developed a modified formula for the MAR index, in which the ratio of intermediately susceptible bacteria was scored as 0.5, in addition to the resistant ones, typically scored as 1. We suggest that the modified-MAR index is more accurate and reflects the real challenge in treating MDR infection, since, in clinical practice, antimicrobial agents with intermediate bacterial susceptibility are disregarded from the therapeutic regimen because of their uncertain efficacy (Kahlmeter et al., 2019).

Curiously, a partial negative correlation was observed (*r_s_* = -0.6139) between the modified-MAR indices of bacterial isolates and their susceptibility scores to the isolated phages (**Figure 2**). In simpler terms, the bacteria that were resistant to most antibiotics were also resistant to the screened phages. The latter observation contrasts with some published suggestions that antimicrobial selective pressure drives the bacteria to a tradeoff between resistance to antibiotics *vs.* phages (Mangalea and Duerkop, 2020; Majkowska-Skrobek et al., 2021). In some studies, antimicrobial pressure enhanced phage susceptibility by reducing bacterial immunity to phages through repressing the CRISPR-Cas system. Lin et al. (2016) observed a significant inverse correlation between CRKP and harboring CRISPR-Cas loci; this was experimentally verified, as treatment of *K. pneumoniae* strain with imipenem reduced the expression of the CRISPR-Cas system. Other studies did not find a correlation between antimicrobial resistance and phage susceptibility (Kesik-Szeloch et al., 2013; Allen et al., 2017), even though an *in vitro* study reported induction in phage resistance after applying streptomycin with subinhibitory to *Pseudomonas* species (McCallin and Oechslin, 2019). Most published data describe the coevolution of the phages and their bacterial host as a complicated process more like bacteria-phage racing. There is a fitness cost to developing phage resistance, which may select for bacterial hosts that are less virulent and more susceptible to antibiotics. With all this said, we believe that there is no real contradiction between the above studies. Antimicrobial resistance is a versatile phenotype, contributed by several genetic mechanisms, including two major sets of mechanisms: acquisition of resistance genes, often horizontally, or mutation in conserved genes. So, we suggest that it is possible that plasmid- or integron-acquired resistance has endowed these bacterial strains with phage resistance as well, but this hypothesis remains to be tested (Ojala et al., 2013; Chen et al., 2022).

To sum up, we have collected a set of *K. pneumoniae* isolates, with high virulence and a wide range of antimicrobial resistance patterns, and we isolated six candidate phages, that mostly affected bacteria with intermediate multiresistance potential (MAR indices of 0.39-0.69). As stated above, phage vB_Kpn_ZCKp20p was selected because of its relatively broad host range, but also because of its lytic potential.

Phage vB_Kpn_ZCKp20p demonstrated a short latent period with medium size burst size and significantly reduced the viable bacteria at different MOIs. At MOI = 1, no bacterial recovery was observed for more than three hours. The antibacterial efficiency of phage vB_Kpn_ZCKp20p was supported by potent antibiofilm activity observed *in vitro* at all tested MOIs, as it inhibited the formation of new biofilm and degraded a 48-hour-old (mature) biofilm. As will be discussed in detail below, whole-genome sequencing and analysis of phage vB_Kpn_ZCKp20p demonstrated the presence of intact putative proteins involved in killing the bacterial host, including an endopeptidase, a lysin, a holin, and a depolymerase.

Among the phage genomes with the highest similarity to phage vB_Kpn_ZCKp20p (according to top BLASTn hits, **Table S6**), only three *Klebsiella* phages (ZCKP8 (Fayez et al., 2021), BUCT610 (Pu et al., 2022), and vB_KpnS_MK54 (Lu et al., 2022)) were characterized and published. Hence, in the following paragraphs, we discuss the characteristic properties of phage vB_Kpn_ZCKp20p compared to related published phages and other *Klebsiella* siphoviruses.

The short latent period and medium burst size of phage vB_Kpn_ZCKp20p are comparable to many of the published *Klebsiella* siphoviruses (Tabassum et al., 2018; Feng et al., 2021; Lu et al., 2022; Pu et al., 2022). A few phages had burst sizes > 200 PFU/cell (Cai et al., 2019; Horvath et al., 2020) and many others had smaller (< 85 PFU/cell) burst sizes (Kesik-Szeloch et al., 2013; Li et al., 2020; Balcão et al., 2022; Lu et al., 2022).

Applying an isolated phage requires stable replicability and lytic activity within a wide range of temperatures and pH. Most published *Klebsiella* phages were assayed for stability after one to two hours, whereas phage vB_Kpn_ZCKp20p stability was evaluated after 24 h to verify its infectivity and replicability threshold under stressful conditions. Like other *Klebsiella* phages ZCKP8, BUCT610, and P545 (Li et al., 2020; Fayez et al., 2021; Pu et al., 2022), phage vB_Kpn_ZCKp20p demonstrated constant stability at storage over a wide temperature range (-20 to 60°C). At 70°C, phage vB_Kpn_ZCKp20p remained infective for 2 h, similar to *Klebsiella* phage BUCT556A (Feng et al., 2021), but its stability was extended up to 24 h at the same temperature, which wasn’t tested for most other phages.

Regarding the effect of pH on phage stability, phage vB_Kpn_ZCKp20p was significantly active at a neutral to slightly alkaline pH of 7-8. Beyond neutral pH, phage vB_Kpn_ZCKp20p was stable at a wide range of both the acidic and alkaline spectrum (pH of 2 to 12), with a limited reduction in viability after 24 h of exposure. Consequently, phage vB_Kpn_ZCKp20p had comparable pH stability with *Klebsiella* phage BUCT610 (Pu et al., 2022), while other *Klebsiella* phages (ZCKP8, vB_KpnS_MK54, P545) demonstrated stability at a narrower pH range (Li et al., 2020; Fayez et al., 2021; Lu et al., 2022).

Phage vB_Kpn_ZCKp20p significantly inhibited the formation of fresh biofilm and was able to disrupt a maturely formed biofilm. This activity was comparable to siphophage TSK1 and *Klebsiella* phage P545 (Tabassum et al., 2018; Li et al., 2020). Of note, the phage had no measurable cytotoxic activity against human skin fibroblasts, even at high concentrations. Other studies also tested and confirmed that phages lack cytotoxic effects, which supports their use for therapeutic applications in humans (Shen et al., 2012; Rahimzadeh Torabi et al., 2021). An increased viability of phage-treated cells was observed by Chung et al. (2010) and Henein et al. (2016), which could be related to enhanced cell attachment due to phage exposure.

One major issue with any newly isolated bacteriophage is the uncertainty about its safety and the risk that it may potentiate bacterial resistance or virulence. Before the genomics era, the only way to ensure safety was to keep testing the phage for any potential risk to human health, which is practically prohibitive, since there is no set threshold for how long it can be tested or how many potential risks may occur in the future. The advancement of sequencing technologies in the past two decades made sequencing bacteriophage genomes feasible for most laboratories. Thus, using genomic analysis and bioinformatics could cut down the safety analysis efforts by pinpointing potential risks within the genome, such as lysogeny-encoding genes, and virulence or resistance genes that may be transduced to bacteria (Weber-Dabrowska et al., 2016; Hyman, 2019). Obviously, this is not a fail-proof operation, since many genes remain of unknown encoded functions (genomic dark matter); still, it substantially reduces the limitless trial and error efforts to test for safety.

Various tools analyzed the whole genome sequence of phage vB_Kpn_ZCKp20p, and all analysis results support the therapeutic potential of the phage, because no known genes encoding virulence factors or antimicrobial resistance were detected. In addition, no evidence for a temperate lifestyle was found. The low probability of a lysogenic potential, predicted by BACPHLIP, is possibly due to a low gene flow of some putative genes, including RNA-guided endonuclease TnpB family protein, DNA-binding protein, exodeoxyribonuclease VIII, and DNA helicase (ORFS 59, 74, 77, 80, and 82, **Table S4**). Moreover, genes encoding putative antimicrobial peptides were identified, including holins whose predicted topologies were consistent with class II holins (Young and Bläsi, 1995; Barenboim et al., 1999). The cluster of adjacent genes encoding endolysin-holin-endopeptidase Rz was predicted in phage vB_Kpn_ZCKp20p genome, which corresponds to the tailed phages conical lysis at the end of their lytic cycle (Bai et al., 2020; Palmer et al., 2021). Holins conically form micron-scale pores in the cytoplasmic membrane through which endolysins leak into and disrupt peptidoglycan. Finally, the endopeptidase degrades the outer membrane of the Gram-negative cell wall (Young, 1992; Young, 2014).

The genomic analysis also predicted depolymerase activity associated with the putative tail spike protein (Liu et al., 2022). Depolymerases can degrade bacterial polysaccharide capsules, and significantly inhibit or remove biofilm (Hughes et al., 1998; Broeker and Barbirz, 2017; Tabassum et al., 2018). Just taking off the capsular and biofilm shields would enhance the antibacterial effect of the host immune system and the administered antibiotic, even if the phage does not proceed to full lysis (Hsieh et al., 2017). The in silico predictions of the depolymerase activity complied with the observed *in vitro* antibiofilm activity of phage vB_Kpn_ZCKp20p. Finally, the in silico analysis with different methods suggesting the safety of phage vB_Kpn_ZCKp20p by demonstrating the lack of any bacterial virulence or antimicrobial resistance genes.

The phylogenetic analysis and classification of phages have always been challenging owing to genomic mosaicism, which is an authentic attribute of phages due to the high frequency of mutation and horizontal gene transfer, in addition to the absence of a single universally conserved gene in all phages (Hendrix, 2003; Lima-Mendez et al., 2008; Khot et al., 2020). Therefore, the phylogeny and relatedness of phage vB_Kpn_ZCKp20p to other phages were investigated by various methods, as recommended in the literature (Grose and Casjens, 2014; Aziz et al., 2018). Proteome-based phylogeny clustered vB_Kpn_ZCKp20p with other unclassified siphoviruses; however, it was not monophyletic with phages of family *Drexlerviridae*. These phages possess common characters, such as morphotype, host type, genome size (∼48.9 Kbp, GC% ∼46), ∼79 encoding genes and 0-2 t-RNAs, as described in the ICTV 2018-2019 update (Adriaenssens et al., 2020). Proteomic analysis is the most reliable to resolve taxonomic distant relationships of phages particularly at family level (Rohwer and Edwards, 2002; Turner et al., 2021).

Therefore, further analyses were conducted on nucleotide sequence, as nucleotide-level analysis is the most suitable approach for classifying closely related phages (Grose and Casjens, 2014). Accordingly, ANIb was used to identify the closest phages to phage vB_Kpn_ZCKp20p to be compared on genomic and proteomic levels (*Klebsiella* phages ZCKP8 and 6691). Unlike ANIb, other methods (VICTOR and VIRIDIC) clustered phage vB_Kpn_ZCKp20p and the closest two phages in different species but still under the same genus. To err on the side of caution, we considered VICTOR (GBDP) and VIRIDIC classification, rather than ANIb, as they are particularly designed for viruses—according to ICTV recommendation (Meier-Kolthoff and Göker, 2017; Moraru et al., 2020).

Additionally, network-based phylogeny (PhageCloud) was used as it takes into account the mosaic nature of phage genomes, thus contributing to a better understanding of phage evolutionary relationships (Dion et al., 2020; Khot et al., 2020; Rangel-Pineros et al., 2021). However, the most closely related phages (*Klebsiella* phages ZCKP8 and 6991) were not included in the PhageCloud analysis because they were not listed as reference sequences in the GenBank database, at the time of analysis (July 11^th^, 2022) and until this manuscript was submitted (October 7^th^, 2022).

The identified 31 orthologous genes supported the pairwise genome comparison, with extra evidence that vB_Kpn_ZCKp20p most likely represents a new family with other closely related unclassified siphoviruses; this is also consistent with the proteome-based analysis results. Signature gene analysis was recommended by Turner et al. (2021) to resolve errors caused by pairwise genome analysis. The phylogenetic trees of terminase proteins (large subunit), major capsid protein, and DNA polymerases suggested that phage vB_Kpn_ZCKp20p most likely belongs to the same genus of *Klebsiella* phage ZCKP8 and *Klebsiella* phage 6991 (Tabassum et al., 2018; Sofy et al., 2021). This was even more supported by the results of their proteome comparison that revealed high similarity and coverage (≥ 95%) of 58-51 proteins, including other proteins like capsid decoration, tail tape measure and tail spike proteins, but other proteins (some enzymes and portal protein) had a low similarity. Therefore, we conclude that phage vB_Kpn_ZCKp20p represents a new species and shares the same genus of *Klebsiella* phages ZCKP8 and 6691.

One limitation of the study is related to the host range of phage vB_Kpn_ZCKp20p. Although the phage was selected as it had the broadest range against challenged bacterial isolates, it was not able to lyse the most resistant ones. One potential concern regarding the observed negative correlation between antibiotic resistance and susceptibility to the isolated phages, is that other factors related to phage attachment to host cell surface receptors need to be included, and a wider pool of bacterial hosts should be tested.

Another area for expanding this study is to explore other isolated phages with good host ranges and promising antibacterial activity. Future work will characterize other phages isolated in this study, e.g., ZCKp24r2. Additional future research would implement cloning and expression of the identified putative proteins with antibacterial or antibiofilm potential and test their activity experimentally.

## 5 Conclusion

We isolated phage vB_Kpn_ZCKp20p, which is a virulent phage against *K. pneumoniae*, with no signs of safety concerns in humans, and no genomic evidence of transducing resistance of virulence to bacteria. We suggest it to be a novel species in the former family *Siphoviridae*, and it most likely represents a new family of siphovirus morphotype that has not been ratified yet by ICTV. Phage vB_Kpn_ZCKp20p was highly stable at a wide range of temperatures and pH values. These properties are critical for flexible formulation so that the phage can fit various dosage forms and applications. Phage vB_Kpn_ZCKp20p is a promising candidate for therapeutic/infection control purposes as a single or in a cocktail of other phages against MDR *K. pneumoniae* pathogens, with no detected cytotoxicity against human fibroblasts. Additionally, phage vB_Kpn_ZCKp20p is a potential source for several putative antibacterial and antibiofilm proteins or peptides that were identified through the functional annotation of individual genes or sets of adjacent genes.

## Data availability statement

The datasets presented in this study can be found in the article and **Supplementary Material**. The whole-genome sequence of phage vB_Kpn_ZCKp20p has been deposited under accession number OP373729 in the NCBI database.

## Author contributions

RA, RS, AE-S conceived the study and supervised different stages of the work. BZ, RS, and AE-S designed experiments. BZ and NF performed *in vitro* experiments. BZ analyzed results, created the figures, and performed statistical analysis. BZ and RA conducted bioinformatics analysis. BZ and RS analyzed biofilm experiments. AE-S coordinated the different stages of the work and provided equipment and reagents for all laboratory experiments. BZ drafted the manuscript. RA, RS, AE-S revised the draft. All authors contributed to the article and approved the submitted version.
