# Supplementary material for: Characterization and comprehensive genome analysis of novel bacteriophage, vB_Kpn_ZCKp20p, with lytic and anti-biofilm potential against clinical multidrug-resistant Klebsiella pneumoniae
Source: Front Cell Infect Microbiol. 2023 Jan 23;13:1077995. doi: 10.3389/fcimb.2023.1077995 (PMC9901506; doi:10.3389/fcimb.2023.1077995)
Supplement: Supplementary file 1 [file DataSheet_1.pdf]

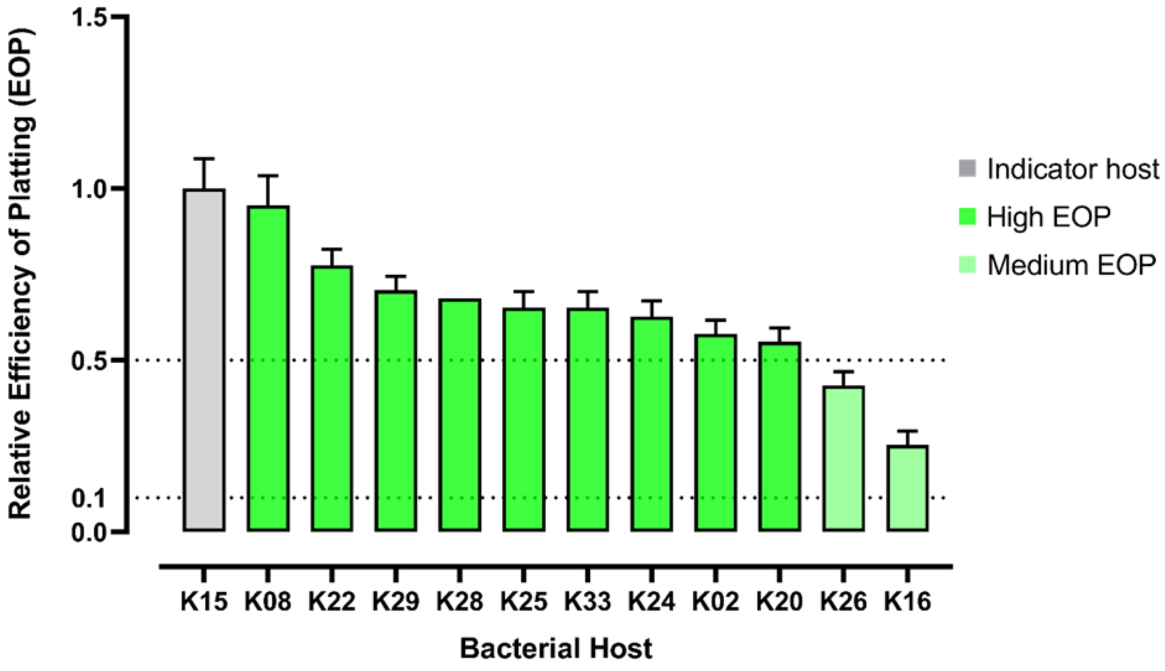

Supplementary Figure S1. Relative efficiency of plating (EOP) of phage vB_Kpn_ZCKp20p host range, calculated according to Kutter, 2009. The indicator host of the highest EOP is represented by a gray bar. The relative EOP of all other bacterial hosts was estimated as a ratio to the indicator host. Phage titers were determined by the Double Agar Overlay Plaque assay. The mean of triplicate results of each bacterial host was plotted, and error bars represent their standard deviations.

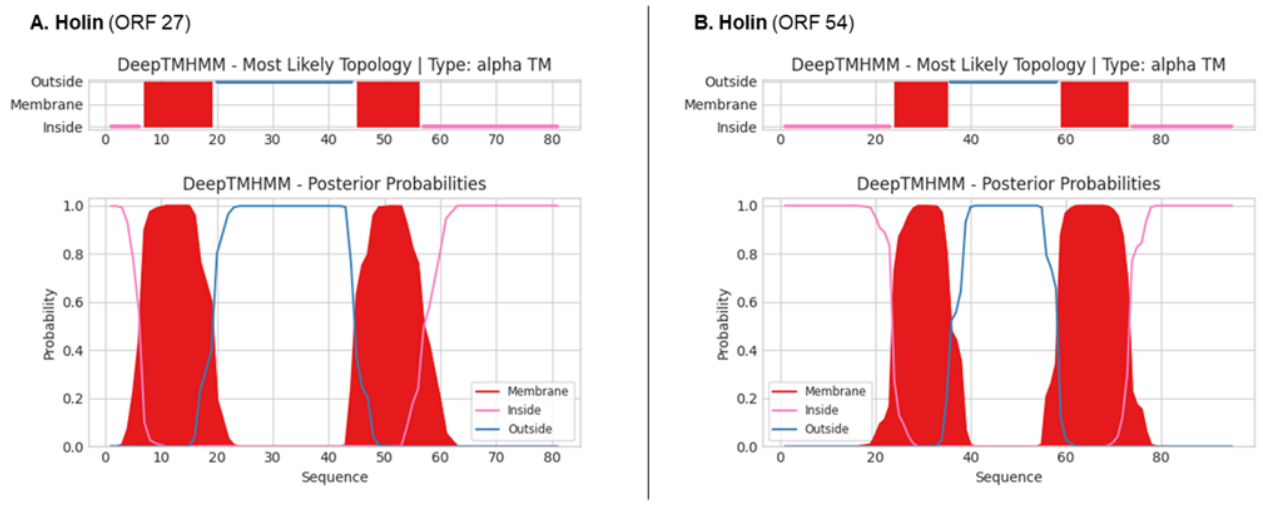

Supplementary Figure S2. Predicted topology of ORFs 27 and 54 (putative class II holins). DeepTMHMM was used to predict the topology of ORF 27 **(A)** and ORF 54 **(B)**. The top part of each chart represents the topology of predicted domains in correspondence to the amino acid sequence: transmembrane (in red), intracellular (in pink), and extracellular (in blue). The probability of predicted topology is presented in the bottom part. The topologies of holin in ORFs 27 and 54 were predicted by 100%.

frontiers

Supplementary Table S1. List of antimicrobial agents and their classes

Antimicrobial agent	Class	Abbreviation	Concentration*
Piperacillin	Penicillins	PRL	100 µg
Piperacillin-tazobactam	Beta-Lactam Combination	TZP	100/10 µg
Cefuroxime	2nd Generation Cephalosporin	CXM	30 µg
Cefepime	4th Generation Cephalosporin	FEB	30 µg
Doripenem	Carbapenem	DOR	10 µg
Meropenem		MPM	10 µg
Ertapenem		ETP	10 µg
Imipenem		IMP	10 µg
Aztreonam	Monobactam	ATM	30 µg
Azithromycin	Macrolides	AZM	15 µg
Tobramycin	Aminoglycoside	TOB	10 µg
Kanamycin		K	30 µg
Gentamicin		CN	10 µg
Amikacin		AK	30 ug
Ciprofloxacin	Fluoroquinolone	CIP	5 µg
Levofloxacin		LEV	5 µg
Trimethoprim-Sulfamethoxazole	Sulfonamides	SXT	1.25/23.75 µg
Doxycycline	Tetracycline	DOX	30 µg

* Oxoid, UK manufactured all mentioned antibiotic discs.

Supplementary Table S2. Primer sequences and sizes of PCR products for virulence gene screening

Gene	Protein: name and function	Gene Conservatio	Primer Sequence Sequence (5' - 3')	Ta (°C)	Amplicon Size
entB	It encodes isochorismatase for the biosynthesis of the siderophore enterobactin (Ent).	Core Genome	AAACAGCGCGATGATGGAGA<br>ACCAGCACGGTATCGTCTTC	52.5	222
iutA	It encodes ferric aerobactin receptor, responsible for aerobactin (Iuc) transport and iron ion homeostasis.	Accessory Genome	GTTGCGATTCTACCCGTTCC<br>GCCCGGTGGTGTAAATCTTC	53	248
*irp2**	It encodes the synthetase (HMWP2) for the siderophore yersiniabactin (Ybt) biosynthesis.	Accessory Genome	GCTACAATGGGACAGCAACGAC<br>GCAGAGCGATACGGAAAATGC	58	230
fimH	It encodes type 1 fimbriae, cell adhesion.	Core Genome	GTCGAGTTTTTCAGGCACCG<br>CACGTCGTTGTTGGCGTAAA	52	259
mrkD	It encodes type 3 fimbriae, cell adhesion and biofilm formation.	Core Genome	CACCTCCTACGACTGGGAGA<br>AGCGGTATGGTGATGTAGCG	54	435

*** All primers are designed specifically for this study, except irp2 primers, which were previously designed (Garza-Ramos *et al.*, 2018).**

The thermocycler (BIO-RAD T100, Singapore) conditions were adjusted to 5 min for both the initial denaturation time at 95°C and the final extension time at 72°C, and 35 cycles of reaction were implemented.

Supplementary Table S3. Levels of the relative efficiency of plating

EOP Level	The ratio of plaque count	
	**From**	**To**
High production	≥ 0.5	≥ 1
Medium production	0.1	< 0.5
Low production	0.001<	< 0.1
No production	0	≤ 0.001

Supplementary Table S4. Annotated CDS of *Klebsiella* phage vB_Kpn_ZCKp20p

Colour key in the genomic map	ORF/ CDS No.	Position (nt)		Length bp	Strand	Product	Predicted function	Functional Category	RAST	InterPro	HHPred	UNIPROT	Blastp
		Start	Stop										
	1	289	474	186	(+)	Hypothetical protein	Unknown	Hypothetical protein	☑	☐	☐	☐	☐
	2	490	768	279	(+)	Hypothetical protein	Unknown	Hypothetical protein	☑	☐	☐	☐	☐
	3	775	1158	384	(+)	Hypothetical protein	Unknown	Hypothetical protein	☑	☐	☐	☐	☐
	4	1155	1439	285	(+)	Hypothetical protein	Unknown	Hypothetical protein	☑	☐	☐	☐	☐
	5	1436	1795	360	(+)	Hypothetical protein	Unknown	Hypothetical protein	☑	☐	☐	☐	☐
	6	1795	1923	129	(+)	Hypothetical protein	Unknown	Hypothetical protein	☑	☐	☐	☐	☐
	7	1928	2779	852	(+)	Phage protein	Unknown	Hypothetical protein	☑	☐	☐	☐	☐
	8	2776	2868	93	(+)	Hypothetical protein	Unknown	Hypothetical protein	☑	☐	☐	☐	☐
	9	2872	3030	159	(+)	Hypothetical protein	Unknown	Hypothetical protein	☑	☐	☐	☐	☐
	10	3030	3224	195	(+)	Hypothetical protein	Unknown	Hypothetical protein	☑	☐	☐	☐	☐
	11	3304	3753	450	(+)	Phage protein	Unknown	Hypothetical protein	☑	☐	☐	☐	☐
	12	3743	5167	1425	(+)	Phage terminase, large subunit	Endonuclease, Hydrolase, Nuclease	DNA genome packaging	☑	☐	☐	☐	☐
	13	5188	5478	291	(–)	Hypothetical protein	Unknown	Hypothetical protein	☑	☐	☐	☐	☐
	14	5567	5806	240	(+)	Phage protein	Unknown	Hypothetical protein	☑	☐	☐	☐	☐
	15	5803	5997	195	(+)	Hypothetical protein	Unknown	Hypothetical protein	☑	☐	☐	☐	☐
	16	5997	6185	189	(+)	Hypothetical protein	Unknown	Hypothetical protein	☑	☐	☐	☐	☐
	17	6185	6385	201	(+)	Hypothetical protein	Unknown	Hypothetical protein	☑	☐	☐	☐	☐
	18	6395	6670	276	(+)	Hypothetical protein	Unknown	Hypothetical protein	☑	☐	☐	☐	☐
	19	6788	6907	120	(+)	Hypothetical protein	Unknown	Hypothetical protein	☑	☐	☐	☐	☐
	20	6955	8409	1455	(+)	Phage portal (connector) protein	Genome packaging, neck/tail attachment	Capsid structure	☑	☐	☐	☐	☐
	21	8390	9334	945	(+)	Phage minor capsid protein	Reinforced post-assembly	Capsid structure	☐	☑	☑	☑	☑
	22	9342	9464	123	(–)	Hypothetical protein	Unknown	Hypothetical protein	☑	☐	☐	☐	☐
	23	9464	10327	864	(–)	Hypothetical protein	Unknown	Hypothetical protein	☑	☐	☐	☐	☐
	24	10324	10455	132	(–)	Hypothetical protein	Unknown	Hypothetical protein	☑	☐	☐	☐	☐
	25	10455	10745	291	(–)	Hypothetical protein	Unknown	Hypothetical protein	☑	☐	☐	☐	☐
	26	10844	11281	438	(+)	Phage endolysin (EC 3.2.1.17)	Peptidoglycan hydrolases	Host cell wall lysis	☑	☐	☐	☐	☐
	27	11278	11523	246	(+)	Holin	Forming holes in the inner membrane of host cell wall	Host cell wall lysis	☐	☐	☐	☑	☑
	28	11498	11992	495	(+)	Phage endopeptidase (EC 3.4.-.-) Rz	Peptidoglycan hydrolases	Host cell wall lysis	☑	☐	☐	☐	☐
	29	11982	12125	144	(+)	Hypothetical protein	Unknown	Hypothetical protein	☑	☐	☐	☐	☐
	30	12223	12462	240	(–)	Hypothetical protein	Unknown	Hypothetical protein	☑	☐	☐	☐	☐
	31	12455	12667	213	(–)	Hypothetical protein	Unknown	Hypothetical protein	☑	☐	☐	☐	☐
	32	12667	13053	387	(–)	Hypothetical protein	Unknown	Hypothetical protein	☑	☐	☐	☐	☐
	33	13056	13250	195	(–)	Hypothetical protein	Unknown	Hypothetical protein	☑	☐	☐	☐	☐
	34	13250	13450	201	(–)	Hypothetical protein	Unknown	Hypothetical protein	☑	☐	☐	☐	☐
	35	13547	13762	216	(+)	Hypothetical protein	Unknown	Hypothetical protein	☑	☐	☐	☐	☐

Supplementary Table S4. Annotated CDS of *Klebsiella* phage vB_Kpn_ZCKp20p

Colour key in the genomic map	ORF/ CDS No.	Position (nt)		Length bp	Strand	Product	Predicted function	Functional Category	RAST	InterPro	HHPred	UNIPROT	Blastp
		Start	Stop										
	36	13846	14037	192	(+)	Hypothetical protein	Unknown	Hypothetical protein	☑	☐	☐	☐	☐
	37	14034	14258	225	(+)	Hypothetical protein	Unknown	Hypothetical protein	☑	☐	☐	☐	☐
	38	14255	14491	237	(+)	Zinc-binding domain (ZBD) of primase-helicase	Template recognition, a polymerase domain (catalytic core), and a domain that interacts with the replicative helicase	DNA replication / repair	☐	☐	☐	☑	☑
	39	14481	14951	471	(+)	Hypothetical protein	Unknown	Hypothetical protein	☑	☐	☐	☐	☐
	40	14939	15229	291	(+)	Hypothetical protein	Unknown	Hypothetical protein	☑	☐	☐	☐	☐
	41	15230	15574	345	(+)	Phage protein	Unknown	Hypothetical protein	☑	☐	☐	☐	☐
	42	15757	17157	1401	(+)	Unclassified head protein / Procapsid Core Protein Serine Protease	Serine protease involved in capsid assembly and maturation	Capsid assembly	☑	☐	☑	☑	☑
	43	17157	17621	465	(+)	Capsid decoration protein	Stabilizing capsid structure	Capsid assembly	☐	☐	☑	☑	☑
	44	17633	18721	1089	(+)	Unclassified head protein / Major capsid protein	Capisd assembly	Capsid structure	☑	☐	☑	☑	☑
	45	18764	18958	195	(+)	Hypothetical protein	Unknown	Hypothetical protein	☑	☐	☐	☐	☐
	46	19000	19818	819	(+)	Hypothetical protein	Unknown	Hypothetical protein	☑	☐	☐	☐	☐
	47	19848	20030	183	(–)	Hypothetical protein	Unknown	Hypothetical protein	☑	☐	☐	☐	☐
	48	20033	20569	537	(–)	DNA polymerase III beta subunit (EC 2.7.7.7)	DNA replication	DNA replication / repair	☑	☐	☐	☐	☐
	49	20566	20742	177	(–)	Hypothetical protein	Unknown	Hypothetical protein	☑	☐	☐	☐	☐
	50	20739	21086	348	(–)	Hypothetical protein	Unknown	Hypothetical protein	☑	☐	☐	☐	☐
	51	21086	21901	816	(–)	Phage protein	Unknown	Hypothetical protein	☑	☐	☐	☐	☐
	52	21909	22025	117	(–)	ParB/RepB/Spo0J family partition protein	Transcriptional regulator	DNA replication / repair	☐	☑	☑	☑	☑
	53	22022	22318	297	(–)	Hypothetical protein	Unknown	Hypothetical protein	☑	☐	☐	☐	☐
	54	22543	22830	288	(+)	Holin	Forming holes in the inner membrane of host cell wall	Host cell wall lysis	☐	☑	☑	☑	☑
	55	22808	23200	393	(+)	Phage protein	Unknown	Hypothetical protein	☑	☐	☐	☐	☐
	56	23197	23331	135	(+)	Hypothetical protein	Unknown	Hypothetical protein	☑	☐	☐	☐	☐
	57	23319	23663	345	(+)	Minor capsid protein	Unknown	Capsid structure	☐	☐	☑	☐	☑
	58	23667	24083	417	(+)	Minor capsid protein	Unknown	Capsid structure	☐	☐	☑	☐	☑
	59	24132	25262	1131	(–)	Transposase: RNA-guided endonuclease TnpB family protein	Guide RNA to bind and cleave guide-complementary DNA in a target adjacent motif-dependent manner	DNA genome packaging	☑	☑	☑	☐	☑
	60	25380	25763	384	(+)	Minor capsid protein	Unknown	Capsid structure	☐	☐	☑	☐	☑
	61	25770	25839	70	(–)	tRNA-Arg-TCT	Unknown	tRNA	☑				
	62	26297	27049	753	(+)	Phage fibritin (Wac) protein	Chaperone involved in tail fiber contractile mechanical	Tail structure	☑	☐	☐	☐	☐
	63	27089	27757	669	(–)	Phage protein (ACLAME family 536)	Unknown	Hypothetical protein	☑	☐	☐	☐	☐

frontiers

Supplementary Table S4. Annotated CDS of *Klebsiella* phage vB_Kpn_ZCKp20p

Colour key in the genomic map	ORF/ CDS No.	Position (nt) Start	Stop	Length bp	Strand	Product	Predicted function	Functional Category	RAST	InterPro	HHPred	UNIPROT	Blastp
	64	27757	28008	252	(–)	Hypothetical protein	Unknown	Hypothetical protein	☑	☐	☐	☐	☐
	65	28005	28490	486	(–)	Phage HNH homing endonuclease (ACLAME 27)	Endonuclease, Hydrolase, Nuclease	DNA genome packaging	☑	☐	☐	☐	☐
	66	28477	28611	135	(–)	Hypothetical protein	Unknown	Hypothetical protein	☑	☐	☐	☐	☐
	67	28709	29416	708	(+)	Putative tail protein	Unknown	Tail structure	☐	☐	☐	☐	☑
	68	29416	32709	3294	(+)	Phage tail tape measure protein	Stopping the tail tube polymerization	Tail structure	☑	☐	☐	☐	☐
	69	32709	33179	471	(+)	Baseplate wedge protein	Tail assembly	Tail structure	☐	☐	☑	☐	☑
	70	33179	33649	471	(+)	Phage protein	Unknown	Hypothetical protein	☑	☐	☐	☐	☐
	71	34018	36498	2481	(+)	Minor structural protein/ Host specificity factor TipJ family phage tail protein	Unknown	Tail structure	☐	☐	☐	☑	☑
	72	36537	39020	2484	(+)	Phage tailspike protein with depolymerase activity	Binding to the polymannose O-antigen accelerating adsorption of T5 phage	Tail structure	☐	☑	☑	☑	☑
	73	39061	39255	195	(–)	Hypothetical protein	Unknown	Hypothetical protein	☑	☐	☐	☐	☐
	74	39294	39797	504	(–)	Single-stranded DNA-binding protein, phage-associated	DNA binding protein	DNA Replication / Repair	☑	☐	☐	☐	☐
	75	39807	40844	1038	(–)	P-loop containing nucleoside triphosphate hydrolase (AAA domain)	DNA metabolism	DNA genome packaging	☐	☑	☐	☑	☑
	76	40841	41116	276	(–)	Hypothetical protein	Unknown	Hypothetical protein	☑	☐	☐	☐	☐
	77	41140	42096	957	(–)	Exodeoxyribonuclease VIII	Endonuclease, Hydrolase, Nuclease	DNA replication / repair	☑	☐	☐	☐	☐
	78	42099	42542	444	(–)	Hypothetical protein	Unknown	Hypothetical protein	☑	☐	☐	☐	☐
	79	42590	43006	417	(–)	Nuclease	Endonuclease, Hydrolase, Nuclease	DNA genome packaging	☐	☑	☐	☑	☑
	80	42993	44900	1908	(–)	Phage-associated DNA helicase	DNA and RNA metabolism	DNA replication / repair	☑	☐	☐	☐	☐
	81	44909	45682	774	(–)	Phage-associated homing endonuclease	Endonuclease, Hydrolase, Nuclease	DNA genome packaging	☐	☐	☐	☐	☐
	82	45755	46648	894	(+)	Phage-associated DNA primase/helicase	DNA and RNA metabolism	DNA replication / repair	☑	☐	☐	☐	☐
	83	46662	46895	234	(–)	Activating signal cointegrator (Asch) domain protein	DNA and RNA metabolism	DNA transcription	☑	☐	☐	☐	☐
	84	46892	48445	1554	(–)	Phage protein	Unknown	Hypothetical protein	☑	☐	☐	☐	☐
	85	48445	48678	234	(–)	Hypothetical protein	Unknown	Hypothetical protein	☑	☐	☐	☐	☐

frontiers

Supplementary Table S5. Phage Depolymerase Finder (PhageDPO) results

ID	ANN7185 DPO Prediction (%)
fiG|2202568.10.peG.71	100
fiG|2202568.10.peG.32	100
fiG|2202568.10.peG.70	58
fiG|2202568.10.peG.46	42
fiG|2202568.10.peG.61	26
fiG|2202568.10.peG.44	22
fiG|2202568.10.peG.79	3
fiG|2202568.10.peG.42	1
fiG|2202568.10.peG.9	0
fiG|2202568.10.peG.84	0
fiG|2202568.10.peG.83	0
fiG|2202568.10.peG.82	0
fiG|2202568.10.peG.81	0
fiG|2202568.10.peG.80	0
fiG|2202568.10.peG.8	0
fiG|2202568.10.peG.78	0
fiG|2202568.10.peG.77	0
fiG|2202568.10.peG.76	0
fiG|2202568.10.peG.75	0
fiG|2202568.10.peG.74	0
fiG|2202568.10.peG.73	0
fiG|2202568.10.peG.72	0
fiG|2202568.10.peG.7	0
fiG|2202568.10.peG.69	0
fiG|2202568.10.peG.68	0
fiG|2202568.10.peG.67	0
fiG|2202568.10.peG.66	0
fiG|2202568.10.peG.65	0
fiG|2202568.10.peG.64	0
fiG|2202568.10.peG.63	0
fiG|2202568.10.peG.62	0
fiG|2202568.10.peG.60	0
fiG|2202568.10.peG.6	0
fiG|2202568.10.peG.59	0
fiG|2202568.10.peG.58	0
fiG|2202568.10.peG.57	0
fiG|2202568.10.peG.56	0
fiG|2202568.10.peG.55	0
fiG|2202568.10.peG.54	0
fiG|2202568.10.peG.53	0
fiG|2202568.10.peG.52	0
fiG|2202568.10.peG.51	0
fiG|2202568.10.peG.50	0
fiG|2202568.10.peG.5	0

ID	SVM4311 DPO Prediction (%)
fiG|2202568.10.peG.71	100
fiG|2202568.10.peG.70	98
fiG|2202568.10.peG.46	83
fiG|2202568.10.peG.44	64
fiG|2202568.10.peG.43	58
fiG|2202568.10.peG.58	57
fiG|2202568.10.peG.20	44
fiG|2202568.10.peG.32	39
fiG|2202568.10.peG.55	32
fiG|2202568.10.peG.42	27
fiG|2202568.10.peG.11	20
fiG|2202568.10.peG.68	19
fiG|2202568.10.peG.69	17
fiG|2202568.10.peG.61	15
fiG|2202568.10.peG.54	11
fiG|2202568.10.peG.66	10
fiG|2202568.10.peG.2	9
fiG|2202568.10.peG.3	9
fiG|2202568.10.peG.12	9
fiG|2202568.10.peG.27	8
fiG|2202568.10.peG.18	7
fiG|2202568.10.peG.45	7
fiG|2202568.10.peG.57	7
fiG|2202568.10.peG.4	6
fiG|2202568.10.peG.6	6
fiG|2202568.10.peG.8	6
fiG|2202568.10.peG.15	6
fiG|2202568.10.peG.16	6
fiG|2202568.10.peG.17	6
fiG|2202568.10.peG.22	6
fiG|2202568.10.peG.24	6
fiG|2202568.10.peG.29	6
fiG|2202568.10.peG.52	6
fiG|2202568.10.peG.56	6
fiG|2202568.10.peG.59	6
fiG|2202568.10.peG.63	6
fiG|2202568.10.peG.65	6
fiG|2202568.10.peG.72	6
fiG|2202568.10.peG.77	6
fiG|2202568.10.peG.78	6
fiG|2202568.10.peG.5	5
fiG|2202568.10.peG.10	5
fiG|2202568.10.peG.13	5
fiG|2202568.10.peG.19	5

Supplementary Table S5. Phage Depolymerase Finder (PhageDPO) results

ID	ANN7185 DPO Prediction (%)
fiG|2202568.10.peG.49	0
fiG|2202568.10.peG.48	0
fiG|2202568.10.peG.47	0
fiG|2202568.10.peG.45	0
fiG|2202568.10.peG.43	0
fiG|2202568.10.peG.41	0
fiG|2202568.10.peG.40	0
fiG|2202568.10.peG.4	0
fiG|2202568.10.peG.39	0
fiG|2202568.10.peG.38	0
fiG|2202568.10.peG.37	0
fiG|2202568.10.peG.36	0
fiG|2202568.10.peG.35	0
fiG|2202568.10.peG.34	0
fiG|2202568.10.peG.33	0
fiG|2202568.10.peG.31	0
fiG|2202568.10.peG.30	0
fiG|2202568.10.peG.3	0
fiG|2202568.10.peG.29	0
fiG|2202568.10.peG.28	0
fiG|2202568.10.peG.27	0
fiG|2202568.10.peG.26	0
fiG|2202568.10.peG.25	0
fiG|2202568.10.peG.24	0
fiG|2202568.10.peG.23	0
fiG|2202568.10.peG.22	0
fiG|2202568.10.peG.21	0
fiG|2202568.10.peG.20	0
fiG|2202568.10.peG.2	0
fiG|2202568.10.peG.19	0
fiG|2202568.10.peG.18	0
fiG|2202568.10.peG.17	0
fiG|2202568.10.peG.16	0
fiG|2202568.10.peG.15	0
fiG|2202568.10.peG.14	0
fiG|2202568.10.peG.13	0
fiG|2202568.10.peG.12	0
fiG|2202568.10.peG.11	0
fiG|2202568.10.peG.10	0
fiG|2202568.10.peG.1	0

ID	SVM4311 DPO Prediction (%)
fiG|2202568.10.peG.25	5
fiG|2202568.10.peG.37	5
fiG|2202568.10.peG.53	5
fiG|2202568.10.peG.62	5
fiG|2202568.10.peG.67	5
fiG|2202568.10.peG.73	5
fiG|2202568.10.peG.74	5
fiG|2202568.10.peG.82	5
fiG|2202568.10.peG.83	5
fiG|2202568.10.peG.9	4
fiG|2202568.10.peG.30	4
fiG|2202568.10.peG.33	4
fiG|2202568.10.peG.34	4
fiG|2202568.10.peG.35	4
fiG|2202568.10.peG.36	4
fiG|2202568.10.peG.49	4
fiG|2202568.10.peG.75	4
fiG|2202568.10.peG.76	4
fiG|2202568.10.peG.1	3
fiG|2202568.10.peG.7	3
fiG|2202568.10.peG.23	3
fiG|2202568.10.peG.26	3
fiG|2202568.10.peG.31	3
fiG|2202568.10.peG.39	3
fiG|2202568.10.peG.41	3
fiG|2202568.10.peG.14	2
fiG|2202568.10.peG.21	2
fiG|2202568.10.peG.28	2
fiG|2202568.10.peG.38	2
fiG|2202568.10.peG.40	2
fiG|2202568.10.peG.47	2
fiG|2202568.10.peG.48	2
fiG|2202568.10.peG.50	2
fiG|2202568.10.peG.51	2
fiG|2202568.10.peG.60	1
fiG|2202568.10.peG.64	1
fiG|2202568.10.peG.80	1
fiG|2202568.10.peG.81	1
fiG|2202568.10.peG.84	1
fiG|2202568.10.peG.79	0

Supplementary Table S6. BLASTn results of vB_Kpn_ZCKp20p whole genome

Description	Max Score	Total Score	Query Cover	E value	Per. ident	Acc. Len	Accession	Publication citation
Klebsiella phage ZCKP8	40293	76010	88%	0	98.26	48490	MZ440881.1	Fayez MS, Hakim TA, Agwa MM, Abdelmoteleb M, Aly RG, Montaser NN, Abdelsattar AS, Rezk N, El-Shibiny A. Topically Applied Bacteriophage to Control Multi-Drug Resistant Klebsiella pneumoniae Infected Wound in a Rat Model. Antibiotics (Basel). 2021 Aug 27;10(9):1048. doi: 10.3390/antibiotics10091048. PMID: 34572629; PMCID: PMC8470685.
Klebsiella phage 6991, complete genome	22472	57102	72%	0	96.83	46373	OL362277.1	Unpublished
Flyfo siphovirus Tbat1_6, complete genome	7463	41581	60%	0	92.32	48082	OL617041.1	Unpublished
Vibrio phage pYD38-A genomic sequence	7367	48378	71%	0	92.23	47552	JF974312.1	Unpublished
Aeromonas phage pIS4-A genomic sequence	7367	48042	71%	0	92.2	47624	NC_042037.1	Unpublished
Klebsiella phage BUCT610, complete genome	7203	44459	66%	0	89.8	46774	MZ318367.1	Pu M, Han P, Zhang G, Liu Y, Li Y, Li F, Li M, An X, Song L, Chen Y, Fan H, Tong Y. Characterization and Comparative Genomics Analysis of a New Bacteriophage BUCT610 against Klebsiella pneumoniae and Efficacy Assessment in Galleria mellonella Larvae. Int J Mol Sci. 2022 Jul 21;23(14):8040. doi: 10.3390/ijms23148040. PMID: 35887393; PMCID: PMC9321532.
Klebsiella phage YX3973, complete genome	7066	27304	46%	0	85.75	46907	NC_054652.1	Unpublished
Klebsiella phage ZX4, complete genome	7027	38187	62%	0	85.65	45424	NC_054654.1	Unpublished
Klebsiella phage BUCT541	7022	34498	59%	0	85.62	46100	MZ836210.1	Unpublished
Klebsiella phage vB_KpnS_MK54	6687	43281	61%	0	92.86	46218	MW119258.1	Lu B, Yao X, Han G, Luo Z, Zhang J, Yong K, Wang Y, Luo Y, Yang Z, Ren M, Cao S. Isolation of Klebsiella pneumoniae Phage vB_KpnS_MK54 and Pathological Assessment of Endolysin in the Treatment of Pneumonia Mice Model. Front Microbiol. 2022 Mar 21;13:854908. doi: 10.3389/fmicb.2022.854908. PMID: 35387089; PMCID: PMC8978833.
Klebsiella virus KpV2811, complete genome	6063	39695	63%	0	86.67	46391	NC_054653.1	Unpublished
Escherichia phage C1, complete genome	1786	4996	19%	0	76.21	46667	NC_054651.1	
Escherichia phage S192, partial genome	1786	4996	18%	0	76.2	46667	ON239132.1	
Shigella phage Sf11 SMD-2017, complete genome	1718	2360	9%	0	75.97	46454	NC_054636.1	
TPA: *Siphoviridae* sp. isolate ctYo41	1646	1778	6%	0	76.7	43934	BK021922.1	
TPA: *Siphoviridae* sp. isolate ctbcf4	1613	5673	18%	0	76.58	48602	BK051619.1	
TPA: *Siphoviridae* sp. isolate ct6rD49	1605	4672	14%	0	76.36	47586	BK048029.1	
TPA: Siphoviridae sp. isolate ctc1i3	1482	2349	9%	0	76.52	45865	BK048082.1	
Salmonella phage Akira, complete genome	1450	3068	10%	0	76.11	45367	NC_054647.1	
Salmonella phage LPST10, complete genome	1439	2873	9%	0	79.69	47657	NC_054645.1	
Mutant *Salmonella* phage seszw clone seszw_6t3, complete genome	1419	3318	11%	0	76.06	41854	OL964744.1	
Mutant *Salmonella* phage seszw clone seszw_6t2, complete genome	1419	3318	11%	0	76.06	42512	OL964743.1	
Mutant *Salmonella* phage seszw clone seszw_6t1, complete genome	1419	3318	11%	0	76.06	41794	OL964742.1	
Mutant *Salmonella* phage seszw clone seszw_2t2, complete genome	1419	3318	11%	0	76.06	43428	OL964752.1	
Mutant *Salmonella* phage seszw clone seszw_2t1, complete genome	1419	3318	11%	0	76.06	43528	OL964751.1	
Mutant *Salmonella* phage seszw clone seszw_2t3, complete genome	1419	3318	11%	0	76.06	43626	OL964734.1	
Mutant *Salmonella* phage seszw clone seszw_270L, complete genome	1419	3312	11%	0	76.06	37892	MZ375347.1	
Salmonella phage seszw clone seszw_wt, complete genome	1419	3324	11%	0	76.06	45807	MZ375232.1	
Mutant *Salmonella* phage seszw clone seszw_300L.2, complete genome	1419	3318	11%	0	76.06	36655	MZ375355.1	
Mutant *Salmonella* phage seszw clone seszw_300S, complete genome	1419	3318	11%	0	76.06	36991	MZ375354.1	
Mutant *Salmonella* phage seszw clone seszw_300L, complete genome	1419	3318	11%	0	76.06	37788	MZ375353.1	
Mutant *Salmonella* phage seszw clone seszw_290L, complete genome	1419	3312	11%	0	76.06	36536	MZ375351.1	
Mutant *Salmonella* phage seszw clone seszw_280L, complete genome	1419	3312	11%	0	76.06	36975	MZ375349.1	
Mutant *Salmonella* phage seszw clone seszw_270S, complete genome	1419	3318	11%	0	76.06	37845	MZ375348.1	
Mutant *Salmonella* phage seszw clone seszw_260S, complete genome	1419	3318	11%	0	76.06	37571	MZ375346.1	
Mutant *Salmonella* phage seszw clone seszw_260L, complete genome	1419	3318	11%	0	76.06	37385	MZ375345.1	
Mutant *Salmonella* phage seszw clone seszw_250S, complete genome	1419	3318	11%	0	76.06	38365	MZ375344.1	
Mutant *Salmonella* phage seszw clone seszw_250L, complete genome	1419	3307	11%	0	76.06	38024	MZ375343.1	
Mutant *Salmonella* phage seszw clone seszw_240S, complete genome	1419	3312	11%	0	76.06	37980	MZ375342.1	
Mutant *Salmonella* phage seszw clone seszw_240L, complete genome	1419	3324	11%	0	76.06	37969	MZ375341.1	
Mutant *Salmonella* phage seszw clone seszw_220S, complete genome	1419	3318	11%	0	76.06	37730	MZ375338.1	
Mutant *Salmonella* phage seszw clone seszw_220L, complete genome	1419	3318	11%	0	76.06	38358	MZ375337.1	
Mutant *Salmonella* phage seszw clone seszw_210L, complete genome	1419	3069	10%	0	76.06	38033	MZ375335.1	
Mutant *Salmonella* phage seszw clone seszw_200S, complete genome	1419	3312	11%	0	76.06	39171	MZ375334.1	
Mutant *Salmonella* phage seszw clone seszw_190S, complete genome	1419	3307	11%	0	76.06	39898	MZ375332.1	
Mutant *Salmonella* phage seszw clone seszw_190L, complete genome	1419	3318	11%	0	76.06	40874	MZ375331.1	
Mutant *Salmonella* phage seszw clone seszw_180S, complete genome	1419	3205	11%	0	76.06	37889	MZ375330.1	
Mutant *Salmonella* phage seszw clone seszw_180L, complete genome	1419	3318	11%	0	76.06	38968	MZ375329.1	
Mutant *Salmonella* phage seszw clone seszw_170L, complete genome	1419	3318	11%	0	76.06	39511	MZ375327.1	
Mutant *Salmonella* phage seszw clone seszw_160S, complete genome	1419	3318	11%	0	76.06	38984	MZ375326.1	
Mutant *Salmonella* phage seszw clone seszw_160L, complete genome	1419	3318	11%	0	76.06	37727	MZ375325.1	
Mutant *Salmonella* phage seszw clone seszw_150S, complete genome	1419	3318	11%	0	76.06	40462	MZ375324.1	

Supplementary Table S6. BLASTn results of vB_Kpn_ZCKp20p whole genome

Description	Max Score	Total Score	Query Cover	E value	Per. ident	Acc. Len	Accession	Publication citation
Mutant *Salmonella* phage seszw clone seszw_150L, complete genome	1419	3318	11%	0	76.06	40035	MZ375323.1	[redacted]
Mutant *Salmonella* phage seszw clone seszw_140S, complete genome	1419	3312	11%	0	76.06	39996	MZ375322.1	[redacted]
Mutant *Salmonella* phage seszw clone seszw_130S, complete genome	1419	3324	11%	0	76.06	45900	MZ375320.1	[redacted]
Mutant *Salmonella* phage seszw clone seszw_120S, complete genome	1419	3318	11%	0	76.06	40916	MZ375318.1	[redacted]
Mutant *Salmonella* phage seszw clone seszw_120L, complete genome	1419	3318	11%	0	76.06	40621	MZ375317.1	[redacted]
Mutant *Salmonella* phage seszw clone seszw_110S, complete genome	1419	3318	11%	0	76.06	40876	MZ375316.1	[redacted]
Mutant *Salmonella* phage seszw clone seszw_110L, complete genome	1419	3318	11%	0	76.06	40680	MZ375315.1	[redacted]
Mutant *Salmonella* phage seszw clone seszw_100S, complete genome	1419	3318	11%	0	76.06	42961	MZ375314.1	[redacted]
Mutant *Salmonella* phage seszw clone seszw_100L, complete genome	1419	3324	11%	0	76.06	45853	MZ375313.1	[redacted]
Mutant *Salmonella* phage seszw clone seszw_90S, complete genome	1419	3307	11%	0	76.06	41868	MZ375312.1	[redacted]
Mutant *Salmonella* phage seszw clone seszw_90L, complete genome	1419	3318	11%	0	76.06	41749	MZ375311.1	[redacted]
Mutant *Salmonella* phage seszw clone seszw_80S, complete genome	1419	3318	11%	0	76.06	42007	MZ375310.1	[redacted]
Mutant *Salmonella* phage seszw clone seszw_80L, complete genome	1419	3318	11%	0	76.06	42032	MZ375309.1	[redacted]
Mutant *Salmonella* phage seszw clone seszw_70S, complete genome	1419	3318	11%	0	76.06	42032	MZ375308.1	[redacted]
Mutant *Salmonella* phage seszw clone seszw_70L, complete genome	1419	3213	11%	0	76.06	42912	MZ375307.1	[redacted]
Mutant *Salmonella* phage seszw clone seszw_60S, complete genome	1419	3318	11%	0	76.06	43917	MZ375306.1	[redacted]
Mutant *Salmonella* phage seszw clone seszw_60L, complete genome	1419	3205	11%	0	76.06	43337	MZ375305.1	[redacted]
Mutant *Salmonella* phage seszw clone seszw_50S, complete genome	1419	3324	11%	0	76.06	44906	MZ375304.1	[redacted]
Mutant *Salmonella* phage seszw clone seszw_50L, complete genome	1419	3324	11%	0	76.06	44462	MZ375303.1	[redacted]
Mutant *Salmonella* phage seszw clone seszw_40S, complete genome	1419	3220	11%	0	76.06	45155	MZ375302.1	[redacted]
Mutant *Salmonella* phage seszw clone seszw_40L, complete genome	1419	3324	11%	0	76.06	43557	MZ375301.1	[redacted]
Mutant *Salmonella* phage seszw clone seszw_30L, complete genome	1419	3324	11%	0	76.06	44704	MZ375299.1	[redacted]
Mutant *Salmonella* phage seszw clone seszw_20L, complete genome	1419	3324	11%	0	76.06	45558	MZ375297.1	[redacted]
Mutant *Salmonella* phage seszw clone seszw_230L, complete genome	1413	3312	11%	0	76.03	38108	MZ375339.1	[redacted]
TPA: *Siphoviridae* sp. isolate ctTyE9	1411	3576	10%	0	79.12	45433	BK033144.1	[redacted]
Klebsiella pneumoniae strain E16KP0268 chromosome, complete genome	1402	2551	5%	0	96.15	5280881	CP052268.1	[redacted]
Mutant *Salmonella* phage seszw clone seszw_200L, complete genome	1391	3178	9%	0	78.81	38195	MZ375333.1	[redacted]
Mutant *Salmonella* phage seszw clone seszw_20S, complete genome	1391	3296	10%	0	78.81	45533	MZ375298.1	[redacted]

Supplementary Table S7. ANIb results of vB_Kpn_ZCKp20p genome on JSpeciesWS

Genome	ANIb [%]	Aligned [%]	Aligned [bp]	Total [bp]
Klebsiella_phage_ZCKP8	96.73	86.77	42343	48797
Klebsiella_phage_6991	95.49	71.82	35044	48797
Klebsiella_phage_BUCT61	90.29	52.7	25714	48797
Klebsiella_phage_vB_KpnS_MK54	89.94	50.77	24776	48797
Flyfo_siphovirus_Tbat1_6	89.71	53.25	25984	48797
Aeromonas_phage_pIS4-A	88.37	60.1	29327	48797
Vibrio_phage_pYD38-A	88.35	60.13	29340	48797
Klebsiella_virus_KpV2811	87.5	55.91	27284	48797
Klebsiella_phage_ZX4	86.29	56.94	27785	48797
Klebsiella_phage_BUCT541	85.24	53.23	25976	48797
Klebsiella_phage_YX3973	84.67	48.2	23518	48797

Supplementary Table S8. PATRIC proteomic comparison results

vB_Kpn_ZCKp20p (Reference Genome)			ZCKP8 (Genome 1)					Phage_6991 (Genome 2)				
ref_genome_patric_id	Start	End	Hit	aa length	patric_id / locus tag	Identity	Coverage	Hit	aa length	patric_id / locus tag	Identity	Coverage
fig|2202568.10.peg.1	1	186	bi (<->)	61	lcl|MZ440881.1_prot_QYW02876.1_1	1	0.984	bi (<->)	66	lcl|OL362277.1_prot_URY99605.1_71	0.898	0.879
fig|2202568.10.peg.2	247	525	bi (<->)	92	lcl|MZ440881.1_prot_QYW02877.1_2	1	0.989	bi (<->)	92	lcl|OL362277.1_prot_URY99606.1_72	1	0.989
fig|2202568.10.peg.3	586	969	bi (<->)	127	lcl|MZ440881.1_prot_QYW02878.1_3	1	0.992	bi (<->)	127	lcl|OL362277.1_prot_URY99607.1_73	1	0.992
fig|2202568.10.peg.4	1030	1314	bi (<->)	94	lcl|MZ440881.1_prot_QYW02879.1_4	1	0.989	bi (<->)	94	lcl|OL362277.1_prot_URY99608.1_74	1	0.989
fig|2202568.10.peg.5	1375	1734	bi (<->)	167	lcl|MZ440881.1_prot_QYW02880.1_5	1	0.707	bi (<->)	119	lcl|OL362277.1_prot_URY99609.1_75	1	0.992
fig|2202568.10.peg.6	1795	1923	bi (<->)	42	lcl|MZ440881.1_prot_QYW02881.1_6	1	0.976	bi (<->)	42	lcl|OL362277.1_prot_URY99610.1_76	1	0.976
fig|2202568.10.peg.7	1984	2835	bi (<->)	302	lcl|MZ440881.1_prot_QYW02882.1_7	1	0.934	bi (<->)	283	lcl|OL362277.1_prot_URY99611.1_77	1	0.996
fig|2202568.10.peg.8	2896	2988						bi (<->)	30	lcl|OL362277.1_prot_URY99612.1_78	0.8	0.967
fig|2202568.10.peg.9	3049	3207	bi (<->)	52	lcl|MZ440881.1_prot_QYW02883.1_8	1	0.981	bi (<->)	52	lcl|OL362277.1_prot_URY99613.1_79	1	0.981
fig|2202568.10.peg.10	3268	3927	bi (<->)	139	lcl|MZ440881.1_prot_QYW02885.1_10	0.352	0.827	bi (<->)	149	lcl|OL362277.1_prot_URY99535.1_1	0.993	0.993
fig|2202568.10.peg.12	3988	5412	bi (<->)	480	lcl|MZ440881.1_prot_QYW02886.1_11	0.97	0.985	bi (<->)	474	lcl|OL362277.1_prot_URY99536.1_2	0.979	0.998
fig|2202568.10.peg.13	5473	5763	bi (<->)	96	lcl|MZ440881.1_prot_QYW02928.1_12	0.979	0.99	bi (<->)	96	lcl|OL362277.1_prot_URY99538.1_4	0.979	0.99
fig|2202568.10.peg.14	5824	6063	bi (<->)	79	lcl|MZ440881.1_prot_QYW02887.1_13	0.975	0.987	bi (<->)	79	lcl|OL362277.1_prot_URY99539.1_5	1	0.987
fig|2202568.10.peg.15	6124	6318	bi (<->)	62	lcl|MZ440881.1_prot_QYW02889.1_15	0.609	0.984	bi (<->)	64	lcl|OL362277.1_prot_URY99540.1_6	1	0.984
fig|2202568.10.peg.16	6379	6567						bi (<->)	62	lcl|OL362277.1_prot_URY99541.1_7	1	0.984
fig|2202568.10.peg.17	6628	6828	bi (<->)	66	lcl|MZ440881.1_prot_QYW02891.1_17	0.848	0.985	bi (<->)	67	lcl|OL362277.1_prot_URY99542.1_8	0.955	0.97
fig|2202568.10.peg.18	6889	7164										
fig|2202568.10.peg.19	7225	7344										
fig|2202568.10.peg.20	7405	8859	bi (<->)	464	lcl|MZ440881.1_prot_QYW02893.1_19	0.642	0.998	bi (<->)	483	lcl|OL362277.1_prot_URY99544.1_10	0.936	0.992
fig|2202568.10.peg.21	8920	9864	bi (<->)	315	lcl|MZ440881.1_prot_QYW02895.1_21	0.724	0.997	bi (<->)	314	lcl|OL362277.1_prot_URY99545.1_11	0.946	0.997
fig|2202568.10.peg.22	9925	10047	bi (<->)	40	lcl|MZ440881.1_prot_QYW02929.1_22	1	0.975					
fig|2202568.10.peg.23	10108	10971	bi (<->)	287	lcl|MZ440881.1_prot_QYW02930.1_23	1	0.997	bi (<->)	302	lcl|OL362277.1_prot_URY99547.1_13	0.476	0.997
fig|2202568.10.peg.24	11032	11163	bi (<->)	43	lcl|MZ440881.1_prot_QYW02931.1_24	1	0.977	bi (<->)	43	lcl|OL362277.1_prot_URY99548.1_14	0.977	0.977
fig|2202568.10.peg.25	11224	11514	bi (<->)	96	lcl|MZ440881.1_prot_QYW02932.1_25	1	0.99	bi (<->)	96	lcl|OL362277.1_prot_URY99549.1_15	0.979	0.99
fig|2202568.10.peg.26	11575	12012	bi (<->)	145	lcl|MZ440881.1_prot_QYW02896.1_26	0.993	0.993	bi (<->)	145	lcl|OL362277.1_prot_URY99550.1_16	0.986	0.993
fig|2202568.10.peg.27	12073	12318	bi (<->)	81	lcl|MZ440881.1_prot_QYW02897.1_27	0.778	0.988	bi (<->)	79	lcl|OL362277.1_prot_URY99551.1_17	0.747	0.987
fig|2202568.10.peg.28	12379	12873	bi (<->)	164	lcl|MZ440881.1_prot_QYW02898.1_28	0.896	0.988	bi (<->)	164	lcl|OL362277.1_prot_URY99552.1_18	0.859	0.988
fig|2202568.10.peg.29	12934	13077										
fig|2202568.10.peg.30	13138	13377										
fig|2202568.10.peg.31	13438	13650	bi (<->)	43	lcl|MZ440881.1_prot_QYW02934.1_30	0.816	0.86	bi (<->)	70	lcl|OL362277.1_prot_URY99553.1_19	0.928	0.971
fig|2202568.10.peg.32	13711	14097	bi (<->)	129	lcl|MZ440881.1_prot_QYW02936.1_32	0.992	0.984	bi (<->)	129	lcl|OL362277.1_prot_URY99554.1_20	1	0.984
fig|2202568.10.peg.33	14158	14352						bi (<->)	64	lcl|OL362277.1_prot_URY99555.1_21	1	0.984
fig|2202568.10.peg.34	14413	14613	bi (<->)	66	lcl|MZ440881.1_prot_QYW02937.1_33	1	0.985	bi (<->)	66	lcl|OL362277.1_prot_URY99556.1_22	1	0.985
fig|2202568.10.peg.35	14674	14889	bi (<->)	71	lcl|MZ440881.1_prot_QYW02899.1_34	1	0.986	bi (<->)	71	lcl|OL362277.1_prot_URY99557.1_23	1	0.986
fig|2202568.10.peg.36	14950	15141	bi (<->)	63	lcl|MZ440881.1_prot_QYW02900.1_35	1	0.984	bi (<->)	63	lcl|OL362277.1_prot_URY99558.1_24	1	0.984
fig|2202568.10.peg.37	15202	15426	bi (<->)	74	lcl|MZ440881.1_prot_QYW02901.1_36	1	0.986	bi (<->)	74	lcl|OL362277.1_prot_URY99559.1_25	1	0.986
fig|2202568.10.peg.38	15487	15723	bi (<->)	78	lcl|MZ440881.1_prot_QYW02902.1_37	0.987	0.987	bi (<->)	78	lcl|OL362277.1_prot_URY99560.1_26	0.987	0.987
fig|2202568.10.peg.39	15784	16254	bi (<->)	168	lcl|MZ440881.1_prot_QYW02903.1_38	1	0.923	bi (<->)	156	lcl|OL362277.1_prot_URY99561.1_27	1	0.994
fig|2202568.10.peg.40	16315	16605	bi (<->)	96	lcl|MZ440881.1_prot_QYW02904.1_39	1	0.99	bi (<->)	96	lcl|OL362277.1_prot_URY99562.1_28	1	0.99
fig|2202568.10.peg.41	16666	17010	bi (<->)	114	lcl|MZ440881.1_prot_QYW02905.1_40	1	0.991	bi (<->)	114	lcl|OL362277.1_prot_URY99563.1_29	1	0.991
fig|2202568.10.peg.42	17071	18471	bi (<->)	466	lcl|MZ440881.1_prot_QYW02907.1_42	1	0.998	bi (<->)	466	lcl|OL362277.1_prot_URY99564.1_30	1	0.998
fig|2202568.10.peg.43	18532	18996	bi (<->)	154	lcl|MZ440881.1_prot_QYW02908.1_43	1	0.994	bi (<->)	154	lcl|OL362277.1_prot_URY99565.1_31	1	0.994
fig|2202568.10.peg.44	19057	20145	bi (<->)	362	lcl|MZ440881.1_prot_QYW02909.1_44	1	0.997	bi (<->)	362	lcl|OL362277.1_prot_URY99566.1_32	1	0.997
fig|2202568.10.peg.45	20206	20400	bi (<->)	64	lcl|MZ440881.1_prot_QYW02910.1_45	1	0.984	bi (<->)	64	lcl|OL362277.1_prot_URY99567.1_33	1	0.984
fig|2202568.10.peg.46	20461	21279	bi (<->)	276	lcl|MZ440881.1_prot_QYW02911.1_46	1	0.982	bi (<->)	272	lcl|OL362277.1_prot_URY99568.1_34	1	0.996
fig|2202568.10.peg.47	21340	21522	bi (<->)	60	lcl|MZ440881.1_prot_QYW02938.1_47	1	0.983	bi (<->)	60	lcl|OL362277.1_prot_URY99569.1_35	1	0.983
fig|2202568.10.peg.48	21583	22119	bi (<->)	192	lcl|MZ440881.1_prot_QYW02939.1_48	1	0.922	bi (<->)	178	lcl|OL362277.1_prot_URY99570.1_36	1	0.994
fig|2202568.10.peg.49	22180	22356	bi (<->)	58	lcl|MZ440881.1_prot_QYW02940.1_49	0.966	0.983	bi (<->)	58	lcl|OL362277.1_prot_URY99571.1_37	0.983	0.983
fig|2202568.10.peg.50	22417	22764	bi (<->)	115	lcl|MZ440881.1_prot_QYW02941.1_50	1	0.991	bi (<->)	115	lcl|OL362277.1_prot_URY99572.1_38	1	0.991
fig|2202568.10.peg.51	22825	23640	bi (<->)	283	lcl|MZ440881.1_prot_QYW02942.1_51	1	0.954	bi (<->)	271	lcl|OL362277.1_prot_URY99573.1_39	1	0.996
fig|2202568.10.peg.52	23701	23817	bi (<->)	38	lcl|MZ440881.1_prot_QYW02943.1_52	1	0.974	bi (<->)	38	lcl|OL362277.1_prot_URY99574.1_40	1	0.974
fig|2202568.10.peg.53	23878	24174	bi (<->)	102	lcl|MZ440881.1_prot_QYW02944.1_53	1	0.951	bi (<->)	98	lcl|OL362277.1_prot_URY99575.1_41	1	0.99
fig|2202568.10.peg.54	24235	24522	bi (<->)	95	lcl|MZ440881.1_prot_QYW02912.1_54	1	0.989	bi (<->)	95	lcl|OL362277.1_prot_URY99576.1_42	0.937	0.989
fig|2202568.10.peg.55	24583	24975	bi (<->)	153	lcl|MZ440881.1_prot_QYW02913.1_55	1	0.843	bi (<->)	130	lcl|OL362277.1_prot_URY99577.1_43	0.869	0.992
fig|2202568.10.peg.56	25036	25170	bi (<->)	44	lcl|MZ440881.1_prot_QYW02914.1_56	1	0.977	bi (<->)	41	lcl|OL362277.1_prot_URY99578.1_44	0.488	0.976
fig|2202568.10.peg.57	25231	25575	bi (<->)	114	lcl|MZ440881.1_prot_QYW02915.1_57	1	0.991	bi (<->)	114	lcl|OL362277.1_prot_URY99579.1_45	0.877	0.991
fig|2202568.10.peg.58	25636	26052	bi (<->)	138	lcl|MZ440881.1_prot_QYW02916.1_58	1	0.986	bi (<->)	138	lcl|OL362277.1_prot_URY99580.1_46	0.956	0.986
fig|2202568.10.peg.59	26113	27243										
fig|2202568.10.peg.60	27304	27687	bi (<->)	127	lcl|MZ440881.1_prot_QYW02917.1_59	0.969	0.992	bi (<->)	127	lcl|OL362277.1_prot_URY99581.1_47	0.913	0.992
fig|2202568.10.peg.61	27748	28500	bi (<->)	250	lcl|MZ440881.1_prot_QYW02919.1_62	1	0.996	bi (<->)	250	lcl|OL362277.1_prot_URY99583.1_49	1	0.996
fig|2202568.10.peg.62	28561	29229	bi (<->)	222	lcl|MZ440881.1_prot_QYW02946.1_63	1	0.995	bi (<->)	217	lcl|OL362277.1_prot_URY99584.1_50	1	0.995
fig|2202568.10.peg.63	29290	29541	bi (<->)	83	lcl|MZ440881.1_prot_QYW02947.1_64	1	0.988	bi (<->)	83	lcl|OL362277.1_prot_URY99585.1_51	1	0.988
fig|2202568.10.peg.64	29602	30087	bi (<->)	161	lcl|MZ440881.1_prot_QYW02948.1_65	1	0.994	bi (<->)	161	lcl|OL362277.1_prot_URY99586.1_52	1	0.994
fig|2202568.10.peg.65	30148	30282	bi (<->)	49	lcl|MZ440881.1_prot_QYW02949.1_66	1	0.878	bi (<->)	44	lcl|OL362277.1_prot_URY99587.1_53	1	0.977
fig|2202568.10.peg.66	30343	31050	bi (<->)	235	lcl|MZ440881.1_prot_QYW02920.1_67	1	0.996	bi (<->)	235	lcl|OL362277.1_prot_URY99588.1_54	1	0.996
fig|2202568.10.peg.67	31111	34404	bi (<->)	1097	lcl|MZ440881.1_prot_QYW02921.1_68	0.957	0.999	bi (<->)	1097	lcl|OL362277.1_prot_URY99589.1_55	0.954	0.999
fig|2202568.10.peg.68	34465	34935	bi (<->)	156	lcl|MZ440881.1_prot_QYW02922.1_69	1	0.994	bi (<->)	156	lcl|OL362277.1_prot_URY99590.1_56	1	0.994
fig|2202568.10.peg.69	34996	35466	bi (<->)	156	lcl|MZ440881.1_prot_QYW02923.1_70	1	0.994	bi (<->)	156	lcl|OL362277.1_prot_URY99591.1_57	1	0.994
fig|2202568.10.peg.70	35527	38007	bi (<->)	826	lcl|MZ440881.1_prot_QYW02925.1_72	0.989	0.999	bi (<->)	818	lcl|OL362277.1_prot_URY99593.1_59	0.99	0.999
fig|2202568.10.peg.71	38068	40551	bi (<->)	830	lcl|MZ440881.1_prot_QYW02926.1_73	1	0.995	bi (<->)	827	lcl|OL362277.1_prot_URY99594.1_60	0.996	0.999

frontiers

Supplementary Table S8. PATRIC proteomic comparison results

vB_Kpn_ZCKp20p (Reference Genome)			ZCKP8 (Genome 1)					Phage_6991 (Genome 2)				
ref_genome_patric_id	Start	End	Hit	aa length	patric_id / locus tag	Identity	Coverage	Hit	aa length	patric_id / locus tag	Identity	Coverage
fig|2202568.10.peg.72	40612	40806										
fig|2202568.10.peg.73	40867	41370	bi (<->)	167	lcl|MZ440881.1_prot_QYW02950.1_74	1	0.994					
fig|2202568.10.peg.74	41431	42468	bi (<->)	345	lcl|MZ440881.1_prot_QYW02951.1_75	1	0.997					
fig|2202568.10.peg.75	42529	42804										
fig|2202568.10.peg.76	42865	43821	bi (<->)	318	lcl|MZ440881.1_prot_QYW02952.1_76	1	0.997					
fig|2202568.10.peg.77	43882	44325	bi (<->)	147	lcl|MZ440881.1_prot_QYW02953.1_77	1	0.993					
fig|2202568.10.peg.78	44386	44802	bi (<->)	138	lcl|MZ440881.1_prot_QYW02954.1_78	0.986	0.993	bi (<->)	152	lcl|OL362277.1_prot_URY99595.1_61	0.316	0.73
fig|2202568.10.peg.79	44863	46770	bi (<->)	635	lcl|MZ440881.1_prot_QYW02955.1_79	1	0.998	bi (<->)	620	lcl|OL362277.1_prot_URY99596.1_62	0.264	0.811
fig|2202568.10.peg.80	46831	47604	bi (<->)	257	lcl|MZ440881.1_prot_QYW02956.1_80	0.996	0.996					
fig|2202568.10.peg.81	47665	48558	bi (<->)	317	lcl|MZ440881.1_prot_QYW02927.1_81	1	0.934					
fig|2202568.10.peg.82	48619	48852	bi (<->)	77	lcl|MZ440881.1_prot_QYW02957.1_82	0.987	0.987					
fig|2202568.10.peg.83	48913	50466	bi (<->)	518	lcl|MZ440881.1_prot_QYW02958.1_83	1	0.996					
fig|2202568.10.peg.84	50527	50760	bi (<->)	78	lcl|MZ440881.1_prot_QYW02959.1_84	1	0.974					

frontiers

Supplementary Table S9. List of phages have the highest normalized tBLASTx scores (S_G) to vB_Kpn_ZCKp20p, calculated by ViPTree

NCBI Accession #	Phage Name	Genome length	Virus family / Morphotype	Host group	S_G to OP373729.1
OP373729.1	user_virus1: Klebsiella phage vB_Kpn_ZCKp20p	48797	Siphovirus	Gammaproteobacteria	1
MZ440881.1	user_virus2: Klebsiella phage ZCKP8	48490	Siphovirus	Gammaproteobacteria	0.9198
OL362277.1	user_virus3: Klebsiella phage 6991	46373	Siphovirus	Gammaproteobacteria	0.7499
NC_021534	Vibrio phage pYD38-A	47552	-	Gammaproteobacteria	0.6756
NC_042037	Aeromonas phage pIS4-A	47624	-	Gammaproteobacteria	0.6735
NC_054653	Klebsiella virus KpV2811	46391	Siphoviridae	Gammaproteobacteria	0.5959
NC_054654	Klebsiella phage vB_KpnS_ZX4	45424	Siphoviridae	Gammaproteobacteria	0.5953
NC_054652	Klebsiella phage YX3973	46907	Siphoviridae	Gammaproteobacteria	0.4602
NC_054650	Shigella phage DS8	44605	-	Gammaproteobacteria	0.4025
NC_054649	Escherichia phage vB_EcoS_swi2	47611	-	Gammaproteobacteria	0.3978
NC_054648	Salmonella phage vB_Se_STGO-35-1	47483	-	Gammaproteobacteria	0.3976
NC_031924	Salmonella phage IME207	47564	-	Gammaproteobacteria	0.3975
NC_054651	Escherichia phage C1	46667	-	Gammaproteobacteria	0.3887
NC_054639	Salmonella phage Skate	47393	-	Gammaproteobacteria	0.3822
NC_010495	Salmonella phage Vi II-E1	45051	-	Gammaproteobacteria	0.378
NC_054644	Salmonella virus VSt472	46905	-	Gammaproteobacteria	0.3738
NC_054643	Salmonella phage SeSz-2	45049	-	Gammaproteobacteria	0.3736
NC_054641	Salmonella virus KFS-SE2	48608	-	Gammaproteobacteria	0.3662
NC_054640	Salmonella phage Segz_1	48285	-	Gammaproteobacteria	0.3648
NC_054645	Salmonella phage LPST10	47657	-	Gammaproteobacteria	0.3585
NC_054646	Salmonella phage VB_StyS_BS5	47604	-	Gammaproteobacteria	0.3584
NC_054647	Salmonella phage Akira	45367	-	Gammaproteobacteria	0.3555
NC_054642	Salmonella phage Seszw_1	45881	-	Gammaproteobacteria	0.3367
NC_031918	Salmonella phage 64795_sal3	45342	-	Gammaproteobacteria	0.3325
NC_054638	Salmonella phage vB_SenS_SB28	45126	-	Gammaproteobacteria	0.3246
NC_054636	Shigella phage Sf11 SMD-2017	46454	-	Gammaproteobacteria	0.3033
NC_054637	Escherichia phage vB_EcoS Sa179lw	46833	-	Gammaproteobacteria	0.2738
NC_048198	Erwinia phage vB_EhrS_59	47116	-	Gammaproteobacteria	0.1205
NC_019927	Cronobacter phage ENT47670	47611	Siphoviridae	Gammaproteobacteria	0.1038
NC_048197	Erwinia phage vB_EhrS_49	46835	-	Gammaproteobacteria	0.0686
NC_049845	Klebsiella phage PhiKpNIH-2	49477	Drexlerviridae	Gammaproteobacteria	0.0602
NC_049847	Klebsiella phage Sin4	49916	Drexlerviridae	Gammaproteobacteria	0.0579
NC_049848	Klebsiella phage KP1801	49835	Drexlerviridae	Gammaproteobacteria	0.0573
NC_028743	Mannheimia phage vB_MhS_587AP2	48594	Siphoviridae	Gammaproteobacteria	0.0562
NC_028853	Mannheimia phage vB_MhS_535AP2	50078	Siphoviridae	Gammaproteobacteria	0.0562
NC_028760	Klebsiella phage KLPN1	49037	Drexlerviridae	Gammaproteobacteria	0.056
NC_047850	Klebsiella phage MezzoGao	49807	Drexlerviridae	Gammaproteobacteria	0.0559
NC_047820	Escherichia phage vB_EcoS_ESCO41	50800	Drexlerviridae	Gammaproteobacteria	0.0546
NC_049839	Klebsiella phage Sweeny	50241	Drexlerviridae	Gammaproteobacteria	0.0536
NC_049834	Klebsiella phage vB_KpnS_IMGroot	52866	Drexlerviridae	Gammaproteobacteria	0.0529
NC_048206	Escherichia phage vB_Eco_mar001J1	50343	Drexlerviridae	Gammaproteobacteria	0.0526
NC_048204	Escherichia phage vB_Eco_mar001J1	50343	Drexlerviridae	Gammaproteobacteria	0.0526
NC_049835	Klebsiella phage vB_KpnS_Domnhall	54438	Drexlerviridae	Gammaproteobacteria	0.0521
NC_042066	Tlsvirus SP126	51092	Drexlerviridae	Gammaproteobacteria	0.052
NC_048143	Klebsiella phage KpKT21phi1	49106	Drexlerviridae	Gammaproteobacteria	0.0518
NC_043469	Enterobacter phage F20	51543	Drexlerviridae	Gammaproteobacteria	0.0517
NC_031053	Klebsiella phage PKP126	50934	Drexlerviridae	Gammaproteobacteria	0.0517
NC_006949	Enterobacteria phage ES18	46900	Siphoviridae	Gammaproteobacteria	0.0516
NC_047907	Klebsiella phage GML-KpCol1	50249	Drexlerviridae	Gammaproteobacteria	0.0514
NC_049833	Klebsiella phage vB_KpnS_SegesCirculi	50713	Drexlerviridae	Gammaproteobacteria	0.0513
NC_041873	Escherichia phage JMPW2	50298	Drexlerviridae	Gammaproteobacteria	0.0512
NC_028786	Klebsiella phage 1513	49462	Drexlerviridae	Gammaproteobacteria	0.0508
NC_048043	Klebsiella phage NJS2	50132	Drexlerviridae	Gammaproteobacteria	0.0508
NC_049841	Klebsiella phage Skenny	49935	Drexlerviridae	Gammaproteobacteria	0.0508
NC_049842	Klebsiella phage vB_KpnS_15-38_KLPPOU149	49316	Drexlerviridae	Gammaproteobacteria	0.0508
NC_049836	Klebsiella phage vB_KpnS_Call	51487	Drexlerviridae	Gammaproteobacteria	0.0507

Supplementary Table S9. List of phages have the highest normalized tBLASTx scores (S_G) to vB_Kpn_ZCKp20p, calculated by ViPTree

NCBI Accession #	Phage Name	Genome length	Virus family / Morphotype	Host group	S_G to OP373729.1
NC_028774	Klebsiella phage Sushi	48754	Drexlerviridae	Gammaproteobacteria	0.0507
NC_047784	Klebsiella phage vB_KpnS_KpV522	51099	Drexlerviridae	Gammaproteobacteria	0.0506
NC_049840	Klebsiella phage vB_KpnS_FZ10	50381	Drexlerviridae	Gammaproteobacteria	0.0506
NC_047825	Klebsiella phage KOX1	50526	Drexlerviridae	Gammaproteobacteria	0.0505
NC_049837	Klebsiella phage vB_KpnS_Alina	51780	Drexlerviridae	Gammaproteobacteria	0.0505
NC_049814	Shigella phage JK16	51854	Drexlerviridae	Gammaproteobacteria	0.0504
NC_048162	Klebsiella phage GH-K3	49427	Drexlerviridae	Gammaproteobacteria	0.05
NC_047847	Shigella phage Sd1	48262	Drexlerviridae	Gammaproteobacteria	0.0498
NC_048126	Klebsiella phage TSK1	49861	Drexlerviridae	Gammaproteobacteria	0.0498
NC_055956	Klebsiella phage RAD2	49276	Drexlerviridae	Gammaproteobacteria	0.0497
NC_048024	Klebsiella phage NJS1	49292	Drexlerviridae	Gammaproteobacteria	0.0497
NC_047841	Klebsiella phage KPN N141	49090	Drexlerviridae	Gammaproteobacteria	0.0497
NC_049853	Escherichia phage Henu8	49890	Drexlerviridae	Gammaproteobacteria	0.0495
NC_049846	Klebsiella phage Shelby	49045	Drexlerviridae	Gammaproteobacteria	0.0494
NC_048042	Klebsiella phage TAH8	49344	Drexlerviridae	Gammaproteobacteria	0.0494
NC_048044	Klebsiella phage NJR15	49468	Drexlerviridae	Gammaproteobacteria	0.0493
NC_029099	Klebsiella phage KP36	49797	Drexlerviridae	Gammaproteobacteria	0.0491
NC_009540	Escherichia phage Tls	49902	Drexlerviridae	Gammaproteobacteria	0.0488
NC_047996	Escherichia phage Eco_BIFF	49372	Drexlerviridae	Gammaproteobacteria	0.0484
NC_021331	Shigella phage pSf-1	51821	Drexlerviridae	Gammaproteobacteria	0.0481
NC_049815	Escherichia phage tonn	51012	Drexlerviridae	Gammaproteobacteria	0.048
NC_047823	Citrobacter phage CF1 DK-2017	50339	Drexlerviridae	Gammaproteobacteria	0.0479
NC_019509	Cronobacter phage ESP2949-1	49116	Drexlerviridae	Gammaproteobacteria	0.0473
NC_048088	Cronobacter phage CS01	48195	Drexlerviridae	Gammaproteobacteria	0.0473
NC_048202	Escherichia phage vB_EcoS_swan01	50865	Drexlerviridae	Gammaproteobacteria	0.0473
NC_054893	Escherichia phage vB_EcoS_W011D	49847	Drexlerviridae	Gammaproteobacteria	0.0471
NC_023743	Escherichia phage phiEB49	47180	Drexlerviridae	Gammaproteobacteria	0.0465
NC_049819	Escherichia phage atuna	50732	Drexlerviridae	Gammaproteobacteria	0.0465
NC_049817	Escherichia phage tonnikala	51277	Drexlerviridae	Gammaproteobacteria	0.0463
NC_031050	Escherichia phage vB_EcoS_NBD2	51802	Drexlerviridae	Gammaproteobacteria	0.0462
NC_054892	Escherichia phage vB_EcoS_PHB17	48939	Drexlerviridae	Gammaproteobacteria	0.046
NC_047848	Shigella phage Sf12	47647	Drexlerviridae	Gammaproteobacteria	0.0458
NC_049821	Salmonella phage slyngel	51048	Drexlerviridae	Gammaproteobacteria	0.0458
NC_049822	Escherichia phage vB_EcoS_G29-2	51739	Drexlerviridae	Gammaproteobacteria	0.0458
NC_049824	Escherichia phage vojen	50709	Drexlerviridae	Gammaproteobacteria	0.0457
NC_048132	Escherichia phage vB_EcoS-95	50910	Drexlerviridae	Gammaproteobacteria	0.0457
NC_047893	Escherichia phage DTL	45814	Drexlerviridae	Gammaproteobacteria	0.0456
NC_047898	Salmonella phage YSP2	50316	Drexlerviridae	Gammaproteobacteria	0.0456
NC_049852	Escherichia phage egaa	51643	Drexlerviridae	Gammaproteobacteria	0.0455
NC_042122	Enterobacter phage Ec_L1	51894	Drexlerviridae	Gammaproteobacteria	0.0454
NC_049816	Escherichia phage tunus	51111	Drexlerviridae	Gammaproteobacteria	0.0453
NC_049825	Escherichia phage grams	49530	Drexlerviridae	Gammaproteobacteria	0.0452
NC_049829	Escherichia phage aalborv	46660	Drexlerviridae	Gammaproteobacteria	0.0451
NC_054894	Escherichia phage Henu7	49526	Drexlerviridae	Gammaproteobacteria	0.045

Supplementary Table S10. Intergenomic similarities of RefSeq phage genomes to vB_Kpn_ZCKp20p, calculated by VIRIDIC

Genome	Intergenomic Similarity%
**OP373729.1_Klebsiella_phage_vB_Kpn_ZCKp20p**	**100**
MZ440881.1_Klebsiella_phage_ZCKP8	90.563
OL362277.1_Klebsiella_phage_6991	72.83
NC_021534.1_Vibrio_phage_pYD38-A	69.987
NC_042037.1_Aeromonas_phage_pIS4-A	69.692
ON602738.1_Klebsiella_phage_VLCpiS13a	67.194
MZ318367.1_Klebsiella_phage_BUCT610	65.29
MW119258.1_Klebsiella_phage_vB_KpnS_MK54	63.136
ON602730.1_Klebsiella_phage_VLCpiS13f	62.906
NC_054654.1_Klebsiella_phage_ZX4	62.349
NC_054653.1_Klebsiella_virus_KpV2811	61.863
ON602759.1_Klebsiella_phage_VLCpiS13e	61.227
OL617041.1_Flyfo_siphovirus_Tbat1_6	59.321
MZ836210.1_Klebsiella_phage_BUCT541	58.067
ON602737.1_Klebsiella_phage_VLCpiS13c	55.541
ON602760.1_Klebsiella_phage_VLCpiS13d	54.09
NC_054652.1_Klebsiella_phage_YX3973	47.588
NC_054650.1_Shigella_phage_DS8	38.299
NC_054648.1_Salmonella_phage_vB_Se_STGO-35-1	37.569
NC_054651.1_Escherichia_phage_C1	37.312
NC_031924.1_Salmonella_phage_IME207	36.963
NC_054649.1_Escherichia_phage_vB_EcoS_swi2	34.781
NC_010495.1_Salmonella_phage_E1	33.952
NC_054639.1_Salmonella_phage_Skate	33.918
NC_054643.1_Salmonella_phage_SeSz-2	33.712
NC_054647.1_Salmonella_phage_Akira	33.612
NC_054641.1_Salmonella_virus_KFS-SE2	33.446
NC_054644.1_Salmonella_virus_VSt472	32.848
NC_054642.1_Salmonella_phage_Seszw_1	31.307
NC_054646.1_Salmonella_phage_VB_StyS_BS5	31.198
NC_054640.1_Salmonella_phage_Segz_1	30.694
NC_031918.1_Salmonella_phage_64795_sal3	30.236
NC_054645.1_Salmonella_phage_LPST10	30.204
NC_054638.1_Salmonella_phage_vB_SenS_SB28	28.372
NC_054636.1_Shigella_phage_Sf11_SMD-2017	27.699
NC_054637.1_Escherichia_coli_Phage_vB_EcoS-Sa179lw	26.024
NC_048198.1_Erwinia_phage_vB_EhrS_59	5.142
NC_019927.1_Cronobacter_phage_ENT47670	3.873
NC_048197.1_Erwinia_phage_vB_EhrS_49	2.51

frontiers

Supplementary Table S10. Intergenomic similarities of RefSeq phage genomes to vB_Kpn_ZCKp20p, calculated by VIRIDIC

Genome	Intergenomic Similarity%
NC_031048.1_Enterobacter_phage_Arya	1.905
NC_025430.1_Escherichia_phage_vB_EcoM-ep3	1.694
NC_048088.1_Cronobacter_phage_CS01	1.524
NC_019509.1_Cronobacter_phage_ESP2949-1	1.471
NC_048067.1_Escherichia_phage_C130_2	1.466
NC_006949.1_Enterobacteria_phage_ES18	1.402
NC_054897.1_Citrobacter_virus_HCF1	1.385
NC_009540.1_Enterobacteria_phage_TLS	1.295
NC_049836.1_Klebsiella_phage_vB_KpnS_Call	1.219
NC_027995.1_Escherichia_phage_vB_EcoM_ECO1230-10	1.197
NC_017981.1_Xanthomonas_phage_vB_XveM_DIBBI	1.193
NC_047825.1_Klebsiella_phage_KOX1	1.174
NC_049835.1_Klebsiella_phage_vB_KpnS_Domnhall	1.164
NC_054892.1_Escherichia_phage_vB_EcoS_PHB17	1.156
NC_048766.1_Pantoea_phage_vB_PagM_AAM37	1.146
NC_048767.1_Pantoea_phage_vB_PagM_PSKM	1.13
NC_047784.1_Klebsiella_phage_vB_KpnS_KpV522	1.117
NC_049853.1_Escherichia_phage_Henu8	1.102
NC_049833.1_Klebsiella_phage_vB_KpnS_SegesCirculi	1.079
NC_049837.1_Klebsiella_phage_vB_KpnS_Alina	1.076
NC_054898.1_Vibrio_virus_2019VC1	1.071
NC_048204.1_Escherichia_virus_vB_Eco_mar001J1_strain_vB_Eco_mar002J2	1.071
NC_048206.1_Escherichia_virus_vB_Eco_mar001J1	1.071
NC_054893.1_Escherichia_phage_vB_EcoS_W011D	1.071
NC_049819.1_Escherichia_phage_atuna	1.045
NC_048112.1_Escherichia_phage_Skarpretter	1.041
NC_047898.1_Salmonella_phage_YSP2	1.037
NC_028743.1_Mannheimia_phage_vB_MhS_587AP2	1.021
NC_028853.1_Mannheimia_phage_vB_MhS_535AP2	1.005
NC_049828.1_Escherichia_phage_haarsle	1.003
NC_049825.1_Escherichia_phage_grams	0.995
NC_021331.1_Shigella_phage_pSf-1	0.986
NC_049826.1_Escherichia_phage_aaroes	0.936
NC_049847.1_Klebsiella_phage_Sin4	0.933
NC_049848.1_Klebsiella_phage_KP1801	0.929
NC_049815.1_Escherichia_phage_tonn	0.922
NC_049817.1_Escherichia_phage_tonnikala	0.917
NC_049824.1_Escherichia_phage_vojen	0.915
NC_048178.1_Escherichia_phage_Sortsne	0.908
NC_049830.1_Escherichia_phage_PGN590	0.904
NC_049827.1_Escherichia_phage_damhaus	0.888

Supplementary Table S10. Intergenomic similarities of RefSeq phage genomes to vB_Kpn_ZCKp20p, calculated by VIRIDIC

Genome	Intergenomic Similarity%
NC_028760.1_Klebsiella_phage_KLPN1	0.885
NC_047820.1_Escherichia_phage_vB_EcoS_ESCO41	0.884
NC_047938.1_Escherichia_phage_SECphi27	0.877
NC_048132.1_Escherichia_phage_vB_EcoS-95	0.859
NC_041926.1_Escherichia_phage_vB_EcoM_ECOO78	0.846
NC_031050.1_Enterobacteria_phage_vB_EcoS_NBD2	0.845
NC_047985.1_Escherichia_phage_LL5	0.836
NC_005857.1_Klebsiella_phage_phiKO2	0.829
NC_047996.1_Escherichia_phage_Eco_BIFF	0.827
NC_048113.1_Salmonella_phage_Lumpael	0.823
NC_049834.1_Klebsiella_phage_vB_KpnS_IMGroot	0.816
NC_054894.1_Escherichia_phage_Henu7	0.816
NC_031026.1_Salmonella_phage_phSE-2	0.809
NC_047947.1_Salmonella_phage_vB_SenS_PHB07	0.801
NC_048202.1_Escherichia_phage_vB_Eco_swan01	0.8
NC_042122.1_Enterobacter_phage_Ec_L1	0.793
NC_048162.1_Klebsiella_phage_GH-K3	0.792
NC_049852.1_Escherichia_phage_egaa	0.787
NC_049829.1_Escherichia_phage_aalborv	0.784
NC_047823.1_Citrobacter_phage_CF1_DK-2017	0.779
NC_049816.1_Escherichia_phage_tunus	0.751
NC_049846.1_Klebsiella_phage_Shelby	0.75
NC_049814.1_Shigella_phage_JK16	0.749
NC_049838.1_Klebsiella_phage_KL	0.747
NC_054895.1_Escherichia_phage_vB_Eco_SLUR29	0.742
NC_054896.1_Escherichia_phage_tiwna	0.737
NC_049845.1_Klebsiella_phage_PhiKpNIH-2	0.736
NC_047850.1_Klebsiella_phage_MezzoGao	0.728
NC_049822.1_Escherichia_phage_vB_EcoS_G29-2	0.728
NC_049818.1_Escherichia_phage_tonijn	0.721
NC_049821.1_Salmonella_phage_slyngel	0.721
NC_027350.1_Citrobacter_phage_Stevie	0.718
NC_049820.1_Shigella_virus_2019SD1	0.717
NC_049823.1_Escherichia_phage_herni	0.716
NC_047862.1_Klebsiella_phage_vB_KpnS_IME279	0.71
NC_049844.1_Klebsiella_phage_13	0.701
NC_049840.1_Klebsiella_phage_vB_KpnS_FZ10	0.7
NC_047907.1_Klebsiella_virus_GML-KpCol1	0.697
NC_031053.1_Klebsiella_phage_PKP126	0.694
NC_041874.1_Escherichia_phage_JMPW1	0.693
NC_019719.1_Enterobacteria_phage_HK633	0.675

Supplementary Table S10. Intergenomic similarities of RefSeq phage genomes to vB_Kpn_ZCKp20p, calculated by VIRIDIC

Genome	Intergenomic Similarity%
NC_048042.1_Klebsiella_phage_TAH8	0.662
NC_029099.1_Klebsiella_phage_KP36	0.649
NC_004348.1_Enterobacteria_phage_ST64T	0.646
NC_028774.1_Klebsiella_phage_Sushi	0.636
NC_047841.1_Klebsiella_phage_KPN_N141	0.619
NC_049839.1_Klebsiella_phage_Sweeny	0.61
NC_026010.1_Shigella_phage_pSf-2	0.609
NC_002371.2_Salmonella_phage_P22	0.599
NC_047857.1_Klebsiella_phage_phiKpS2	0.599
NC_047847.1_Shigella_phage_Sd1	0.598
NC_031019.1_Enterobacteria_phage_UAB_Phi20	0.598
NC_047848.1_Shigella_phage_Sf12	0.595
NC_049842.1_Klebsiella_phage_vB_KpnS_15-38_KLPPOU149	0.589
NC_048043.1_Klebsiella_phage_NJS2	0.584
NC_055956.1_Klebsiella_phage_RAD2	0.569
NC_028696.2_Salmonella_phage_SEN22	0.561
NC_048065.1_Citrobacter_phage_Sazh	0.557
NC_019721.1_Enterobacterial_phage_mEp390	0.554
NC_047893.1_Escherichia_phage_DTL	0.546
NC_049841.1_Klebsiella_phage_Skenny	0.533
NC_013059.1_Salmonella_phage_c341	0.521
NC_018279.1_Salmonella_phage_vB_SosS_Oslo	0.519
NC_042346.1_Salmonella_virus_BTP1	0.515
NC_048024.1_Klebsiella_phage_NJS1	0.512
NC_047998.1_Shigella_phage_Sfin-1	0.512
NC_049813.1_Escherichia_phage_vB_EcoS-12210I	0.5
NC_042046.1_Escherichia_phage_C119	0.493
NC_047742.1_Enterobacteria_phage_IME_EC2	0.492
NC_025443.1_Salmonella_phage_9NA	0.492
NC_048143.1_Klebsiella_phage_KpKT21phi1	0.484
NC_049831.1_Shigella_virus_Sfin-3	0.472
NC_015295.1_Erwinia_phage_phiEt88	0.47
NC_047810.1_Escherichia_phage_vB_EcoS-IME253	0.467
NC_031946.1_Salmonella_Phage_103203_sal5	0.457
NC_023006.1_Pseudomonas_phage_PPpW-3	0.448
NC_048044.1_Klebsiella_phage_NJR15	0.436
NC_047959.1_Enterobacteria_phage_vB_EcoS_IME18	0.436
NC_047785.1_Shigella_phage_SH6	0.429
NC_049948.1_Escherichia_phage_Lambda_ev017	0.427
NC_021563.1_Serratia_phage_Eta	0.426
NC_019445.1_Escherichia_phage_TL-2011b	0.425

Supplementary Table S10. Intergenomic similarities of RefSeq phage genomes to vB_Kpn_ZCKp20p, calculated by VIRIDIC

Genome	Intergenomic Similarity%
NC_024789.1_Escherichia_phage_bV_EcoS_AKS96	0.419
NC_024793.1_Escherichia_phage_bV_EcoS_AHP42	0.414
NC_049926.1_Escherichia_phage_ECP1	0.41
NC_048150.1_Shigella_phage_vB_SsoS_008	0.391
NC_048126.1_Klebsiella_phage_TSK1	0.389
NC_007603.1_Enterobacteria_phage_RTP	0.386
NC_023743.1_Escherichia_phage_phiEB49	0.383
NC_042043.1_Escherichia_phage_SRT8	0.382
NC_048137.1_Pantoea_phage_vB_PagS_AAS23	0.378
NC_048208.1_Enterobacteria_phage_vB_EcoS_IME542	0.372
NC_024369.2_Vibrio_phage_X29	0.372
NC_011976.1_Salmonella_phage_epsilon34	0.368
NC_019404.1_Enterobacteria_phage_vB_EcoS_ACG-M12	0.36
NC_049832.1_Escherichia_phage_vB_EcoS-DELF2_DNA	0.358
NC_055836.1_Xanthomonas_phage_FoX2	0.355
NC_024210.1_Escherichia_phage_e4/1c	0.348
NC_015456.1_Shigella_phage_Shfl1	0.34
NC_047740.1_Enterobacteria_phage_phiJLA23	0.338
NC_041995.1_Shigella_phage_vB_SsoS-ISF002	0.338
NC_041873.1_Escherichia_phage_JMPW2	0.337
NC_019718.1_Enterobacteria_phage_vB_EcoS_Rogue1	0.334
NC_007291.1_Enterobacteria_phage_JK06	0.333
NC_024784.1_Escherichia_phage_bV_EcoS_AHS24	0.332
NC_054149.1_Pseudomonas_phage_Persinger	0.321
NC_048181.1_Raoultella_phage_RP180	0.32
NC_029066.1_Pseudomonas_phage_PS-1_DNA	0.316
NC_019710.1_Enterobacteria_phage_HK140	0.315
NC_019706.1_Enterobacteria_phage_mEp043_c-1	0.314
NC_028699.1_Salmonella_phage_SEN34	0.31
NC_019720.1_Enterobacterial_phage_mEp213	0.31
NC_030929.1_Pseudomonas_phage_JBD44	0.292
NC_019725.1_Escherichia_phage_ADB-2	0.286
NC_019545.1_Salmonella_phage_SPN3UB	0.285
NC_028786.1_Klebsiella_phage_1513	0.271
NC_042038.1_Enterobacteriophage_phiKP26	0.266
NC_021777.1_Salmonella_phage_Jersey	0.265
NC_047812.1_Escherichia_phage_vB_Ecos_CEB_EC3a	0.262
NC_054465.1_Aeromonas_phage_2_D05	0.262
NC_049941.1_Stx2-converting_phage_Stx2a_WGPS2_proviral_DNA	0.257
NC_016762.1_Pseudomonas_phage_phi297	0.255
NC_020205.1_Xanthomonas_citri_phage_CP2_DNA	0.251

Supplementary Table S10. Intergenomic similarities of RefSeq phage genomes to vB_Kpn_ZCKp20p, calculated by VIRIDIC

Genome	Intergenomic Similarity%
NC_005833.1_Enterobacteria_phage_T1	0.25
NC_019717.1_Enterobacteria_phage_HK225	0.249
NC_021190.1_Enterobacteria_phage_phi80	0.246
NC_030909.1_Pseudomonas_phage_YMC11/07/P54_PAE_BP	0.232
NC_047786.1_Salmonella_phage_vB_SenS_Sasha	0.21
NC_047828.1_Escherichia_phage_vB_EcoS_SH2	0.204
NC_047960.1_Enterobacteria_phage_vB_EcoS_IME347	0.204
NC_048130.1_Klebsiella_phage_KN4-1_DNA	0.189
NC_028819.1_Pseudoalteromonas_Phage_H103	0.189
NC_047908.1_Klebsiella_phage_SH-Kp_152410	0.187
NC_003356.1_Enterobacteria_phage_phiP27	0.183
NC_055838.1_Xanthomonas_phage_FoX5	0.179
NC_029019.1_Stenotrophomonas_phage_vB_SmaS-DLP_2	0.179
NC_019419.2_Enterobacteria_phage_JL1	0.178
NC_055835.1_Xanthomonas_phage_FoX1	0.177
NC_047863.1_Shigella_phage_vB_SflS-ISF001	0.169
NC_049466.2_Escherichia_phage_vB_EcoM_4HA13	0.168
NC_049944.1_Stx2-converting_phage_Stx2a_WGPS8_proviral_DNA	0.164
NC_019711.1_Enterobacteria_phage_HK629	0.162
NC_048875.1_Pantoea_phage_vB_PagM_SSEM1	0.162
NC_010116.1_Pseudomonas_phage_YuA	0.16
NC_049945.1_Stx2-converting_phage_Stx2a_WGPS6_proviral_DNA	0.16
NC_049923.1_Stx2-converting_phage_Stx2a_WGPS9_proviral_DNA	0.159
NC_028695.2_Enterobacter_phage_phiEap-2	0.159
NC_019716.1_Enterobacteria_phage_mEp460	0.146
NC_041897.1_Escherichia_phage_K1ind1	0.143
NC_019709.1_Enterobacteria_phage_mEpX1	0.142
NC_027994.1_Escherichia_phage_K1H	0.142
NC_027993.1_Escherichia_phage_K1G	0.139
NC_021776.1_Vibrio_phage_vpms1	0.138
NC_054466.1_Aeromonas_phage_4_D05	0.13
NC_052975.1_Klebsiella_phage_YMC16/01/N133_KPN_BP	0.125
NC_022752.1_Salmonella_phage_SETP13	0.12
NC_047821.1_Marinomonas_phage_CPP1m	0.116
NC_047836.1_Marinomonas_phage_CB5A	0.115
NC_048799.1_Escherichia_phage_Mangalitsa	0.107
NC_010106.1_Enterobacteria_phage_phiEcoM-GJ1	0.106
NC_019540.1_Salinivibrio_phage_CW02	0.106
NC_048845.1_Escherichia_phage_flopper	0.105
NC_031274.1_Pseudomonas_phage_O4	0.105
NC_047830.1_Escherichia_phage_ST32	0.104

Supplementary Table S10. Intergenomic similarities of RefSeq phage genomes to vB_Kpn_ZCKp20p, calculated by VIRIDIC

Genome	Intergenomic Similarity%
NC_025463.1_Shewanella_sp._phage_1/44	0.098
NC_011373.1_Pseudomonas_phage_PAJU2	0.096
NC_047978.1_Erwinia_phage_Faunus	0.093
NC_019767.1_Enterobacteria_phage_HK544	0.092
NC_012223.2_Enterobacteria_phage_SSL2009a	0.092
NC_004902.1_Xanthomonas_phage_Xp10	0.09
NC_007809.1_Pseudomonas_phage_M6	0.087
NC_020841.1_Psychrobacter_phage_pOW20-A	0.087
NC_027383.1_Escherichia_phage_YD-2008.s	0.084
NC_027980.1_Enterobacter_phage_phiKDA1	0.082
NC_031077.1_Enterobacter_phage_Tyrion	0.082
NC_042084.1_Escherichia_phage_VB_EcoS-Golestan	0.081
NC_055837.1_Xanthomonas_phage_FoX3	0.08
NC_019724.1_Enterobacteria_phage_HK578	0.08
NC_028901.1_Escherichia_phage_slur05	0.08
NC_031081.1_Escherichia_phage_Envy	0.079
NC_031113.1_Escherichia_phage_Gluttony	0.079
NC_041924.1_Acinetobacter_phage_WCHABP12	0.079
NC_041966.1_Acinetobacter_phage_WCHABP1	0.078
NC_026611.1_Edwardsiella_phage_GF-2_DNA,	0.076
NC_027330.1_Escherichia_phage_ECBP5	0.072
NC_019527.1_Aeromonas_phage_vB_AsaM-56	0.071
NC_028956.1_Mannheimia_phage_vB_MhS_1152AP2	0.071
NC_047791.1_Pectobacterium_phage_PP101	0.067
NC_016158.1_Escherichia_phage_HK639	0.065
NC_023865.1_Pectobacterium_phage_PM1	0.065
NC_023605.1_Vibrio_phage_PVA1	0.064
NC_019485.1_Enterobacter_phage_EcP1	0.063
NC_020846.1_Vibrio_phage_pYD21-A	0.063
NC_019518.1_Vibrio_phage_vB_VchM-138	0.062
NC_005069.1_Yersinia_phage_PY54	0.059
NC_049950.1_Escherichia_virus_Lambda_2H1	0.053
NC_053014.1_Pectobacterium_phage_MA11	0.052
NC_024146.1_Enterobacteria_phage_9g	0.051
NC_053015.1_Pectobacterium_phage_MA12	0.05
NC_016765.1_Pseudomonas_phage_PMG1	0.043
NC_002484.2_Bacteriophage_D3	0.042
NC_019722.1_Vibrio_phage_vB_VpaM_MAR	0
NC_004456.1_Vibrio_phage_VHML	0
NC_027981.1_Vibrio_phage_VP58.5	0
NC_007807.1_Pseudomonas_phage_119X	0

Supplementary Table S10. Intergenomic similarities of RefSeq phage genomes to vB_Kpn_ZCKp20p, calculated by VIRIDIC

Genome	Intergenomic Similarity%
NC_005884.1_Pseudomonas_phage_PaP2	0
NC_055711.1_Pseudomonas_phage_PaMx41	0
NC_054890.1_Pseudomonas_phage_vB_PsyP_3MF5	0
NC_023005.1_Pseudomonas_phage_PPpW-4_DNA	0
NC_047965.1_Pseudomonas_phage_22PfluR64PP	0
NC_047997.1_Pseudomonas_phage_PFP1	0
NC_015264.1_Pseudomonas_phage_phiIBB-PF7A	0
NC_021062.1_Pseudomonas_phage_Phi-S1	0
NC_047981.1_Pseudomonas_phage_PspYZU08	0
NC_048201.1_Pseudomonas_phage_17A	0
NC_047826.1_Pseudomonas_virus_WRT	0
NC_047827.1_Pseudomonas_virus_KNP	0
NC_024362.1_Pseudomonas_phage_phiPSA2	0
NC_047747.1_Pseudomonas_phage_phiPsa17	0
NC_047922.1_Pseudomonas_phage_Henninger	0
NC_028661.1_Pseudomonas_phage_PPPL-1	0
NC_048200.1_Pseudomonas_phage_shl2	0
NC_048025.1_Shigella_phage_SFPH2	0
NC_048071.1_Escherichia_phage_IMM-002	0
NC_031943.1_Escherichia_phage_vB_EcoP_GA2A	0
NC_047777.1_Escherichia_phage_ZG49	0
NC_048059.1_Dickeya_phage_Luksen	0
NC_048060.1_Dickeya_phage_Mysterion	0
NC_047961.1_Dickeya_phage_Dagda	0
NC_048057.1_Dickeya_phage_Katbat	0
NC_047797.2_Pectobacterium_phage_PP81	0
NC_047801.2_Pectobacterium_phage_PP47	0
NC_048052.1_Dickeya_phage_vB_DsoP_JA10	0
NC_047936.1_Yersinia_phage_fPS-53	0
NC_047937.1_Yersinia_phage_fPS-54-ocr	0
NC_028863.1_Escherichia_phage_P694	0
NC_028822.1_Escherichia_phage_P483	0
NC_048180.1_Shigella_phage_VB_Ship_A7	0
NC_047856.1_Enterobacter_phage_KNP3	0
NC_047780.1_Klebsiella_phage_vB_KpnP_PRA33	0
NC_047781.1_Klebsiella_phage_vB_KpnP_BIS33	0
NC_047782.1_Klebsiella_phage_vB_KpnP_IL33	0
NC_047772.1_Klebsiella_phage_vB_KpnP_KpV767	0
NC_013647.1_Klebsiella_phage_KP32	0
NC_028800.1_Klebsiella_phage_K5	0
NC_047969.1_Klebsiella_virus_KP32_isolate_194	0

frontiers

Supplementary Table S10. Intergenomic similarities of RefSeq phage genomes to vB_Kpn_ZCKp20p, calculated by VIRIDIC

Genome	Intergenomic Similarity%
NC_047771.1_Klebsiella_phage_vB_KpnP_KpV763	0
NC_047968.1_Klebsiella_virus_KP32_isolate_192	0
NC_047842.1_Klebsiella_phage_2044-307w	0
NC_048175.1_Klebsiella_phage_Pharr	0
NC_048131.1_Klebsiella_phage_KN3-1_DNA	0
NC_048138.1_Klebsiella_phage_Henu1	0
NC_011043.1_Klebsiella_phage_K11	0
NC_047798.1_Klebsiella_phage_K5-2	0
NC_047799.1_Klebsiella_phage_K5-4	0
NC_048129.1_Klebsiella_phage_KN1-1_DNA	0
NC_047971.1_Klebsiella_virus_KP32_isolate_196	0
NC_015719.1_Enterobacteria_phage_K30	0
NC_028977.1_Klebsiella_phage_vB_KpnP_KpV289	0
NC_047773.1_Klebsiella_phage_vB_KpnP_KpV766	0
NC_047761.1_Klebsiella_phage_vB_KpnP_IME205	0
NC_047970.1_Klebsiella_virus_KP32_isolate_195	0
NC_047980.1_Klebsiella_phage_SH-Kp_152234	0
NC_028688.1_Klebsiella_phage_vB_Kp1	0
NC_048114.1_Klebsiella_phage_kpssk3	0
NC_047886.1_Pectobacterium_phage_DU_PP_II	0
NC_047963.1_Pectobacterium_phage_Jarilo	0
NC_054460.1_Xanthomonas_phage_KPhi1	0
NC_054461.1_Xanthomonas_virus_phiXaf18	0
NC_007710.1_Xanthomonas_oryzae_phage_OP2_DNA	0
NC_054458.1_Xanthomonas_phage_XPP1	0
NC_054459.1_Xanthomonas_phage_XPV1	0
NC_005841.1_Enterobacteria_phage_ST104_DNA	0
NC_011802.1_Salmonella_enterica_bacteriophage_SE1	0
NC_014900.1_Salmonella_phage_ST160	0
NC_017985.1_Salmonella_phage_SPN9CC	0
NC_030919.1_Salmonella_phage_118970_sal4	0
NC_054464.1_Edwardsiella_phage_Edno5	0
NC_052979.1_Providencia_phage_Kokobel1	0
NC_052980.1_Proteus_phage_ASh-2020a	0
NC_052981.1_Proteus_phage_PM87	0
NC_052982.1_Proteus_phage_P16-2532	0
NC_028812.1_Proteus_phage_pPM_01	0
NC_052983.1_Escherichia_phage_E21	0
NC_052987.1_Klebsiella_phage_KPN_N98	0
NC_048670.1_Klebsiella_phage_KPN_N137	0
NC_052988.1_Klebsiella_phage_KPN_U2874	0

Supplementary Table S10. Intergenomic similarities of RefSeq phage genomes to vB_Kpn_ZCKp20p, calculated by VIRIDIC

Genome	Intergenomic Similarity%
NC_048732.1_Klebsiella_phage_Seifer	0
NC_048805.1_Klebsiella_phage_Soft	0
NC_018832.1_Providencia_phage_Redjac	0
NC_053008.1_Salmonella_phage_SAP012_DNA	0
NC_031930.1_Salmonella_phage_118970_sal1	0
NC_048683.1_Salmonella_phage_KFS-SE1	0
NC_021315.1_Enterobacteria_phage_Chi	0
NC_025442.1_Salmonella_phage_Chi	0
NC_048655.1_Salmonella_phage_BSPM4	0
NC_053000.1_Salmonella_phage_SeWh-1	0
NC_021780.1_Salmonella_phage_FSL_SP-088	0
NC_052997.1_Salmonella_phage_ST-118	0
NC_048648.1_Salmonella_phage_ST-101	0
NC_052998.1_Salmonella_phage_ST-374	0
NC_019417.1_Salmonella_phage_SPN19	0
NC_053002.1_Escherichia_phage_Utah	0
NC_053003.1_Salmonella_phage_vB_SalS_ABTNLsp1	0
NC_021783.1_Salmonella_phage_iEPS5	0
NC_053004.1_Salmonella_phage_TS13	0
NC_053005.1_Enterobacter_phage_KNP7	0
NC_053006.1_Salmonella_phage_ER3	0
NC_053007.1_Salmonella_phage_ER6	0
NC_048632.1_Salmonella_phage_35	0
NC_052992.1_Salmonella_phage_KNP6	0
NC_052993.1_Salmonella_phage_ER19	0
NC_052994.1_Salmonella_phage_BPS1	0
NC_048727.1_Salmonella_phage_Siskin	0
NC_052995.1_Salmonella_phage_BPS2	0
NC_052996.1_Salmonella_phage_BPS4	0
NC_052991.1_Serratia_phage_KpYy_1_41	0
NC_021779.1_Salmonella_phage_FSL_SP-030	0
NC_052990.1_Salmonella_phage_Season12	0
NC_053013.1_Pseudomonas_phage_vB_Pae-SS2019XI	0
NC_042344.1_Phage_Sano	0
NC_042345.1_Phage_Salvo	0
NC_052973.1_Xylella_phage_Bacata	0
NC_052976.1_Pseudomonas_phage_PspYZU01	0
NC_007024.1_Xanthomonas_phage_Xp15	0
NC_028770.1_Pseudomonas_phage_PaMx11	0
NC_042115.1_Pseudomonas_phage_vB_PaeS_PAO1_Ab19	0
NC_026594.1_Pseudomonas_phage_vB_PaeS_PAO1_Ab18	0

Supplementary Table S10. Intergenomic similarities of RefSeq phage genomes to vB_Kpn_ZCKp20p, calculated by VIRIDIC

Genome	Intergenomic Similarity%
NC_052965.1_Pseudomonas_phage_ZC01	0
NC_004813.1_Enterobacteria_phage_BP-4795	0
NC_049942.1_Escherichia_phage_JLK-2012	0
NC_011356.1_Enterobacteria_phage_YYZ-2008	0
NC_009514.1_Phage_cdtI_DNA	0
NC_049946.1_Escherichia_virus_Lambda_4A7	0
NC_049947.1_Escherichia_virus_Lambda_1H12	0
NC_049949.1_Escherichia_phage_Lambda_ev207	0
NC_019723.1_Enterobacteria_phage_HK630	0
NC_042057.1_Enterobacteria_phage_DE3	0
NC_001416.1_Enterobacteria_phage_lambda	0
NC_049951.1_Enterobacteria_phage_O276	0
NC_049952.1_Escherichia_virus_Lambda_2B8	0
NC_049953.1_Escherichia_phage_Lambda_ev099	0
NC_049954.1_Escherichia_virus_Lambda_2G7b	0
NC_049955.1_Escherichia_phage_Lambda_ev243	0
NC_002166.1_Bacteriophage_HK022	0
NC_019768.1_Enterobacteria_phage_HK106	0
NC_019704.1_Enterobacteria_phage_mEp237	0
NC_048803.1_Proteus_phage_Myduc	0
NC_019504.1_Erwinia_phage_vB_EamM-Y2	0
NC_025459.1_Aeromonas_phage_pAh6-C	0
NC_023594.2_Shewanella_phage_Spp001	0
NC_047824.1_Shewanella_phage_SppYZU05	0
NC_028879.1_Pseudomonas_phage_PaMx42	0
NC_024381.1_Pseudomonas_phage_vB_PaeS_SCH_Ab26	0
NC_017864.1_Pseudomonas_phage_vB_Pae-Kakheti25	0
NC_007806.1_Pseudomonas_phage_73	0
NC_023590.1_Acinetobacter_phage_IME_AB3	0
NC_042137.1_Acinetobacter_phage_vB_AbaS_Loki	0
NC_027378.1_Escherichia_phage_Seurat	0
NC_028776.1_Enterobacteria_phage_CAjan	0
NC_028997.1_Enterobacteria_phage_JenP2	0
NC_026610.2_Vibrio_phage_VpKK5	0
NC_048878.1_Alteromonas_phage_vB_AcoS-R7M	0
NC_031058.1_Pseudomonas_phage_NP1	0
NC_041953.1_Pseudomonas_phage_PaMx25	0
NC_048687.1_Pseudomonas_phage_PMBT14	0
NC_048763.1_Salmonella_phage_SE13	0
NC_048864.1_Salmonella_phage_birk	0
NC_048863.1_Salmonella_phage_yarpen	0

Supplementary Table S10. Intergenomic similarities of RefSeq phage genomes to vB_Kpn_ZCKp20p, calculated by VIRIDIC

Genome	Intergenomic Similarity%
NC_031250.1_Salmonella_phage_BP63	0
NC_048649.1_Salmonella_phage_UPF_BP2	0
NC_048179.1_Salmonella_phage_ZCSE2	0
NC_048764.1_Salmonella_phage_SE4	0
NC_019522.1_Pectobacterium_phage_ZF40	0
NC_003907.2_Vibrio_phage_VpV262	0
NC_021300.1_Pseudoalteromonas_phage_RIO-1	0
NC_048630.1_Pseudoalteromonas_phage_HP1	0
NC_025466.1_Shewanella_sp._phage_3/49	0
NC_021775.1_Salmonella_phage_FSL_SP-031	0
NC_026017.1_Salmonella_phage_LSPA1	0
NC_009232.2_Salmonella_phage_SETP3	0
NC_041991.1_Salmonella_phage_vB_SenS_AG11	0
NC_022754.1_Salmonella_phage_SETP7	0
NC_023608.1_Salmonella_phage_vB_SenS-Ent2	0
NC_019539.1_Salmonella_phage_vB_SenS-Ent1	0
NC_024204.1_Salmonella_phage_vB_SenS-Ent3	0
NC_028698.1_Salmonella_phage_f18SE	0
NC_031021.1_Salmonella_phage_MA12	0
NC_031925.1_Salmonella_phage_BPS11Q3	0
NC_016763.1_Salmonella_phage_SE2	0
NC_006940.2_Salmonella_phage_SS3e	0
NC_041992.1_Salmonella_phage_wksl3	0
NC_041898.1_Escherichia_phage_K1ind2	0
NC_030923.1_Pseudomonas_phage_YMC11/06/C171_PPU_BP	0
NC_029081.1_Pectobacterium_phage_Peat1	0
NC_041854.1_Pectobacterium_phage_PhiM1	0
NC_048117.1_Pectobacterium_phage_Khlen	0
NC_048061.1_Pectobacterium_phage_Nobby	0
NC_048116.1_Pectobacterium_phage_Clickz	0
NC_048118.1_Pectobacterium_phage_Koot	0
NC_031096.1_Pectobacterium_phage_PP90	0
NC_048120.1_Pectobacterium_phage_Zenivior	0
NC_048119.1_Pectobacterium_phage_Phoria	0
NC_042131.1_Pectobacterium_phage_vB_PatP_CB5	0
NC_048056.1_Pectobacterium_phage_Gaspode	0
NC_048058.1_Pectobacterium_phage_Lelidair	0
NC_047759.1_Dickeya_phage_BF25/12	0
NC_031068.2_Pectobacterium_phage_PP16	0
NC_048103.1_Pectobacterium_phage_PPWS2_DNA	0
NC_048148.1_Providencia_phage_vB_PstP_PS3	0

Supplementary Table S10. Intergenomic similarities of RefSeq phage genomes to vB_Kpn_ZCKp20p, calculated by VIRIDIC

Genome	Intergenomic Similarity%
NC_027342.1_Proteus_phage_PM16	0
NC_027363.1_Proteus_phage_PM_75	0
NC_048167.1_Vibrio_phage_vB_VpaS_OWB	0
NC_012662.1_Vibrio_phage_VP93	0
NC_048035.1_Vibrio_phage_vB_VpaP_KF1	0
NC_048036.1_Vibrio_phage_vB_VpaP_KF2	0
NC_019454.1_Pantoea_phage_LIMElight	0
NC_047929.1_Shigella_phage_SFN6B	0
NC_047787.1_Klebsiella_phage_KPV811	0
NC_048172.1_Escherichia_phage_Minorna	0
NC_023567.2_Klebsiella_phage_F19	0
NC_047811.1_Klebsiella_phage_vB_KpnP_KpV74	0
NC_047849.1_Klebsiella_phage_AltoGao	0
NC_028664.1_Klebsiella_phage_vB_Kp2	0
NC_047748.1_Klebsiella_phage_phiBO1E	0
NC_013649.2_Klebsiella_phage_KP34	0
NC_028816.1_Klebsiella_phage_vB_KpnP_SU503	0
NC_028670.1_Klebsiella_phage_vB_KpnP_KpV41	0
NC_025418.1_Klebsiella_phage_NTUH-K2044-K1-1_DNA	0
NC_031025.1_Klebsiella_phage_vB_KpnP_KpV475	0
NC_031246.1_Klebsiella_phage_vB_KpnP_KpV71	0
NC_047783.1_Klebsiella_phage_vB_KpnP_KpV48	0
NC_047944.1_Klebsiella_phage_myPSH1235	0
NC_047779.1_Klebsiella_phage_KP-Rio/2015	0
NC_028870.1_Klebsiella_phage_vB_KpnP_SU552A	0
NC_048174.1_Shigella_phage_HRP29	0
NC_015585.1_Pantoea_phage_LIMEzero	0
NC_025445.1_Enterobacteria_phage_J8-65	0
NC_048177.1_Escherichia_phage_Lidtsur	0
NC_047933.1_Pseudomonas_phage_phiNV3	0
NC_009935.1_Pseudomonas_phage_LKD16	0
NC_012418.1_Pseudomonas_phage_phikF77	0
NC_022091.1_Pseudomonas_phage_MPK7	0
NC_027375.1_Pseudomonas_phage_vB_PaeP_PPA-ABTNL	0
NC_047967.1_Pseudomonas_phage_vB_PaeP_PAO1_1-15pyo	0
NC_011105.1_Pseudomonas_phage_PT5	0
NC_017865.1_Pseudomonas_phage_vB_Pae-TbilisiM32	0
NC_047852.1_Pseudomonas_phage_phiNFS	0
NC_005045.1_Pseudomonas_phage_phiKMV	0
NC_011107.1_Pseudomonas_phage_PT2	0
NC_026602.1_Pseudomonas_phage_vB_PaeP_PAO1_Ab05	0

Supplementary Table S10. Intergenomic similarities of RefSeq phage genomes to vB_Kpn_ZCKp20p, calculated by VIRIDIC

Genome	Intergenomic Similarity%
NC_047953.1_Pseudomonas_phage_vB_PaeP_130_113	0
NC_010326.1_Pseudomonas_phage_LUZ19	0
NC_022746.1_Pseudomonas_phage_MPK6	0
NC_047952.1_Pseudomonas_phage_PAXYB1	0
NC_028836.1_Pseudomonas_phage_DL62	0
NC_048168.1_Pseudomonas_phage_RLP	0
NC_009936.1_Pseudomonas_phage_LKA1	0
NC_013638.1_Pseudomonas_phage_phi-2	0
NC_028684.1_Acinetobacter_phage_vB_AbaP_PD-6A3	0
NC_047896.1_Acinetobacter_phage_SWH-Ab-1	0
NC_028679.1_Acinetobacter_phage_vB_AbaP_PD-AB9	0
NC_041905.1_Acinetobacter_phage_SH-Ab_15519	0
NC_041967.1_Acinetobacter_phage_WCHABP5	0
NC_031086.1_Acinetobacter_phage_phiAB6	0
NC_042004.1_Acinetobacter_phage_vB_AbaP_B3	0
NC_028675.1_Acinetobacter_phage_phiAB1	0
NC_021316.1_Acinetobacter_phage_Abp1	0
NC_047883.1_Acinetobacter_phage_SWH-Ab-3	0
NC_028848.1_Acinetobacter_phage_Fri1	0
NC_041915.1_Acinetobacter_phage_vB_AbaP_AS11	0
NC_041914.1_Acinetobacter_phage_vB_AbaP_AS12	0
NC_048081.1_Acinetobacter_phage_vB_AbaP_B09_Aci08	0
NC_042006.1_Acinetobacter_phage_vB_ApiP_P1	0
NC_042007.1_Acinetobacter_phage_vB_ApiP_P2	0
NC_042003.1_Acinetobacter_phage_vB_AbaP_B1	0
NC_042005.1_Acinetobacter_phage_vB_AbaP_B5	0
NC_048076.1_Acinetobacter_phage_vB_AbaP_46-62_Aci07	0
NC_028987.2_Acinetobacter_phage_IME200	0
NC_048142.1_Acinetobacter_phage_AbKT21phiIII	0
NC_023570.1_Acinetobacter_phage_Petty	0
NC_025457.1_Acinetobacter_phage_vB_AbaP_Acibel007	0
NC_047741.1_Vibrio_phage_JSF7	0
NC_047834.1_Alteromonas_virus_vB_AspP-H4/4	0
NC_019520.1_Escherichia_phage_phiKT	0
NC_047890.1_Escherichia_phage_PGT2	0
NC_047941.1_Salmonella_phage_vB_SpuP_Spp16	0
NC_047865.1_Aeromonas_phage_CF7	0
NC_047866.1_Aeromonas_phage_Ahp1	0
NC_019528.1_Aeromonas_phage_phiAS7	0
NC_047966.1_Aeromonas_phage_25AhydR2PP	0
NC_028850.1_Yersinia_phage_vB_YenP_ISAO8	0

Supplementary Table S10. Intergenomic similarities of RefSeq phage genomes to vB_Kpn_ZCKp20p, calculated by VIRIDIC

Genome	Intergenomic Similarity%
NC_047951.1_Yersinia_phage_phiR8-01	0
NC_019911.2_Yersinia_phage_phi80-18	0
NC_047805.1_Yersinia_phage_fHe-Yen3-01	0
NC_047778.1_Pectobacterium_phage_PP2	0
NC_048111.1_Pectobacterium_phage_Arno160	0
NC_020078.1_Cronobacter_sakazakii_phage_vB_CskP_GAP227	0
NC_029070.1_Cronobacter_phage_Dev-CD-23823	0
NC_047917.1_Erwinia_phage_vB_EamP-S2	0
NC_009014.1_Erwinia_amylovora_phage_Era103	0
NC_019926.1_Erwinia_amylovora_phage_phiEa100	0
NC_019542.1_Pectobacterium_phage_PP1	0
NC_025450.1_Lelliottia_phage_phD2B	0
NC_019403.1_Enterobacteria_phage_vB_EcoP_ACG-C91	0
NC_047889.1_Escherichia_virus_mutPK1A2	0
NC_007637.1_Enterobacteria_phage_K1E	0
NC_047806.1_Escherichia_phage_vB_EcoP_B	0
NC_047807.1_Escherichia_phage_vB_EcoP_C	0
NC_047899.1_Escherichia_virus_VEc3	0
NC_008152.1_Enterobacteria_phage_K1-5	0
NC_047769.1_Escherichia_virus_AAPEc6	0
NC_047920.1_Citrobacter_phage_vB_CroP_CrRp3	0
NC_048064.1_Escherichia_phage_LL11	0
NC_004831.2_Enterobacteria_phage_SP6	0
NC_020414.2_Salmonella_phage_UAB_Phi78	0
NC_031271.1_Salmonella_phage_BP12B	0
NC_047858.1_Proteus_phage_PM_116	0
NC_027379.1_Proteus_phage_PM_85	0
NC_027390.1_Proteus_phage_PM_93	0
NC_028916.1_Proteus_phage_vB_PmiP_Pm5460	0
NC_047802.1_Pectobacterium_phage_PP99	0
NC_047956.1_Pseudomonas_phage_Achelous	0
NC_047955.1_Pseudomonas_phage_Nerthus	0
NC_047957.1_Pseudomonas_phage_Alpheus	0
NC_047894.1_Pseudomonas_phage_uligo	0
NC_047954.1_Pseudomonas_phage_Njord	0
NC_047903.1_Vibrio_phage_VEN	0
NC_047746.1_Vibrio_phage_Vc1	0
NC_047803.1_Vibrio_phage_Vp670	0
NC_025822.1_Vibrio_phage_phi-A318	0
NC_047737.1_Vibrio_phage_AS51	0
NC_009543.1_Xanthomonas_phage_Xop411	0

Supplementary Table S10. Intergenomic similarities of RefSeq phage genomes to vB_Kpn_ZCKp20p, calculated by VIRIDIC

Genome	Intergenomic Similarity%
NC_012742.1_Xanthomonas_phage_phiL7	0
NC_019933.2_Xanthomonas_phage_CP1_DNA	0
NC_022982.1_Xylella_phage_Paz	0
NC_022987.1_Xylella_phage_Prado	0
NC_047762.1_Xanthomonas_phage_XAJ24	0
NC_047840.1_Xanthomonas_phage_phi_Xc10	0
NC_030928.1_Xanthomonas_phage_f20-Xaj	0
NC_030937.1_Xanthomonas_phage_f30-Xaj	0
NC_041885.1_Pseudomonas_phage_VSW-3	0
NC_042104.1_Pseudomonas_phage_PollyC	0
NC_016764.1_Pseudomonas_phage_Bf7	0
NC_031014.1_Pseudomonas_phage_Andromeda	0
NC_041925.1_Proteus_phage_VB_PmiS-Isfahan	0
NC_042103.1_Pseudomonas_phage_Bjorn	0
NC_017971.2_Pseudomonas_phage_tf	0
NC_018850.2_Pseudomonas_phage_UFV-P2	0
NC_042107.1_Pseudomonas_phage_NV1	0
NC_028933.1_Pseudomonas_phage_PhiCHU	0
NC_004466.2_Pseudomonas_aeruginosa_phage_PaP3	0
NC_019813.1_Pseudomonas_phage_vB_PaeP_p2-10_Or1	0
NC_026599.1_Pseudomonas_phage_vB_PaeP_C2-10_Ab22	0
NC_010325.1_Pseudomonas_phage_LUZ24	0
NC_023583.1_Pseudomonas_phage_TL	0
NC_042343.1_Pseudomonas_phage_PaP4	0
NC_022971.1_Pseudomonas_phage_phiIBB-PAA2	0
NC_028919.1_Pseudomonas_phage_DL54	0
NC_019457.1_Vibrio_phage_CP-T1	0
NC_020082.1_Edwardsiella_phage_MSW-3_DNA	0
NC_021342.2_Edwardsiella_phage_PEi21	0
NC_041900.1_Klebsiella_phage_vB_KpnM_KpV52	0
NC_020204.1_Klebsiella_phage_JD001	0
NC_042041.1_Klebsiella_phage_vB_KpnM_KpV79	0
NC_023555.1_Edwardsiella_phage_eiAU-183	0
NC_042029.1_Edwardsiella_phage_eiAU	0
NC_016566.1_Shigella_phage_EP23	0
NC_013600.1_Sodalis_phage_SO-1	0
NC_024783.1_Enterobacteria_phage_EK99P-1	0
NC_028995.1_Acinetobacter_phage_phiAC-1	0
NC_017984.1_Acinetobacter_bacteriophage_AP22	0
NC_042028.1_Acinetobacter_phage_AB1	0
NC_041998.1_Acinetobacter_phage_AbP2	0

Supplementary Table S10. Intergenomic similarities of RefSeq phage genomes to vB_Kpn_ZCKp20p, calculated by VIRIDIC

Genome	Intergenomic Similarity%
NC_031117.1_Acinetobacter_phage_LZ35	0
NC_041857.1_Acinetobacter_phage_IME-AB2	0
NC_024785.1_Acinetobacter_phage_YMC-13-01-C62	0
NC_028855.1_Acinetobacter_phage_YMC11/12/R2315	0
NC_031098.1_Acinetobacter_phage_vB_AbaS_TRS1	0
NC_019541.1_Acinetobacter_phage_YMC/09/02/B1251_ABA_BP	0
NC_041866.1_Acinetobacter_phage_YMC11/11/R3177	0
NC_031908.1_Pseudoalteromonas_phage_PH1	0
NC_019420.1_Edwardsiella_phage_KF-1_DNA	0
NC_015158.1_Vibrio_phage_ICP2	0
NC_024791.1_Vibrio_phage_ICP2_2013_A_Haiti	0
NC_027988.2_Citrobacter_phage_CVT22	0
NC_007808.1_Pseudomonas_phage_PA11	0
NC_024365.1_Pseudomonas_phage_phiPSA1	0
NC_030931.1_Pseudomonas_phage_phi2	0
NC_031091.1_Pseudomonas_phage_MD8	0
NC_023575.1_Pseudomonas_phage_vB_PaeP_Tr60_Ab31	0
NC_007902.1_Sodalis_phage_phiSG1	0
NC_004827.1_Bacteriophage_Aaphi23	0
NC_013597.1_Aggregatibacter_phage_S1249	0
NC_025458.1_Shewanella_sp._phage_1/41	0
NC_004313.1_Salmonella_phage_ST64B	0
NC_021857.1_Shigella_phage_SfII	0
NC_021068.1_Vibrio_phage_douglas_12A4	0
NC_020844.1_Salicola_phage_CGphi29	0
NC_019923.1_Pseudomonas_phage_AF	0
NC_011589.1_Stenotrophomonas_phage_S1	0
NC_001901.1_Bacteriophage_N15	0
NC_001901.1_Bacteriophage_N15	0

frontiers

Supplementary Table S11. CoreGene5-identified homologous genes of phage vB_Kpn_ZCKp20p and top-matched phages by BLASTn (31 homologous)

Klebsiella phage vB_Kpn_ZCKp20p	Klebsiella phage ZCKP8	Klebsiella phage 6991	Vibrio phage pYD38-A	Aeromonas phage pIS4-A	Klebsiella phage VLCpiS13a	Klebsiella phage BUCT610	Klebsiella phage vB_KpnS_MK54	Klebsiella phage VLCpiS13f	Klebsiella phage vB_KpnS_ZX4	Klebsiella virus KpV2811	Klebsiella phage VLCpiS13e	Flyfo siphovirus Tbat1_6	Klebsiella phage BUCT541	Klebsiella phage VLCpiS13c
**OP373729.1**	**MZ440881.1**	**OL362277.1**	**NC_021534.1**	**NC_042037.1**	**ON602738.1**	**MZ318367.1**	**MW119258.1**	**ON602730.1**	**NC_054654.1**	**NC_054653.1**	**ON602759.1**	**OL617041.1**	**MZ836210.1**	**ON602737.1**
UXQ88383.1	QYW02877.1	URY99606.1	YP_008126213.1	YP_009614601.1	UVX30255.1	QWX10344.1	QZD26098.1	UVX29578.1	YP_010054559.1	YP_010054492.1	UVX31619.1	UIW10248.1	UAW06819.1	UVX30173.1
Product:hypothetical protein	Product:hypothetical protein	Product:hypothetical protein	Product:hypothetical protein	Product:hypothetical protein	Product:hypothetical protein	Product:hypothetical protein	Product:hypothetical protein	Product:hypothetical protein	Product:hypothetical protein	Product:hypothetical protein	Product:hypothetical protein	Product:hypothetical protein	Product:hypothetical protein	Product:hypothetical protein
UXQ88392.1	QYW02885.1	URY99535.1	YP_008126205.1	YP_009614609.1	UVX30262.1	QWX10350.1	QZD26106.1	UVX29571.1	YP_010054563.1	YP_010054486.1	UVX31626.1	UIW10230.1	UAW06815.1	UVX30164.1
Product:hypothetical protein	Product:hypothetical protein	Product:hypothetical protein	Product:hypothetical protein	Product:hypothetical protein	Product:hypothetical protein	Product:hypothetical protein	Product:hypothetical protein	Product:hypothetical protein	Product:hypothetical protein	Product:hypothetical protein	Product:hypothetical protein	Product:hypothetical protein	Product:hypothetical protein	Product:hypothetical protein
UXQ88393.1	QYW02886.1	URY99536.1	YP_008126204.1	YP_009614610.1	UVX30263.1	QWX10352.1	QZD26107.1	UVX29570.1	YP_010054565.1	YP_010054485.1	UVX31628.1	UIW10204.1	UAW06891.1	UVX30162.1
Product:terminase large subunit	Product:large terminase subunit	Product:terminase large subunit	Product:large terminase subunit	Product:terminase	Product:terminase large subunit	Product:large terminase subunit	Product:large terminase subunit	Product:terminase large subunit	Product:PBSX family terminase large subunit	Product:terminase large subunit	Product:terminase large subunit	Product:terminase large subunit	Product:large terminase	Product:terminase large subunit
UXQ88394.1	QYW02928.1	URY99538.1	YP_008126203.1	YP_009614611.1	UVX30264.1	QWX10271.1	QZD26108.1	UVX29568.1	YP_010054494.1	YP_010054484.1	UVX31630.1	UIW10242.1	UAW06890.1	UVX30161.1
Product:hypothetical protein	Product:hypothetical protein	Product:hypothetical protein	Product:hypothetical protein	Product:hypothetical protein	Product:hypothetical protein	Product:hypothetical protein	Product:hypothetical protein	Product:hypothetical protein	Product:hypothetical protein	Product:hypothetical protein	Product:hypothetical protein	Product:hypothetical protein	Product:hypothetical protein	Product:hypothetical protein
UXQ88395.1	QYW02887.1	URY99539.1	YP_008126202.1	YP_009614612.1	UVX30265.1	QWX10272.1	QZD26109.1	UVX29567.1	YP_010054495.1	YP_010054483.1	UVX31631.1	UIW10258.1	UAW06889.1	UVX30160.1
Product:hypothetical protein	Product:hypothetical protein	Product:hypothetical protein	Product:hypothetical protein	Product:hypothetical protein	Product:hypothetical protein	Product:hypothetical protein	Product:hypothetical protein	Product:hypothetical protein	Product:hypothetical protein	Product:hypothetical protein	Product:hypothetical protein	Product:hypothetical protein	Product:hypothetical protein	Product:hypothetical protein
UXQ88401.1	QYW02893.1	URY99544.1	YP_008126197.1	YP_009614617.1	UVX30272.1	QWX10279.1	QZD26115.1	UVX29561.1	YP_010054497.1	YP_010054478.1	UVX31635.1	UIW10203.1	UAW06885.1	UVX30155.1
Product:portal (connector) protein	Product:portal (connector) protein	Product:portal (connector) protein	Product:DUF1073 domain-containing protein	Product:DUF1073 domain-containing protein	Product:portal protein	Product:portal protein	Product:portal protein	Product:portal protein	Product:DUF1073 domain-containing protein	Product:DUF1073 domain-containing protein	Product:portal protein	Product:protein of unknown function DUF1073	Product:hypothetical protein	Product:portal protein
UXQ88402.1	QYW02895.1	URY99545.1	YP_008126196.1	YP_009614618.1	UVX30273.1	QWX10280.1	QZD26116.1	UVX29560.1	YP_010054498.1	YP_010054477.1	UVX31636.1	UIW10207.1	UAW06884.1	UVX30153.1
Product:minor capsid protein	Product:head decoration protein	Product:hypothetical protein	Product:hypothetical protein	Product:hypothetical protein	Product:head morphogenesis	Product:hypothetical protein	Product:minor capsid protein	Product:head morphogenesis	Product:hypothetical protein	Product:hypothetical protein	Product:head morphogenesis	Product:hypothetical protein	Product:hypothetical protein	Product:head morphogenesis
UXQ88406.1	QYW02932.1	URY99549.1	YP_008126193.1	YP_009614621.1	UVX30276.1	QWX10284.1	QZD26120.1	UVX29557.1	YP_010054502.1	YP_010054472.1	UVX31640.1	UIW10244.1	UAW06881.1	UVX30150.1
Product:hypothetical protein	Product:hypothetical protein	Product:hypothetical protein	Product:hypothetical protein	Product:hypothetical protein	Product:hypothetical protein	Product:hypothetical protein	Product:hypothetical protein	Product:hypothetical protein	Product:hypothetical protein	Product:hypothetical protein	Product:hypothetical protein	Product:hypothetical protein	Product:hypothetical protein	Product:hypothetical protein
UXQ88407.1	QYW02896.1	URY99550.1	YP_008126192.1	YP_009614622.1	UVX30277.1	QWX10285.1	QZD26122.1	UVX29556.1	YP_010054503.1	YP_010054471.1	UVX31641.1	UIW10247.1	UAW06880.1	UVX30149.1
Product:endolysin	Product:lysozyme	Product:lysozyme R	Product:lysozyme	Product:hypothetical protein	Product:endolysin	Product:lysozyme	Product:lysozyme	Product:endolysin	Product:lysozyme	Product:lysozyme	Product:endolysin	Product:hypothetical protein	Product:lysozyme	Product:endolysin

Supplementary Table S11. CoreGene5-identified homologous genes of phage vB_Kpn_ZCKp20p and top-matched phages by BLASTn (31 homologous)

Klebsiella phage vB_Kpn_ZCKp20p	Klebsiella phage ZCKP8	Klebsiella phage 6991	Vibrio phage pYD38-A	Aeromonas phage pIS4-A	Klebsiella phage VLCpiS13a	Klebsiella phage BUCT610	Klebsiella phage vB_KpnS_MK54	Klebsiella phage VLCpiS13f	Klebsiella phage vB_KpnS_ZX4	Klebsiella virus KpV2811	Klebsiella phage VLCpiS13e	Flyfo siphovirus Tbat1_6	Klebsiella phage BUCT541	Klebsiella phage VLCpiS13c
**OP373729.1**	**MZ440881.1**	**OL362277.1**	**NC_021534.1**	**NC_042037.1**	**ON602738.1**	**MZ318367.1**	**MW119258.1**	**ON602730.1**	**NC_054654.1**	**NC_054653.1**	**ON602759.1**	**OL617041.1**	**MZ836210.1**	**ON602737.1**
UXQ88413.1	QYW02936.1	URY99554.1	YP_008126189.1	YP_009614625.1	UVX30283.1	QWX10290.1	QZD26047.1	UVX29549.1	YP_010054507.1	YP_010054466.1	UVX31647.1	UIW10220.1	UAW06874.1	UVX30143.1
Product:hypothetical protein	Product:hypothetical protein	Product:hypothetical protein	Product:hypothetical protein	Product:hypothetical protein	Product:hypothetical protein	Product:hypothetical protein	Product:hypothetical protein	Product:hypothetical protein	Product:hypothetical protein	Product:hypothetical protein	Product:hypothetical protein	Product:hypothetical protein	Product:hypothetical protein	Product:hypothetical protein
UXQ88415.1	QYW02937.1	URY99556.1	YP_008126187.1	YP_009614627.1	UVX30285.1	QWX10291.1	QZD26049.1	UVX29547.1	YP_010054508.1	YP_010054465.1	UVX31649.1	UIW10267.1	UAW06873.1	UVX30141.1
Product:hypothetical protein	Product:holin	Product:hypothetical protein	Product:hypothetical protein	Product:hypothetical protein	Product:hypothetical protein	Product:hypothetical protein	Product:hypothetical protein	Product:hypothetical protein	Product:hypothetical protein	Product:hypothetical protein	Product:hypothetical protein	Product:hypothetical protein	Product:hypothetical protein	Product:hypothetical protein
UXQ88416.1	QYW02899.1	URY99557.1	YP_008126186.1	YP_009614628.1	UVX30286.1	QWX10292.1	QZD26050.1	UVX29546.1	YP_010054509.1	YP_010054463.1	UVX31650.1	UIW10263.1	UAW06872.1	UVX30140.1
Product:hypothetical protein	Product:hypothetical protein	Product:hypothetical protein	Product:hypothetical protein	Product:hypothetical protein	Product:hypothetical protein	Product:hypothetical protein	Product:hypothetical protein	Product:hypothetical protein	Product:hypothetical protein	Product:hypothetical protein	Product:hypothetical protein	Product:hypothetical protein	Product:hypothetical protein	Product:hypothetical protein
UXQ88420.1	QYW02903.1	URY99561.1	YP_008126182.1	YP_009614632.1	UVX30210.1	QWX10296.1	QZD26053.1	UVX29542.1	YP_010054513.1	YP_010054459.1	UVX31653.1	UIW10226.1	UAW06868.1	UVX30136.1
Product:hypothetical protein	Product:hypothetical protein	Product:hypothetical protein	Product:hypothetical protein	Product:hypothetical protein	Product:MarR family transcrition regulator	Product:hypothetical protein	Product:hypothetical protein	Product:MarR family transcrition regulator	Product:hypothetical protein	Product:hypothetical protein	Product:MarR family transcrition regulator	Product:hypothetical protein	Product:transcription factor	Product:MarR family transcrition regulator
UXQ88422.1	QYW02905.1	URY99563.1	YP_008126180.1	YP_009614634.1	UVX30212.1	QWX10298.1	QZD26055.1	UVX29540.1	YP_010054515.1	YP_010054457.1	UVX31655.1	UIW10237.1	UAW06866.1	UVX30134.1
Product:hypothetical protein	Product:hypothetical protein	Product:hypothetical protein	Product:hypothetical protein	Product:hypothetical protein	Product:hypothetical protein	Product:hypothetical protein	Product:hypothetical protein	Product:hypothetical protein	Product:hypothetical protein	Product:hypothetical protein	Product:hypothetical protein	Product:hypothetical protein	Product:hypothetical protein	Product:hypothetical protein
UXQ88423.1	QYW02907.1	URY99564.1	YP_008126179.1	YP_009614635.1	UVX30213.1	QWX10299.1	QZD26056.1	UVX29539.1	YP_010054516.1	YP_010054456.1	UVX31656.1	UIW10205.1	UAW06865.1	UVX30133.1
Product:procapsid core protein serine protease	Product:hypothetical protein	Product:coat protein	Product:hypothetical protein	Product:hypothetical protein	Product:hypothetical protein	Product:coat protein	Product:prohead core protein serine protease	Product:hypothetical protein	Product:coat protein	Product:hypothetical protein	Product:hypothetical protein	Product:hypothetical protein	Product:hypothetical protein	Product:hypothetical protein
UXQ88425.1	QYW02909.1	URY99566.1	YP_008126177.1	YP_009614637.1	UVX30215.1	QWX10301.1	QZD26058.1	UVX29537.1	YP_010054518.1	YP_010054454.1	UVX31658.1	UIW10206.1	UAW06863.1	UVX30131.1
Product:major capsid protein	Product:major coat protein	Product:coat protein	Product:DUF6260 family protein	Product:DUF6260 family protein	Product:virion structural protein	Product:protein of unknown function DUF2184	Product:major capsid protein	Product:virion structural protein	Product:DUF6260 family protein	Product:hypothetical protein	Product:virion structural protein	Product:protein of unknown function DUF6260	Product:coat protein	Product:virion structural protein
UXQ88435.1	QYW02940.1	URY99571.1	YP_008126171.1	YP_009614643.1	UVX30220.1	QWX10306.1	QZD26062.1	UVX29534.1	YP_010054521.1	YP_010054450.1	UVX31662.1	UIW10276.1	UAW06859.1	UVX30205.1
Product:hypothetical protein	Product:hypothetical protein	Product:hypothetical protein	Product:hypothetical protein	Product:hypothetical protein	Product:hypothetical protein	Product:hypothetical protein	Product:hypothetical protein	Product:hypothetical protein	Product:hypothetical protein	Product:hypothetical protein	Product:hypothetical protein	Product:hypothetical protein	Product:hypothetical protein	Product:hypothetical protein
UXQ88431.1	QYW02942.1	URY99573.1	YP_008126170.1	YP_009614644.1	UVX30222.1	QWX10308.1	QZD26064.1	UVX29532.1	YP_010054524.1	YP_010054447.1	UVX31664.1	UIW10212.1	UAW06856.1	UVX30203.1

Supplementary Table S11. CoreGene5-identified homologous genes of phage vB_Kpn_ZCKp20p and top-matched phages by BLASTn (31 homologous)

Klebsiella phage vB_Kpn_ZCKp20p	Klebsiella phage ZCKP8	Klebsiella phage 6991	Vibrio phage pYD38-A	Aeromonas phage pIS4-A	Klebsiella phage VLCpiS13a	Klebsiella phage BUCT610	Klebsiella phage vB_KpnS_MK54	Klebsiella phage VLCpiS13f	Klebsiella phage vB_KpnS_ZX4	Klebsiella virus KpV2811	Klebsiella phage VLCpiS13e	Flyfo siphovirus Tbat1_6	Klebsiella phage BUCT541	Klebsiella phage VLCpiS13c
**OP373729.1**	**MZ440881.1**	**OL362277.1**	**NC_021534.1**	**NC_042037.1**	**ON602738.1**	**MZ318367.1**	**MW119258.1**	**ON602730.1**	**NC_054654.1**	**NC_054653.1**	**ON602759.1**	**OL617041.1**	**MZ836210.1**	**ON602737.1**
Product:hypothetical protein	Product:putative transcriptional regulator	Product:hypothetical protein	Product:hypothetical protein	Product:hypothetical protein	Product:hypothetical protein	Product:hypothetical protein	Product:putative transcriptional regulator	Product:ParB-like partition protein	Product:hypothetical protein	Product:ParB/RepB/Spo0J family partition protein	Product:ParB-like partition protein	Product:KorB domain protein	Product:hypothetical protein	Product:ParB-like partition protein
UXQ88434.1	QYW02912.1	URY99576.1	YP_008126168.1	YP_009614646.1	UVX30223.1	QWX10311.1	QZD26068.1	UVX29528.1	YP_010054527.1	YP_010054444.1	UVX31588.1	UIW10245.1	UAW06853.1	UVX30200.1
Product:holin protein	Product:hypothetical protein	Product:hypothetical protein	Product:hypothetical protein	Product:hypothetical protein	Product:membrane protein	Product:hypothetical protein	Product:holin	Product:membrane protein	Product:hypothetical protein	Product:hypothetical protein	Product:membrane protein	Product:hypothetical protein	Product:hypothetical protein	Product:membrane protein
UXQ88436.1	QYW02913.1	URY99577.1	YP_008126167.1	YP_009614647.1	UVX30224.1	QWX10312.1	QZD26069.1	UVX29527.1	YP_010054528.1	YP_010054443.1	UVX31589.1	UIW10235.1	UAW06852.1	UVX30199.1
Product:hypothetical protein	Product:hypothetical protein	Product:hypothetical protein	Product:hypothetical protein	Product:hypothetical protein	Product:hypothetical protein	Product:hypothetical protein	Product:head to tail adaptor	Product:hypothetical protein	Product:hypothetical protein	Product:hypothetical protein	Product:hypothetical protein	Product:hypothetical protein	Product:hypothetical protein	Product:hypothetical protein
UXQ88438.1	QYW02915.1	URY99579.1	YP_008126166.1	YP_009614648.1	UVX30226.1	QWX10313.1	QZD26070.1	UVX29526.1	YP_010054530.1	YP_010054441.1	UVX31591.1	UIW10236.1	UAW06850.1	UVX30197.1
Product:minor capsid protein	Product:hypothetical protein	Product:hypothetical protein	Product:hypothetical protein	Product:hypothetical protein	Product:hypothetical protein	Product:hypothetical protein	Product:minor capsid protein	Product:hypothetical protein	Product:hypothetical protein	Product:hypothetical protein	Product:hypothetical protein	Product:hypothetical protein	Product:minor capsid protein	Product:hypothetical protein
UXQ88439.1	QYW02916.1	URY99580.1	YP_008126165.1	YP_009614649.1	UVX30227.1	QWX10314.1	QZD26071.1	UVX29525.1	YP_010054531.1	YP_010054440.1	UVX31592.1	UIW10232.1	UAW06849.1	UVX30196.1
Product:minor capsid protein	Product:hypothetical protein	Product:hypothetical protein	Product:HK97 gp10 family protein	Product:HK97 gp10 family protein	Product:tail completion or Neck1 protein	Product:hypothetical protein	Product:minor capsid protein	Product:hypothetical protein	Product:HK97 gp10 family protein	Product:HK97 gp10 family protein	Product:hypothetical protein	Product:hypothetical protein	Product:hypothetical protein	Product:tail completion or Neck1 protein
UXQ88441.1	QYW02917.1	URY99581.1	YP_008126240.1	YP_009614650.1	UVX30228.1	QWX10315.1	QZD26072.1	UVX29524.1	YP_010054532.1	YP_010054439.1	UVX31593.1	UIW10234.1	UAW06848.1	UVX30195.1
Product:minor capsid protein	Product:hypothetical protein	Product:hypothetical protein	Product:hypothetical protein	Product:hypothetical protein	Product:hypothetical protein	Product:hypothetical protein	Product:tail completion protein	Product:hypothetical protein	Product:hypothetical protein	Product:hypothetical protein	Product:hypothetical protein	Product:hypothetical protein	Product:hypothetical protein	Product:hypothetical protein
UXQ88442.1	QYW02919.1	URY99583.1	YP_008126238.1	YP_009614652.1	UVX30229.1	QWX10318.1	QZD26073.1	UVX29523.1	YP_010054534.1	YP_010054437.1	UVX31595.1	UIW10213.1	UAW06845.1	UVX30194.1
Product:fibritin protein	Product:major tail subunit	Product:hypothetical protein	Product:major tail subunit	Product:hypothetical protein	Product:major tail protein	Product:hypothetical protein	Product:major tail subunit	Product:major tail protein	Product:hypothetical protein	Product:hypothetical protein	Product:major tail protein	Product:Immunoglobulin domain protein	Product:major tail protein	Product:major tail protein
UXQ88447.1	QYW02920.1	URY99588.1	YP_008126234.1	YP_009614656.1	UVX30234.1	QWX10324.1	QZD26077.1	UVX29518.1	YP_010054540.1	YP_010054430.1	UVX31600.1	UIW10216.1	UAW06839.1	UVX30189.1
Product:putative tail protein	Product:hypothetical protein	Product:hypothetical protein	Product:DUF6246 family protein	Product:DUF6246 family protein	Product:hypothetical protein	Product:hypothetical protein	Product:hypothetical protein	Product:hypothetical protein	Product:DUF6246 family protein	Product:DUF6246 family protein	Product:hypothetical protein	Product:protein of unknown function DUF6246	Product:hypothetical protein	Product:hypothetical protein
UXQ88448.1	QYW02921.1	URY99589.1	YP_008126233.1	YP_009614657.1	UVX30235.1	QWX10325.1	QZD26078.1	UVX29517.1	YP_010054541.1	YP_010054429.1	UVX31601.1	UIW10199.1	UAW06838.1	UVX30188.1

Supplementary Table S11. CoreGene5-identified homologous genes of phage vB_Kpn_ZCKp20p and top-matched phages by BLASTn (31 homologous)

Klebsiella phage vB_Kpn_ZCKp20p	Klebsiella phage ZCKP8	Klebsiella phage 6991	Vibrio phage pYD38-A	Aeromonas phage pIS4-A	Klebsiella phage VLCpiS13a	Klebsiella phage BUCT610	Klebsiella phage vB_KpnS_MK54	Klebsiella phage VLCpiS13f	Klebsiella phage vB_KpnS_ZX4	Klebsiella virus KpV2811	Klebsiella phage VLCpiS13e	Flyfo siphovirus Tbat1_6	Klebsiella phage BUCT541	Klebsiella phage VLCpiS13c
**OP373729.1**	**MZ440881.1**	**OL362277.1**	**NC_021534.1**	**NC_042037.1**	**ON602738.1**	**MZ318367.1**	**MW119258.1**	**ON602730.1**	**NC_054654.1**	**NC_054653.1**	**ON602759.1**	**OL617041.1**	**MZ836210.1**	**ON602737.1**
Product:tail tape measure protein	Product:tail length tape measure protein	Product:tail length tape-measure protein T	Product:tail length tape measure protein	Product:tail length tape measure protein	Product:tail length tape measure protein	Product:tail length tape measure protein	Product:putative tail protein	Product:tail length tape measure protein	Product:tail length tape-measure protein 1	Product:tail length tape-measure protein	Product:tail length tape measure protein	Product:tape measure protein	Product:tail length tape-measure protein 1	Product:tail length tape measure protein
UXQ88449.1	QYW02922.1	URY99590.1	YP_008126232.1	YP_009614658.1	UVX30238.1	QWX10326.1	QZD26079.1	UVX29515.1	YP_010054542.1	YP_010054428.1	UVX31603.1	UIW10225.1	UAW06837.1	UVX30187.1
Product:baseplate wedge protein	Product:minor tail protein	Product:hypothetical protein	Product:hypothetical protein	Product:hypothetical protein	Product:virion structural protein	Product:hypothetical protein	Product:minor tail protein	Product:virion structural protein	Product:hypothetical protein	Product:hypothetical protein	Product:virion structural protein	Product:hypothetical protein	Product:hypothetical protein	Product:virion structural protein
UXQ88450.1	QYW02923.1	URY99591.1	YP_008126231.1	YP_009614659.1	UVX30239.1	QWX10327.1	QZD26080.1	UVX29514.1	YP_010054543.1	YP_010054427.1	UVX31604.1	UIW10227.1	UAW06836.1	UVX30186.1
Product:hypothetical protein	Product:hypothetical protein	Product:hypothetical protein	Product:hypothetical protein	Product:hypothetical protein	Product:minor tail protein	Product:hypothetical protein	Product:hypothetical protein	Product:minor tail protein	Product:hypothetical protein	Product:hypothetical protein	Product:minor tail protein	Product:hypothetical protein	Product:hypothetical protein	Product:minor tail protein
UXQ88451.1	QYW02925.1	URY99593.1	YP_008126229.1	YP_009614661.1	UVX30241.1	QWX10329.1	QZD26082.1	UVX29512.1	YP_010054545.1	YP_010054426.1	UVX31606.1	UIW10198.1	UAW06834.1	UVX30184.1
Product:host specificity factor TipJ family tail	Product:putative tail protein	Product:hypothetical protein	Product:MoaD/ThiS family protein	Product:MoaD/ThiS family protein	Product:tail protein	Product:hypothetical protein	Product:tail protein	Product:tail protein	Product:MoaD/ThiS family protein	Product:MoaD/ThiS family protein	Product:tail protein	Product:hypothetical protein	Product:hypothetical protein	Product:tail protein
UXQ88454.1	QYW02950.1	URY99597.1	YP_008126226.1	YP_009614664.1	UVX30243.1	QWX10332.1	QZD26084.1	UVX29591.1	YP_010054547.1	YP_010054424.1	UVX31608.1	UIW10224.1	UAW06832.1	UVX30180.1
Product:single stranded DNA-binding protein	Product:single-stranded DNA-binding protein	Product:hypothetical protein	Product:single-stranded DNA-binding protein	Product:single-stranded DNA-binding protein	Product:single strand DNA binding protein	Product:single-stranded DNA-binding protein	Product:single-stranded DNA-binding protein	Product:single strand DNA binding protein	Product:single-stranded DNA-binding protein	Product:single-stranded DNA-binding protein	Product:single strand DNA binding protein	Product:single-strand binding protein family protein	Product:single strand binding protein	Product:single strand DNA binding protein
UXQ88460.1	QYW02955.1	URY99596.1	YP_008126221.1	YP_009614669.1	UVX30247.1	QWX10337.1	QZD26089.1	UVX29586.1	YP_010054552.1	YP_010054420.1	UVX31613.1	UIW10201.1	UAW06827.1	UVX30181.1
Product:DNA helicase	Product:DNA helicase	Product:ATP-dependent RNA helicase	Product:helicase	Product:helicase	Product:DNA helicase	Product:DNA helicase	Product:DNA helicase	Product:DNA helicase	Product:DEAD/DEAH box helicase family protein	Product:DEAD/DEAH box helicase family protein	Product:DNA helicase	Product:DNA helicase	Product:DEAD/DEAH box helicase family protein	Product:DNA helicase

Supplementary Table S12. CoreGene5-identified homologous genes of phage vB_Kpn_ZCKp20p, phage ZCKP8, and phage 6991 (64 homologs)

Klebsiella phage ZCKp20p	Klebsiella phage ZCKP8	Klebsiella phage 6991
OP373729.1	MZ440881.1	OL362277.1
PI:UXQ88382.1	PI:QYW02876.1	PI:URY99605.1
Product:hypothetical protein	Product:hypothetical protein	Product:hypothetical protein
PI:UXQ88383.1	PI:QYW02877.1	PI:URY99606.1
Product:hypothetical protein	Product:hypothetical protein	Product:hypothetical protein
PI:UXQ88384.1	PI:QYW02878.1	PI:URY99607.1
Product:hypothetical protein	Product:hypothetical protein	Product:hypothetical protein
PI:UXQ88385.1	PI:QYW02879.1	PI:URY99608.1
Product:hypothetical protein	Product:hypothetical protein	Product:hypothetical protein
PI:UXQ88386.1	PI:QYW02880.1	PI:URY99609.1
Product:hypothetical protein	Product:hypothetical protein	Product:hypothetical protein
PI:UXQ88387.1	PI:QYW02881.1	PI:URY99610.1
Product:hypothetical protein	Product:hypothetical protein	Product:hypothetical protein
PI:UXQ88388.1	PI:QYW02882.1	PI:URY99611.1
Product:hypothetical protein	Product:hypothetical protein	Product:EaA protein
PI:UXQ88390.1	PI:QYW02883.1	PI:URY99613.1
Product:hypothetical protein	Product:hypothetical protein	Product:hypothetical protein
PI:UXQ88391.1	PI:QYW02884.1	PI:URY99614.1
Product:hypothetical protein	Product:hypothetical protein	Product:hypothetical protein
PI:UXQ88392.1	PI:QYW02885.1	PI:URY99535.1
Product:hypothetical protein	Product:hypothetical protein	Product:hypothetical protein
PI:UXQ88393.1	PI:QYW02886.1	PI:URY99536.1
Product:terminase large subunit	Product:large terminase subunit	Product:terminase large subunit
PI:UXQ88394.1	PI:QYW02928.1	PI:URY99538.1
Product:hypothetical protein	Product:hypothetical protein	Product:hypothetical protein
PI:UXQ88395.1	PI:QYW02887.1	PI:URY99539.1
Product:hypothetical protein	Product:hypothetical protein	Product:hypothetical protein
PI:UXQ88396.1	PI:QYW02889.1	PI:URY99540.1
Product:hypothetical protein	Product:hypothetical protein	Product:hypothetical protein
PI:UXQ88398.1	PI:QYW02891.1	PI:URY99542.1
Product:hypothetical protein	Product:hypothetical protein	Product:hypothetical protein
PI:UXQ88401.1	PI:QYW02893.1	PI:URY99544.1
Product:portal (connector) protein	Product:portal (connector) protein	Product:portal (connector) protein
PI:UXQ88402.1	PI:QYW02895.1	PI:URY99545.1

Supplementary Table S12. CoreGene5-identified homologous genes of phage vB_Kpn_ZCKp20p, phage ZCKP8, and phage 6991 (64 homologs)

Klebsiella phage ZCKp20p	Klebsiella phage ZCKP8	Klebsiella phage 6991
**OP373729.1**	**MZ440881.1**	**OL362277.1**
Product:minor capsid protein	Product:head decoration protein	Product:hypothetical protein
PI:UXQ88404.1	PI:QYW02930.1	PI:URY99547.1
Product:hypothetical protein	Product:hypothetical protein	Product:hypothetical protein
PI:UXQ88405.1	PI:QYW02931.1	PI:URY99548.1
Product:hypothetical protein	Product:hypothetical protein	Product:hypothetical protein
PI:UXQ88406.1	PI:QYW02932.1	PI:URY99549.1
Product:hypothetical protein	Product:hypothetical protein	Product:hypothetical protein
PI:UXQ88407.1	PI:QYW02896.1	PI:URY99550.1
Product:endolysin	Product:lysozyme	Product:lysozyme R
PI:UXQ88408.1	PI:QYW02897.1	PI:URY99551.1
Product:holin protein	Product:putative holin	Product:hypothetical protein
PI:UXQ88409.1	PI:QYW02898.1	PI:URY99552.1
Product:endopeptidase Rz	Product:tail protein	Product:lysin
PI:UXQ88412.1	PI:QYW02934.1	PI:URY99553.1
Product:hypothetical protein	Product:hypothetical protein	Product:hypothetical protein
PI:UXQ88413.1	PI:QYW02936.1	PI:URY99554.1
Product:hypothetical protein	Product:hypothetical protein	Product:hypothetical protein
PI:UXQ88415.1	PI:QYW02937.1	PI:URY99556.1
Product:hypothetical protein	Product:holin	Product:hypothetical protein
PI:UXQ88416.1	PI:QYW02899.1	PI:URY99557.1
Product:hypothetical protein	Product:hypothetical protein	Product:hypothetical protein
PI:UXQ88417.1	PI:QYW02900.1	PI:URY99558.1
Product:hypothetical protein	Product:hypothetical protein	Product:hypothetical protein
PI:UXQ88418.1	PI:QYW02901.1	PI:URY99559.1
Product:hypothetical protein	Product:hypothetical protein	Product:hypothetical protein
PI:UXQ88419.1	PI:QYW02902.1	PI:URY99560.1
Product:zinc-binding domain (ZBD) of primase-helicase	Product:zinc-binding domain of primase-helicase	Product:hypothetical protein
PI:UXQ88420.1	PI:QYW02903.1	PI:URY99561.1
Product:hypothetical protein	Product:hypothetical protein	Product:hypothetical protein
PI:UXQ88421.1	PI:QYW02904.1	PI:URY99562.1
Product:hypothetical protein	Product:hypothetical protein	Product:hypothetical protein
PI:UXQ88422.1	PI:QYW02905.1	PI:URY99563.1
Product:hypothetical protein	Product:hypothetical protein	Product:hypothetical protein

frontiers

Supplementary Table S12. CoreGene5-identified homologous genes of phage vB_Kpn_ZCKp20p, phage ZCKP8, and phage 6991 (64 homologs)

Klebsiella phage ZCKp20p	Klebsiella phage ZCKP8	Klebsiella phage 6991
**OP373729.1**	**MZ440881.1**	**OL362277.1**
PI:UXQ88423.1	PI:QYW02907.1	PI:URY99564.1
Product:procapsid core protein serine protease	Product:hypothetical protein	Product:coat protein
PI:UXQ88424.1	PI:QYW02908.1	PI:URY99565.1
Product:capsid decoration protein	Product:capsid decoration protein	Product:hypothetical protein
PI:UXQ88425.1	PI:QYW02909.1	PI:URY99566.1
Product:major capsid protein	Product:major coat protein	Product:coat protein
PI:UXQ88426.1	PI:QYW02910.1	PI:URY99567.1
Product:hypothetical protein	Product:hypothetical protein	Product:hypothetical protein
PI:UXQ88427.1	PI:QYW02911.1	PI:URY99568.1
Product:hypothetical protein	Product:hypothetical protein	Product:hypothetical protein
PI:UXQ88428.1	PI:QYW02938.1	PI:URY99569.1
Product:hypothetical protein	Product:hypothetical protein	Product:hypothetical protein
PI:UXQ88429.1	PI:QYW02939.1	PI:URY99570.1
Product:DNA polymerase III beta subunit	Product:DNA polymerase III beta subunit	Product:DNA polymerase III beta subunit
PI:UXQ88435.1	PI:QYW02940.1	PI:URY99571.1
Product:hypothetical protein	Product:hypothetical protein	Product:hypothetical protein
PI:UXQ88430.1	PI:QYW02941.1	PI:URY99572.1
Product:hypothetical protein	Product:hypothetical protein	Product:hypothetical protein
PI:UXQ88431.1	PI:QYW02942.1	PI:URY99573.1
Product:hypothetical protein	Product:putative transcriptional regulator	Product:hypothetical protein
PI:UXQ88432.1	PI:QYW02943.1	PI:URY99574.1
Product:ParB/RepB/Spo0J family partition protein	Product:hypothetical protein	Product:hypothetical protein
PI:UXQ88433.1	PI:QYW02944.1	PI:URY99575.1
Product:hypothetical protein	Product:hypothetical protein	Product:hypothetical protein
PI:UXQ88434.1	PI:QYW02912.1	PI:URY99576.1
Product:holin protein	Product:hypothetical protein	Product:hypothetical protein
PI:UXQ88436.1	PI:QYW02913.1	PI:URY99577.1
Product:hypothetical protein	Product:hypothetical protein	Product:hypothetical protein
PI:UXQ88437.1	PI:QYW02914.1	PI:URY99578.1
Product:hypothetical protein	Product:hypothetical protein	Product:hypothetical protein
PI:UXQ88438.1	PI:QYW02915.1	PI:URY99579.1
Product:minor capsid protein	Product:hypothetical protein	Product:hypothetical protein
PI:UXQ88439.1	PI:QYW02916.1	PI:URY99580.1

Supplementary Table S12. CoreGene5-identified homologous genes of phage vB_Kpn_ZCKp20p, phage ZCKP8, and phage 6991 (64 homologs)

Klebsiella phage ZCKp20p	Klebsiella phage ZCKP8	Klebsiella phage 6991
**OP373729.1**	**MZ440881.1**	**OL362277.1**
Product:minor capsid protein	Product:hypothetical protein	Product:hypothetical protein
PI:UXQ88441.1	PI:QYW02917.1	PI:URY99581.1
Product:minor capsid protein	Product:hypothetical protein	Product:hypothetical protein
PI:UXQ88442.1	PI:QYW02919.1	PI:URY99583.1
Product:fibritin protein	Product:major tail subunit	Product:hypothetical protein
PI:UXQ88443.1	PI:QYW02946.1	PI:URY99584.1
Product:hypothetical protein	Product:hypothetical protein	Product:hypothetical protein
PI:UXQ88444.1	PI:QYW02947.1	PI:URY99585.1
Product:hypothetical protein	Product:hypothetical protein	Product:hypothetical protein
PI:UXQ88445.1	PI:QYW02948.1	PI:URY99586.1
Product:HNH homing endonuclease	Product:HNH-endonuclease	Product:HNH endonuclease motif protein
PI:UXQ88446.1	PI:QYW02949.1	PI:URY99587.1
Product:hypothetical protein	Product:hypothetical protein	Product:hypothetical protein
PI:UXQ88447.1	PI:QYW02920.1	PI:URY99588.1
Product:putative tail protein	Product:hypothetical protein	Product:hypothetical protein
PI:UXQ88448.1	PI:QYW02921.1	PI:URY99589.1
Product:tail tape measure protein	Product:tail length tape measure protein	Product:tail length tape-measure protein T
PI:UXQ88449.1	PI:QYW02922.1	PI:URY99590.1
Product:baseplate wedge protein	Product:minor tail protein	Product:hypothetical protein
PI:UXQ88450.1	PI:QYW02923.1	PI:URY99591.1
Product:hypothetical protein	Product:hypothetical protein	Product:hypothetical protein
PI:UXQ88451.1	PI:QYW02925.1	PI:URY99593.1
Product:host specificity factor TipJ family tail	Product:putative tail protein	Product:hypothetical protein
PI:UXQ88452.1	PI:QYW02926.1	PI:URY99594.1
Product:tailspike protein	Product:tail fiber protein	Product:hypothetical protein
PI:UXQ88454.1	PI:QYW02950.1	PI:URY99597.1
Product:single stranded DNA-binding protein	Product:single-stranded DNA-binding protein	Product:hypothetical protein
PI:UXQ88460.1	PI:QYW02955.1	PI:URY99596.1
Product:DNA helicase	Product:DNA helicase	Product:ATP-dependent RNA helicase
